# Evidence of cryptic introgression in tomato (*Solanum lycopersicum* L.) based on wild tomato species alleles

**DOI:** 10.1186/1471-2229-12-133

**Published:** 2012-08-07

**Authors:** Joanne A Labate, Larry D Robertson

**Affiliations:** 1 USDA-ARS Plant Genetic Resources Unit, 630 W. North Street, Geneva, NY, 14456, USA

**Keywords:** Cryptic introgression, Linkage drag, Breeding, DNA sequence, *Solanum* species

## Abstract

**Background:**

Many highly beneficial traits (e.g. disease or abiotic stress resistance) have been transferred into crops through crosses with their wild relatives. The 13 recognized species of tomato (*Solanum* section *Lycopersicon*) are closely related to each other and wild species genes have been extensively used for improvement of the crop, *Solanum lycopersicum* L. In addition, the lack of geographical barriers has permitted natural hybridization between *S. lycopersicum* and its closest wild relative *Solanum pimpinellifolium* in Ecuador, Peru and northern Chile. In order to better understand patterns of *S. lycopersicum* diversity, we sequenced 47 markers ranging in length from 130 to 1200 bp (total of 24 kb) in genotypes of *S. lycopersicum* and wild tomato species *S. pimpinellifolium*, *Solanum arcanum*, *Solanum peruvianum*, *Solanum pennellii* and *Solanum habrochaites*. Between six and twelve genotypes were comparatively analyzed per marker. Several of the markers had previously been hypothesized as carrying wild species alleles within *S. lycopersicum*, i.e., cryptic introgressions.

**Results:**

Each marker was mapped with high confidence (e<1 x 10^-30^) to a single genomic location using BLASTN against tomato whole genome shotgun chromosomes (SL2.40) database. Neighbor-joining trees showed high mean bootstrap support (86.8 ± 2.34%) for distinguishing red-fruited from green-fruited taxa for 38 of the markers. Hybridization and parsimony splits networks, genomic map positions of markers relative to documented introgressions, and historical origins of accessions were used to interpret evolutionary patterns at nine markers with putatively introgressed alleles.

**Conclusion:**

Of the 47 genetic markers surveyed in this study, four were involved in linkage drag on chromosome 9 during introgression breeding, while alleles at five markers apparently originated from natural hybridization with *S. pimpinellifolium* and were associated with primitive genotypes of *S. lycopersicum*. The positive identification of introgressed genes within crop species such as *S. lycopersicum* will help inform conservation and utilization of crop germplasm diversity, for example, facilitating the purging of undesirable linkage drag or the exploitation of novel, favorable alleles.

## Background

Introgression is the transfer of genes of one species into the gene pool of another via hybridization. As a phenomenon, it has been an important topic in animal and plant genetics research for many different reasons. For example, introgression has been implicated in the adaptation of modern humans [1] and is of concern to conservation biologists due to loss of integrity of wild bird and mammal populations [2,3]. In plants, introgression is a key concept in studies of the risks of contamination of natural populations by genetically modified (GM) crops. More commonly in crops, favorable genes from wild relatives are intentionally transferred into breeding lines for cultivar development. This has been particularly valuable in crop species that are relatively low in genetic diversity.

According to a review of crop introgression breeding [4] the major functional categories of beneficial traits transferred from wild species are resistance or tolerance to abiotic stress or disease, yield, cytoplasmic male sterility or fertility restorers for hybrid production, and quality traits. Among the pioneering uses of wild crop relatives during the late 19^th^ to early 20^th^ centuries were the transfer of disease resistances into grape (*Vitis vinifera*) [5] and sugarcane (*Saccarum officinarum*) [6]. A 1986 review of 23 crops estimated that 6% of total annual economic value in the US was contributed by crop wild relatives [7]. For 13 major crops of global importance, it was estimated that 46 wild species have been used in released cultivars, and that furthermore, the introgression breeding approach is increasing [4]. Lack of information on pedigrees, unpublished activities within the private sector and changes in taxonomy are some of the factors that contribute to the uncertainty of the collective impacts of crop introgression breeding [4].

Some of the earliest tomato introgression breeding in the US may have been done indirectly and unwittingly via the French variety Merville des Marchés. Recent phenotypic data collected for Merville des Marchés PI 109834 showed it to be variable in fruit size and smoothness ( http://www.ars-grin.gov/cgi-bin/npgs/acc/display.pl?1129442); its genotype was segregating, showed population admixture, and was an outlier based on genetic distance relative to many other *S. lycopersicum* accessions [8,9]. We postulated that these were indications of *S. pimpinellifolium* in its ancestry (this idea was examined in the current study). The *Fusarium* wilt-resistant processing variety Marvel [10] was selected from Merville des Marchés in the early 1900s, and Marvel was a parent of Marglobe released in 1925 [11], which in turn can be found in the pedigree of many important varieties from the 1930s through the late 1950s (H.M. Munger’s tomato pedigree chart provided by E.D. Cobb, Cornell University, 2012). Direct introgression of tomato with wild species in the US commenced in the 1930s concurrent with collection expeditions to geographic centers of origin. The first released cultivar, developed from Marglobe x *S. pimpinellifolium*, was aptly named Pan American [12]. Introgression breeding efforts of tomato increased globally post World War II, involving the screening of a wide range of traits and all wild tomato species [13]. Such efforts continue to be of utmost priority today using sophisticated tools such as introgression libraries for gene discovery [14,15].

Compellingly, of 96 introgressed traits tallied in released crop cultivars for 11 species (cassava, wheat, millet, rice, maize, sunflower, lettuce, banana, potato, groundnut, tomato), 55 of them were in tomato (*Solanum lycopersicum* L.); the next highest numbers were found in rice and potato with 12 traits each [4]. The emphasis and success of introgression breeding in tomato encompasses several factors including its intrinsically narrow genetic base, relative ease of crossing with several wild taxa, production demands based on growing conditions and market niche, its susceptibility to pests and pathogens, and its sensitivity to abiotic factors. In addition to resistance or tolerance to dozens of bacterial, viral, fungal, insect, and nematode pathogens, hundreds of favorable genes or quantitative trait loci (QTL) for abiotic stress resistance, flower and fruit traits, yield, and plant architecture have been mapped in wild tomato species [16] and thus hold the potential to be exploited.

Introgression breeding carries a cost, namely, genetic linkage of non-targeted loci that are eliminated through repeated backcrossing. Linkage drag can persist within a genome despite backcrossing, especially if recombination is suppressed. Several examples of linkage drag in tomato and other crops have been quantified using molecular markers [17-21]. Linkage drag can denote favorable, deleterious or neutral alleles that become inadvertently incorporated into breeding lines or cultivars.

In this study we apply the term ‘cryptic introgression’ [22] to describe latent genetic variation in *S. lycopersicum* that originated from wild tomato species. Various scenarios can be evoked for its origins ranging from linkage drag, hybridization between feral *S. lycopersicum* and wild relatives, to crossing in open-pollinated populations by wind or insect vectors with pollen of introgressed cultivars [23]. Cryptic introgression is of interest in germplasm collections such as those conserved at United States Department of Agriculture, Agricultural Research Service (USDA, ARS) Plant Genetic Resources Unit (PGRU) because it can indicate novel genetic variation for exploitation by end-users, or conversely, reveal unfavorable and hence undesirable alleles with respect to crop improvement.

In previous reports we hypothesized the detection of cryptic introgression in 5% to 10% of DNA markers that were resequenced in tomato germplasm panels [9,24,25]. The aim of the current study was to gather additional evidence on these alleles by resequencing and analyzing the same markers in several accessions of wild tomato species and one accession of weedy *S. lycopersicum* (Table 1). Although variation within wild species gene pools made it impracticable to attempt to discover the 100% identical homologous allele, the assumption was that introgressed alleles would be more closely related to the alleles of a particular wild species than to their *S. lycopersicum* homologs. To identify introgressed alleles we also used evidence from mapped locations of markers, phenotypic descriptions, and historical origins of lines and accessions.

**Table 1:** Tomato samples analyzed in this study

**Species**	**Accession or line**	**Description**^**a**^
*Solanum habrochaites*	PI 126445	collected from Peru in 1937, source of *Cyc*-*B*, green-fruited
*Solanum pennellii*	PI 414773	collected from Peru in 1976, source of *I*-*1*, *I*-*3*, green-fruited
*Solanum peruvianum*	G 32592 (LA4125)	naturally selfing, collected from Chile in 2001, green-fruited
*Solanum peruvianum*	LA1537	artificially inbred from PI 128650 collected from Chile in 1938 and source of *Tm-2*^*a*^, green-fruited
*Solanum arcanum*^b^	G 32591 (LA2157)	naturally selfing, collected from Peru in 1980, source of *Cm* QTLs, green-fruited
*Solanum pimpinellifolium*	PI 370093	traces back to Vaughan Seed Co., Chicago, USA, circa 1930, source of *Cf-2*, *Cf-3*, *Pto*, red-fruited
*Solanum lycopersicum*	PI 303801	Peru Wild (syn. with Utah 665) from Utah Agr. Expt. Sta., source of *Ve*, red-fruited
*Solanum lycopersicum*^b^	PI 99782	Tomate, collected in 1932 from Peru, red-fruited
*Solanum lycopersicum*^b^	PI 109834	Merville des Marchés, collected in 1935 from France, red-fruited
*Solanum lycopersicum*^b^	PI 129026	unnamed, collected in 1938 from Ecuador, red-fruited
*Solanum lycopersicum*^b^	PI 129128	unnamed, collected in 1938 from Panama, red-fruited
*Solanum lycopersicum*^b^	PI 196297	unnamed, collected in 1951 from Nicaragua, red-fruited
*Solanum lycopersicum*^b^	PI 258474	unnamed, collected in 1959 from Ecuador, red-fruited
*Solanum lycopersicum*^b^	PI 258478	unnamed, collected in 1959 from Peru, red-fruited
*Solanum lycopersicum*^b^	PI 390510	unnamed, collected in 1974 from Ecuador, red-fruited
*Solanum lycopersicum*^b^	TA496	from S. Tanksley, Cornell Univ., developed in 1990s from E6203 x Vendor-*Tm-2*^*a*^, red-fruited

Germplasm sources of sequenced alleles in wild and cultivated tomato accessions.

^a^ For complete references tracing history of these germplasm sources used for introgression breeding see [26].

^b^ Sequences published in [25].

## Results and discussion

### Markers and *in silico* mapping

For the 47 markers used in this study (Additional file 1: Table S1), nucleotide primers for PCR and sequencing were originally designed against *S. lycopersicum* sequences except for the Conserved Ortholog Set II (COSII or C2) and unigene (U) markers, which were designed against Euasterids [27,28]. Molecular markers have shown good success rates of transferability among distantly related wild tomato species such as *S. lycopersicum* and *S. pennellii* (for examples see [29-31]). In the current study, some primer pairs did not amplify or give clean or homologous reads in every wild tomato sample. The COSII and U markers did not outperform the other markers in terms of successfully generating high quality sequence data because most of them contained introns, which sometimes carried small indels in a heterozyogus condition. Such heterozygous indels were major contributors to poor quality reads and hence missing data.

In our current germplasm panel (Table 1), *S. lycopersicum* and *S. pimpinellifolium* had no missing markers; sequences were available for at least two of the four green-fruited taxa for the final set of 47 markers. *S. peruvianum* LA1537, *S. peruvianum* G32592, *S. arcanum*, *S. pennellii* and *S. habrochaites* gave data for 32, 42, 40, 35 and 38 markers, respectively. The latter two species are usually self-incompatible (SI) and carried greater numbers of polymorphic markers within accessions than the inbred accessions of *S. peruvianum* and *S. arcanum* that were sampled. Mean SNP frequency across the polymorphic markers was 0.0127 (*n* = 17) for *S. habrochaites*, 0.0082 (*n* = 17) for *S. pennellii*, 0.0126 (*n* = 5) for *S. peruvianum* LA1537, 0.0058 (*n* = 2) for *S. peruvianum* G32592 and 0.0078 (*n* = 7) for Peru Wild. TA496, Tomate and *S. arcanum* had no polymorphic sites in any of the markers.

*S. pimpinellifolium* showed unusually high polymorphism of 0.0055 (*n* = 17) relative to the other self-compatible taxa. As one explanation, this accession was categorized as an admixture population in a simple sequence repeat (SSR) genotyping study of *S. pimpinellifolium* population structure [32], so naturally represents two dissimilar *S. pimpinellifolium* genomes. Because the seed source traces back to Vaughan Seed Co. in USA and Horticultural Experiment Station, Ontario, Canada [33], another possibility is that *S. lycopersicum* was incorporated into its pedigree by one of those entities. This may be evidenced by comparing the previously estimated *D*, number of mutations per kb, [34] between the two species [35] which was approximately four-fold greater than our estimate reported below.

**Figure 1:**
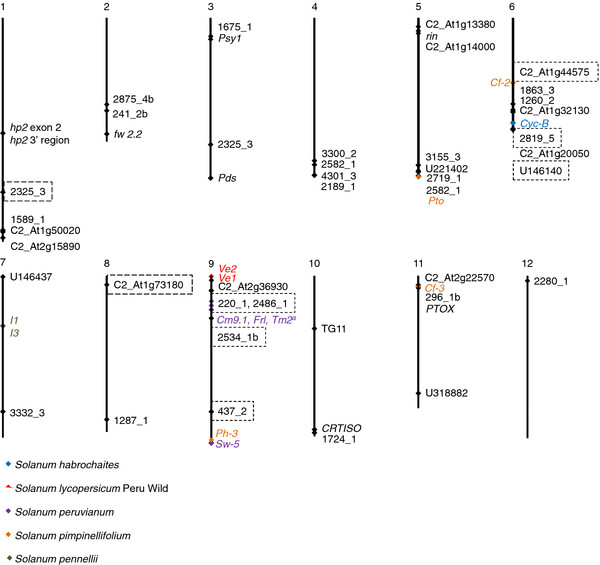
**Chromosomal map locations of 47 markers sequenced in this study.** Nine markers with dashed outlines showed cryptic introgressions. Also shown on the map (color) are documented introgressions used in tomato breeding that were mentioned in this report, *S. habrochaites*: blue, *S, lycopersicum* Peru Wild: red, *S. peruvianum*: purple, *S. pimpinellifolium*: orange, *S. pennellii*: green.

The majority of markers were mapped with high confidence to a single map location within the genome (Figure 1, Additional file 1: Table S1). This is the first report in which a whole-genome sequence [36] and web based tools were available with which to do this for the expressed sequence tag (EST) -based markers [24]. Accordingly, 12 of the EST-based markers were newly mapped and markers 437_2, 2189_1 and 2819_5 were revised with respect to previously predicted chromosomal location based on identities to restriction fragment length polymorphism (RFLP) markers [25]. All chromosomes were represented by the 47 markers; numbers of markers per chromosome ranged from one on chromosome 12 to eight on chromosome 6. Small gaps were observed in the alignments but close examination revealed that these were in masked regions rich in runs of poly A or poly T. Only marker 1675_1 did not initially provide any hits. A lowered stringency of 1 x 10^-30^ subsequently found the probable position.

Four markers had two BLASTN hits each. These were 1260_2 for two tightly linked (2,849 nt apart) sequences on chromosome 6, 2325_3 on chromosomes 1 and 3, 2582_1 on chromosomes 4 and 5, and 2819_5 for two linked (14,114 nt apart) regions on chromosome 6. A check of the primer regions found multiple mismatches in predicted primer binding sites of the secondary hit for three of the markers; these were assumed to have amplified as single-copy. For marker 2819_5 the forward primer had a single mismatch and the reverse had no mismatches. The predicted amplicon in the mismatch region was 86% identical to the reference TA496 sequence. When the mismatch sequence was included in MEGA cluster analysis, it separated from all other (wild and cultivated) alleles with 87% bootstrap support, and the mean *D* among sequences increased 8-fold (*D* = 1.5 for *n* = 10 alleles versus *D* = 12 for *n* = 11 alleles). Therefore, it is unlikely that this paralog amplified and confounded the results. All markers had previously been screened in our lab for amplification of single bands and highly homozygous sequences within *S. lycopersicum*[24,25]. Results of *in silico* mapping confirmed that this set of markers provides robust results in sampling single-copy *S. lycopersicum* genes. All sequences were deposited into the European Molecular Biology Laboratory (EMBL), European Nucleotide Archive (ENA) data base as accession numbers HE977919-HE978211.

### Clustering patterns and divergence estimates among taxa

PI 99782 Tomate was chosen for the current study to represent a ‘pre-introgression breeding’ genotype. It bears small, slightly ribbed and unimproved fruit with scarring and cracking ( http://www.ars-grin.gov/cgi-bin/npgs/acc/display.pl?1127604) and was homozygous for the common *S. lycopersicum* haplotype at 48 of 50 markers for which it had been sequenced [25]. For 38 of the markers there was high bootstrap support for the red-fruited clade that consisted of *S. lycopersicum* including Tomate and *S. pimpinellifolium* alleles (Additional file 1: Table S2). Bootstrap values ranged from 54% to 100% (mean ± standard error = 86.8 ± 2.34%). These bootstrap values subtracted from 100% can be interpreted as estimates of the probability of Type I error, i.e., falsely accepting a cluster that is not signified by the data (see [37] for discussion).

Results underscored the close relationship of *S. lycopersicum* to *S. pimpinellifolium* because their alleles were frequently identical or highly similar. The placement of green-fruited species with respect to the red-fruited clade and each other varied among loci. This can result from incomplete lineage sorting or introgression [38]. An example was the placement of *S. habrochaites* near *S. pimpinellifolium* for marker TG11 (Additional file 2: Figure S1); this example was also reported by Nesbitt and Tanksley [35].

Of the nine markers that did not support the red-fruited clade, seven had previously been hypothesized as carrying introgressions (Additional file 1: Table S2). These are discussed below. The other two markers, 1287_1 and 2280_1, showed patterns that contained *S. peruvianum* within the red-fruited clade (Additional file 2: Figure S1) seemingly due to lack of resolution. At marker 1287_1 *S. peruvianum* haplotypes were only one or two mutational steps from Tomate. These mutations were not shared with any other taxa. At marker 2280_1 only four unique haplotypes were observed. These were *S. habrochaitesS. pennelliiS. arcanum* and {TA496, Tomate, *S. pimpinellifolium*, Peru Wild, G32592, LA1537}. For the 38 markers that supported the red-fruited clade, accepted taxonomic relationships among tomato species were generally supported [39,40].

Divergence estimates (*D*) among loci are associated with a high variance over evolutionary time due to differences in mutation and recombination rates, selective constraints, and influences of various factors such as random sampling of gametes and demography. Average *D* ranged from 5 for marker 4301_3 to 52 mutations per kb for marker C2_At1g44575 (mean ± standard error = 16.5 ± 1.29). At the aggregate scale the mean *D* ± standard error from PI 99782 Tomate was as follows: TA496 *D* = 0.001 ± 0.0005, Peru Wild *D* = 0.002 ± 0.0006, *S. pimpinellifolium D* = 0.002 ± 0.0004, *S. arcanum D* = 0.020 ± 0.0022, *S. peruvianum* LA1537 *D* = 0.020 ± 0.0025, *S. peruvianum* G32592 *D* = 0.022 ± 0.0024, *S. habrochaites D* = 0.021 ± 0.0024 and *S. pennellii D* = 0.022 ± 0.033.

The lack of precise resolution in distinguishing taxa by clustering, or by average divergence from Tomate in the case of the green-fruited taxa was a function of shared polymorphisms in many instances, e.g., at 24 markers at least one single nucleotide polymorphism (SNP) within a species was also segregating between other species pairs. This has been observed in other studies of crop species and their closely related wild relatives ( [41] and references therein). In addition, random sampling contributed to low resolution, e.g., *S. peruvianum* haplotypes appeared to be derived from Tomate at marker 1287_1 based on a small number of noninformative SNPs.

### Evidence for introgression

In previous studies we reported nine markers (Table 2) with highly diverged alleles within *S. lycopersicum* and hypothesized that this was due to introgression from wild species. These were 220_1, 437_2, 2325_3, 2534_1R (redesigned into 2534_1b), 2486_1 [24], 2819_5, C2_At1g73180 [25], U146140 and C2_At1g44575 [9]. Of the 47 markers in the current study, seven of these nine showed patterns in cladograms that did not cluster together members of the red-fruited clade (Additional file 2: Figure S1). Hybridization networks (Additional file 2: Figure S1), descriptive information of the accessions, map positions of markers, and species origins of documented introgressed disease resistance alleles (e.g. [16,26,42-44]) were used as total evidence to categorize the divergent markers into putative linkage drag during introgression breeding versus natural out crossing with *S. pimpinellifolium* (Table 2). This categorization did not constitute proof of natural hybridization versus introgression breeding. However, it was a useful concept from which to synthesize independent lines of evidence and can serve as a basis for future hypothesis testing of the two scenarios.

**Table 2:** Tomato markers tested for cryptic introgression

**Marker**	**SGN gene model, predicted protein**	**Chromosome location (MB)**	**Divergent line or accession**	**Genetic-distance based clustering results**^a^	**Observations**
Introgression from crop improvement					
220_1	Solyc09g014280, hydroxycinnamoyl transferase	ch09, 5.77	TA496	TA496 intermediate between LA1537 and *S. pennelli*	Major disease resistance genes on ch09 include:
					*Ve2*, 0.05 MB, (11.002 cM^b^), from Peru Wild
2486_1	Solyc09g014350, glycerol-3-phosphate acyltransferase 6	ch09, 5.90	TA496	low bootstrap values overall except {TA496, LA1537}	
					*Ve1*, 0.06 MB, (11.002 cM), from Peru Wild
2534_1b	Solyc09g018790, succinic semialdehyde reductase isofom1	ch09, 17.00	TA496	TA496 clustered with two *S. peruvianum* accessions	*Cm9.1*, (4.0 – 24.0 cM), from *S. peruvianum*
					*Frl*, (27.0 – 37.0 cM), from *S. peruvianum*
437_2	Solyc09g061440, uncharacterized protein	ch09, 54.71	TA496	TA496 intermediate between {*S. peruvianum*, *S. arcanum*, Peru Wild, *S. pimpinellifolium*, Tomate} and {*S. pennellii*, *S. habrochaites*}	
					*Tm2*^*a*^, 13.62 MB, (32.002 cM), from *S. peruvianum*
					*Sw-5*, 67.30 MB, (78.001 cM), from *S. peruvianum*
					*Ph-3*, 66.71 – 66.78 MB, (63.0 – 78.0 cM), from *S. pimpinellifolium*
Introgression from natural hybridization with *S. pimpinellifolium*					
2325_3^c^	Solyc01g073640, alcohol dehydrogenase-3	ch01, 70.26	TA496	red-fruited clade was supported	PI 258478 was collected from Peru in 1959, highly variable, fasciated fruit.
			PI 258478		
C2_At1g44575	Solyc06g060340, chloroplast photosystem II-associated protein	ch06, 34.71	PI 258478	red-fruited clade was supported	Introgressions on ch06 include:
					*Cyc-B*, 42.29 MB, (106 cM), from *S. habrochaites*
2819_5^c^	Solyc06g082670, ribosomal protein L10	ch06, 44.70	PI 258478	red-fruited clade was split into {Peru Wild-1, Tomate, TA496} and {Peru Wild-2, *S. pimpinellifolium*, PI 258478}	
U146140	Solyc06g083360, DNA-directed RNA polymerase II subunit	ch06, 45.08	PI 109834	red-fruited clade was split into {PI 109834, *S. pimpinellifolium*} and {TA496, Peru Wild, Tomate}	PI 109834 Merville des Marchés was collected from France in 1935.
C2_At1g73180	Solyc08g014060, eukaryotic translation initiation factor 3 subunit 9-like protein	ch08, 3.57	PI 129026	{PI 196297, PI 390510} were divergent from other members of red-fruited clade	PI 196297 was collected in Nicaragua in 1951, fasciated fruit, reported as introgressed by Rick [23]; carries same allele as PI 129026 (from Ecuador, 1938, fasciated fruit), PI 129128 (from Panama, 1938, fasciated fruit), PI 258474 (from Ecuador, 1959, fasciated fruit). PI 390510 was collected in Ecuador in 1974, described as a wild cherry tomato.
			PI 129128		
			PI 196297		
			PI 258474		
			PI 390510		

Nine tomato markers previously identified as carrying highly divergent alleles within *Solanum lycopersicum*.

^a^ (Additional file 2: Figure S1).

^b^ Genetic linkage map positions from [45] or [46].

^c^ Sequence mapped to two locations, see Results and discussion.

Linkage drag was inferred for at least three of the four markers at which TA496 carried an allele that was highly divergent from all other members of the red-fruited clade. All four mapped to chromosome 9 and spanned from 5.77 MB to 54.71 MB (Table 2). Introgressed disease resistance loci documented on chromosome 9 [42] include *Ve1* (0.06 MB) and *Ve2* (0.05 MB) both from Peru Wild, *Frl* (physical map position not annotated), *Tm-2*^*a*^ (13.62 MB) and *Sw-5* (67.30 MB), all three from *S. peruvianum*, and *Ph-3* (66.71-66.78 MB) from *S. pimpinellifolium* (Figure 1, Table 2). LA1537 was the most closely related allele to TA496 in cladograms for the three markers spanning 5.77 – 17.00 MB on chromosome 9, which encompasses the *Tm-2*^*a*^ locus at position 13.62 MB. LA1537 is an inbred accession that was derived from PI 128650, the original source of *Tm-2*^*a*^ (Table 1).

**Figure 2:**
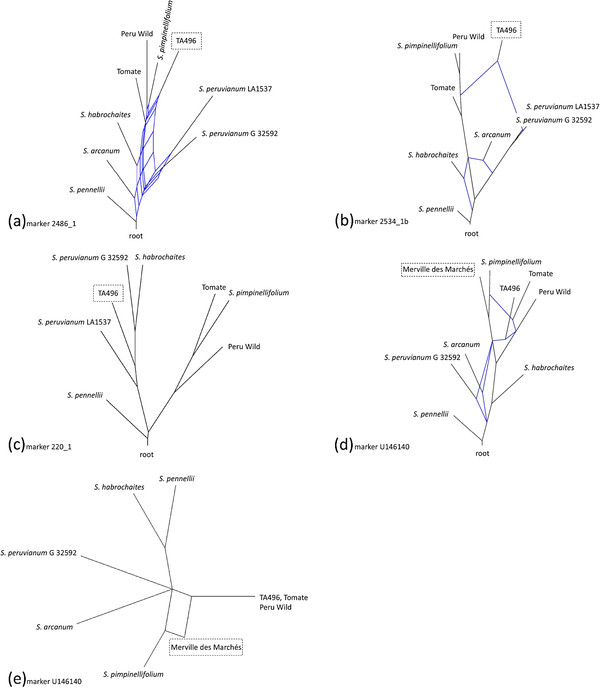
**Examples of splits networks at markers with cryptic introgressions in*S. lycopersicum.*** Dashed boxes indicate accessions with introgressed alleles (a-d: hybridization networks using marker U146437 as a control, e: parsimony splits network); **a**) TA496 showed reticulation with *S. peruvianum* LA1537 and other green-fruited species, **b**) TA496 showed reticulation with red-fruited and green-fruited species, **c**) TA496 nested within green-fruited species, **d**) Merville des Marchés PI 109834 was closely related to *S. pimpinellifolium*, which showed reticulation to Tomate PI 99782, **e**) parsimony splits network used only conservative, global signal within the marker to illustrate the hybrid origin of Merville des Marchés PI 109834.

Hybridization networks placed TA496 in various positions for each of the three markers surrounding *Tm-2*^*a*^: near the red-fruited alleles with reticulation back towards *S. peruvianum* for marker 2486_1 (Figure 2a and Additional file 2: Figure S1), between the red-fruited alleles and *S. peruvianum* with reticulation back towards both for marker 2534_1b (Figure 2b and Additional file 2: Figure S1), and within the green-fruited wild species alleles between the two *S. peruvianum* accessions, with no reticulation for marker 220_1 (Figure 2c and Additional file 2: Figure S1). The size of the *Tm-2*^*a*^ introgressed region was estimated by mapping in segregating F_2_ populations [19]. RFLP marker TG101 at chromosome 9 location 50.41 MB was tightly linked (≤ 1 cM) to *Tm-2*^*a*^. The chromosomal position of *Tm-2*^*a*^ has been characterized as very near the centromere with extremely repressed recombination [47]. It is therefore probable that markers 220_1 (5.77 MB), 2486_1 (5.90 MB) and 2534_1b (17.00 MB) were part of the introgressed segment in TA496.

The TA496 allele at marker 437_2 (54.71 MB) was not as definitively related to *S. peruvianum* and its origin was more difficult to interpret. The cladogram placed TA496 in an intermediate position between two clades, namely, {*S. pimpinellifolium*, Tomate, *S. arcanum*, Peru Wild, *S. peruvianum*} versus {*S. habrochaitesS. pennellii*} (Additional file 2: Figure S1). The hybridization network showed reticulation with *S. habrochaites* and the red-fruited alleles (Additional file 2: Figure S1). Several genes commonly found in tomato varieties have originated from *S. habrochaites* (*Cf-4Tm-1Ol-1Cyc-B* and *Del*) or *S. pennellii* (*I1* and *I3*) but none of these map to chromosome 9 [43,45]. The chromosome 9 introgressions listed in Table 2 were judged to be commonly used in cultivars based on an informal survey of descriptions from online seed catalogs (unpublished observations) as well as comprehensive reviews of tomato breeding (e.g. [16,26,42-44]).

Linkage drag of 437_2 in TA496 with *Ph-3* (66.71 MB) or *Sw-5* (67.30) was rejected because they originated from *S. pimpinellifolium* and *S. peruvianum*, respectively. Based on a BLASTN search of TA496 marker 437_2 against the Solanaceae PlantGDB-assembled Unique Transcipts (PUTs) in the Solanaceae Genomics Resource database ( http://solanaceae.plantbiology.msu.edu/), TA496 and *S. habrochaites* shared a sympleisiomorphy at nucleotide position 46 that was absent from all other alleles that we resequenced. This provided tentative but inconclusive evidence of a direct relationship between the introgression and *S. habrochaites*. As alternative evidence, FM6203, a progenitor of TA496, purportedly carries *Asc* resistance (pers comm. S. Loewen, University of Guelph, 2005) for which *S. pennellii* has served as one of the original sources on chromosome 3 in tomato [48].

**Figure 3:**
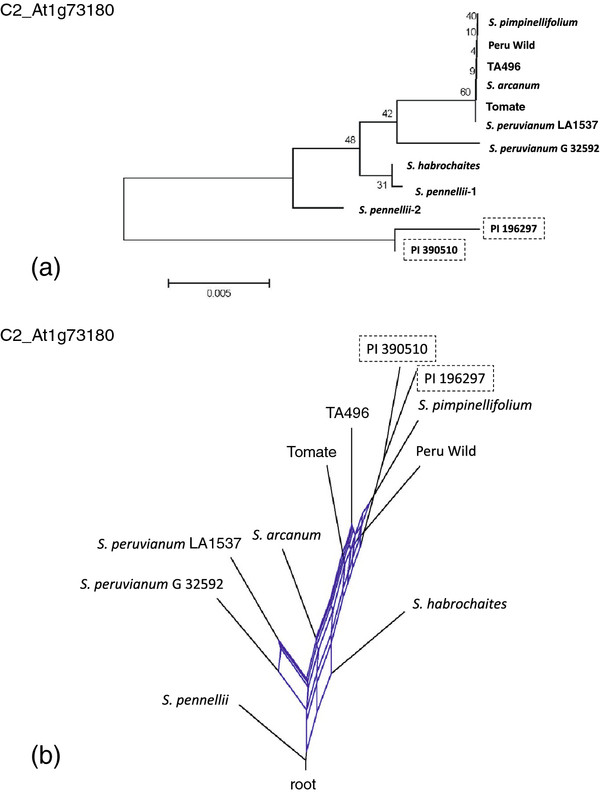
**Extreme divergence of marker C2_At1g73180.a**) Clustering at marker C2_At1g73180 showed the distinctiveness of PI 129026 and PI 196297 with 99% bootstrap support, **b**) the splits network showed a combination of introgression of PI 129026 and PI 196297 (among others, see Table 2) with *S. pimpinellifolium* and retention of ancient polymorphisms that reticulated down to the root; the latter supported genetic hitchhiking and rejection of selective neutrality.

The remaining five markers with divergent alleles showed patterns consistent with introgression from *S. pimpinellifolium* in natural populations. At four markers (2325_3, C2_At1g44575, 2819_5 and U146140) the divergent alleles were more closely related to red-fruited rather than green-fruited taxa with bootstrap values ranging from 82% – 100% (Additional file 1: Table S2), although markers 2819_5 and U146140 did not support monophyly of red-fruited alleles (Additional file 2: Figure S1). Hybridization networks showed red-fruited species alleles to be distinct from green-fruited species alleles for 2325_3 and C2_At1g44575, while 2819_5 and U146140 showed connections between red-fruited and green-fruited species (Additional file 2: Figure S1). Marker U146140 nicely illustrated the *S. lycopersicum* x *S. pimpinellifolium* hybrid origin of Merville des Marchés PI 109834 (Figures 2d 2e). Marker C2_At1g73180 showed an unusual pattern in that PI 196297 (and three additional accessions with the identical allele, Table 2) and PI 390510 were divergent from all other alleles in the cluster analysis with 99% bootstrap support (Figure 3a and Additional file 2: Figure S1). This suggested a potential paralog. However, no heterozygotes were observed, the marker did not map to more than one genomic location, and COSII markers were designed to amplify highly conserved single copy genes [27].

The divergent C2_At1g73180 alleles were unlikely to have originated from a green-fruited species because chromosome 8 does not carry any introgressed disease resistance alleles [16,42-44]. The hybridization network depicted PI 196297 and PI 390510 as branching from *S. pimpinellifolium*, with complex reticulations at the base of the red-fruited clade that extended through the green-fruited taxa, down to *S. pennellii* at the root (Figure 3b). Among the seven unique polymorphisms carried by PI 196297 and PI 390510, two were non-conservative amino acid substitutions, two were synonymous and three were intronic. One possibility is that selection at or near this locus has caused ancient polymorphism to have been retained. A significant HKA test [49] (*χ*^2^ = 7.36, *P* = 0.007) strengthened this interpretation. Therefore, diversity at this marker showed patterns of both natural selection and introgression.

Additional evidence that these five markers represent *S. pimpinellifolium* introgressions in natural populations includes geographical origins of three accessions from Ecuador where the two species hybridize extensively [50], original collection of three accessions dating to 1935 and 1938 (likely precluding the influence of direct introgression breeding), the primitive fruit phenotype of fasciation (four accessions) or wild cherry tomato (one accession, n.b., cherry tomato was described as an admixture of *S. lycopersicum* and *S. pimpinellifolium* by [35]), and previously reported introgression of PI 196297 with *S. pimpinellifolium* by Rick [23]. In estimates of population structure [51] PI 109834, PI 129128, PI 258474 and PI 258478 all showed high probability of membership in the second of two populations inferred for *S. lycopersicum* genotypes, consistent with interspecific hybridization [9].

## Conclusions

It was useful to delineate cryptic introgression within *S. lycopersicum* into linkage drag stemming from breeding versus natural hybridization with *S. pimpinellifolium*, although this categorization was not definitive and should be subjected to further scrutiny. Sequences of the wild tomato species markers in the context of their physical map locations have strengthened our previous interpretations of detection of introgressed alleles in domesticated tomato [24,25]. Genomic tools for fine resolution of introgressed regions in crop species are increasingly available. The strengths and weaknesses of comparative genotyping to verify introgression have been illustrated here using *Solanum* section *Lycopersicon* taxa.

In the current study the implications of linkage drag of introgressed alleles on phenotype remain unknown. At least one marker (2534_1b) codes for an enzyme involved in fruit ripening (Table 2). In cultivars or breeding lines it will be more useful to estimate the proportion of a genome that harbors linkage drag. This should be feasible for TA496 using bioinformatics given the vast amount of public EST sequence data available for this particular line and wild tomato species ( http://solgenomics.net/tools/blast/dbinfo.pl as of June 2012 reported 323,465 Lycopersicon mRNAs). Importantly, an understanding of linkage drag will help to distinguish it from selection during crop improvement. Four of six markers that previously rejected neutrality tests (437_2, 2486_1, 2534_1b and 2819_5) (Table 2 in [25]) were found to be introgressed rather than selected. It is anticipated that next-generation sequencing will be utilized to more rapidly eliminate linkage drag in crops [52].

Natural hybrids will carry high proportions of wild alleles making them somewhat easy to detect using large numbers of molecular markers. The intrinsic value of naturally introgressed germplasm was recognized in common bean (*Phaseolus vulgaris*) as a source of new alleles for traits such as disease resistance [53]. In *S. lycopersicum*, if horticultural effects of introgression are subtle then accessions such as Merville des Marchés may be prime sources to screen for new alleles. A search of the literature for accessions that were part of the current study (Table 2) found that PI 129128 showed high lycopene content, similar to lines containing pigment mutations such as *og*^*c*^*, hp, and dg*[54].

Finally, it is worth noting that Rick [23] reported the potential of natural hybridization of *S. lycopersicum* with *Solanum chilenseS. habrochaites* (formerly *Lycopersicon hirsutum* f. *glabratum*), and *Lycopersicon peruvianum* (now revised into four species, [55]) in regions of Chile, Ecuador and Peru where sympatric populations grow in close contact, although he found no evidence of this based on phenotypes and severe postzygotic barriers are well known. Genotyping of populations sampled from these regions would provide evidence to reexamine whether introgression from these wild tomato species into *S. lycopersicum* has played a role in the crop’s evolutionary history.

## Methods

### Plant material

Marker genotypes used in this study were previously reported for *Solanum arcanum* and *S. lycopersicum*[9,25] or were newly collected from each of five wild tomato accessions and one weedy *S. lycopersicum* accession Peru Wild. The specific accessions of *S. habrochaitesS. pennelliiS. pimpinellifolium* and Peru Wild have served historically as sources of important disease resistance alleles for tomato cultivars (Table 1). The two *S. peruvianum* accessions were chosen because they were known to be inbred and predicted to be highly homozygous. One of these, *S. peruvianum* LA1537, originated from accession PI 128650 which was the original source of *Tm-2*^*a*^[56,57]. Two plants per each of the six accessions were sampled as seedlings for genomic DNA isolation and sequencing of markers. Three accessions included in this study had previously published sequences for the markers [25] (Table 1). These were − a breeding line with documented multiple introgressions in its pedigree including *Tm-2*^*a*^ (TA496) [57], an accession that predated tomato introgression breeding (Tomate, PI 99782), and *S. arcanum* (G 32591) a naturally self-fertilizing accession formerly classified as *Lycopersicon peruvianum*. For a few markers, published sequences from additional *S. lycopersicum* accessions (PI 109834 Merville des Marchés, PI 129026, PI 129128, PI 196297, PI 258474, PI 258478, PI 390510, Table 1) were included in analyses because they were previously reported as carrying highly divergent alleles, i.e. putative introgressions, at those loci [9,25].

### DNA sequences

Genomic DNA extraction from seedlings, PCR amplification and two-pass sequencing were as described in Labate et al. [28]. Initially, 49 of 50 markers from Labate et al. [9] were sequenced. These represent random loci including expressed genes (expressed sequence tag, EST-based), highly conserved genes (COSII and U) and arbitrary loci. Marker 1523_4 was excluded without testing because it tended to give poor quality sequence within *S. lycopersicum*. Markers 175_1 and 1909_2 were dropped during this study because they did not consistently amplify *S. lycopersicum* homologs in wild tomato species, leaving 47 markers representing approximately 24 kb in total (Additional file 1: Table S1). Software packages phred, phrap and Consed [58,59] and Staden [60] were used for assembly and base calling of reads. Pregap4 (ver. 1.5) of the Staden package was configured to apply a base-calling algorithm “Estimate Base Accuracies“ that is different from phred in order to independently verify the data. Sequence data were trimmed to remove primer binding sites and low quality ends (phred<40), and manually aligned in BioEdit [61]. All SNPs and heterozygous positions were confirmed by visual examination of trace files by two people. If the two plants from one accession had different sequences they were kept distinct; if they were identical they were treated as a single representative sequence of that accession. Heterozygous sites were manually edited to use IUPAC nucleotide ambiguity codes. GeneSeqer (ver. 08 Oct. 2008) [62] was used to compare exon and intron prediction for all markers against previous annotations [28].

All sequences were mapped using BLASTN [63] against tomato whole genome shotgun chromosomes (SL2.40) database with an e-value threshold of 1 x 10^-40^ on the SGN web site [64]. Gene models within markers and adjacent regions were identified in the SGN genome browser using ITAG2.3 Release: genomic annotations. For marker 437_2, tomato sequences were compared to transcribed sequences from an evolutionary out group (potato, *Solanum tuberosum*) by BLASTN searches of the Solanaceae Genomics Resource database at Michigan State University ( http://solanaceae.plantbiology.msu.edu/).

### Statistical analyses

For each of the 47 markers, relationships among genotypes (also referred to as taxa) were first examined by applying the neighbor-joining (NJ) clustering method [65] as implemented in MEGA 4.0.2 [66] with 1,000 bootstrap replicates. Genetic distance and average evolutionary divergence (*D*) were estimated using the Jukes-Cantor method [34]; positions with alignment gaps or missing data were eliminated in pairwise sequence comparisons. For each of 11 markers that did not support the red-fruited clade based on MEGA results, consensus trees were generated by Phylip ver. 3.69 using Seqboot to produce 100 datasets by bootstrap resampling, Dnadist to estimate genetic distances using the Jukes-Cantor method, Neighbor to produce unrooted NJ trees and Consense to compute a consensus tree by the majority-rule consensus tree method [67]. SplitsTree4 ver. 4.12.3 [68] was used to create splits networks from DNA sequences or NJ trees [69]. Hybridization splits networks were created using the consensus tree of marker U146437 as a control that highly supported the red-fruited clade (99%) plus the consensus tree of the marker being tested for an introgression, with balanced sets of taxa (same taxa in each tree). A neutrality test [49] of marker C2_At1g73180 was carried out in DnaSP v. 5.10 [70] using marker TG11 as a control and *S. arcanum* as the out group.

## Abbreviations

ARS, Agricultural research service; COSII, Conserved Ortholog Set II; EMBL, European Molecular Biology Laboratory; ENA, European Nucleotide Archive; EST, Expressed sequence tag; NJ, Neighbor joining; GM, Genetically modified; PGRU, Plant Genetic Resources Unit; PUT, PlantGDB-assembled Unique Transcipts; QTL, Quantitative trait loci; RFLP, Restriction fragment length polymorphism; SGN, Sol genomics network; SI, Self-incompatible; SNP, Single nucleotide polymorphism; U, Unigene; USDA, United States Department of Agriculture.

## Competing interests

The authors declare that they have no competing interests.

## Authors’ contributions

JAL and LDR conceived and designed the study. JAL acquired, analyzed and interpreted the data, and drafted the manuscript. LDR gave final approval of the version to be published. Both authors read and approved the final manuscript.

## Supplementary Material

Additional file 1 **Table S1.**Virtual mapping of 47 sequence-based markers. BLASTN results of 47 markers against tomato whole genome shotgun chromosomes from version 2.40 of the WUR assembly, current as of 10 Jan, 2012. **Table S2.**Evolutionary relationships among taxa at 47 markers. Bootstrap support and evolutionary divergence of 47 tomato markers sequenced in wild and cultivated tomato accessions. Marker names in bold were previously hypothesized as carrying introgressions. Click here for file

Additional file 2 **Clustering and network analyses of tomato genotypes.** Neighbor joining trees for 47 markers and hybridization networks for nine markers sampled from wild and cultivated tomato. Click here for file
