# Supplementary material for: Evidence of cryptic introgression in tomato (Solanum lycopersicum L.) based on wild tomato species alleles
Source: BMC Plant Biol. 2012 Aug 7;12:133. doi: 10.1186/1471-2229-12-133 (PMC3462117; doi:10.1186/1471-2229-12-133)

LeSNP1
437_2

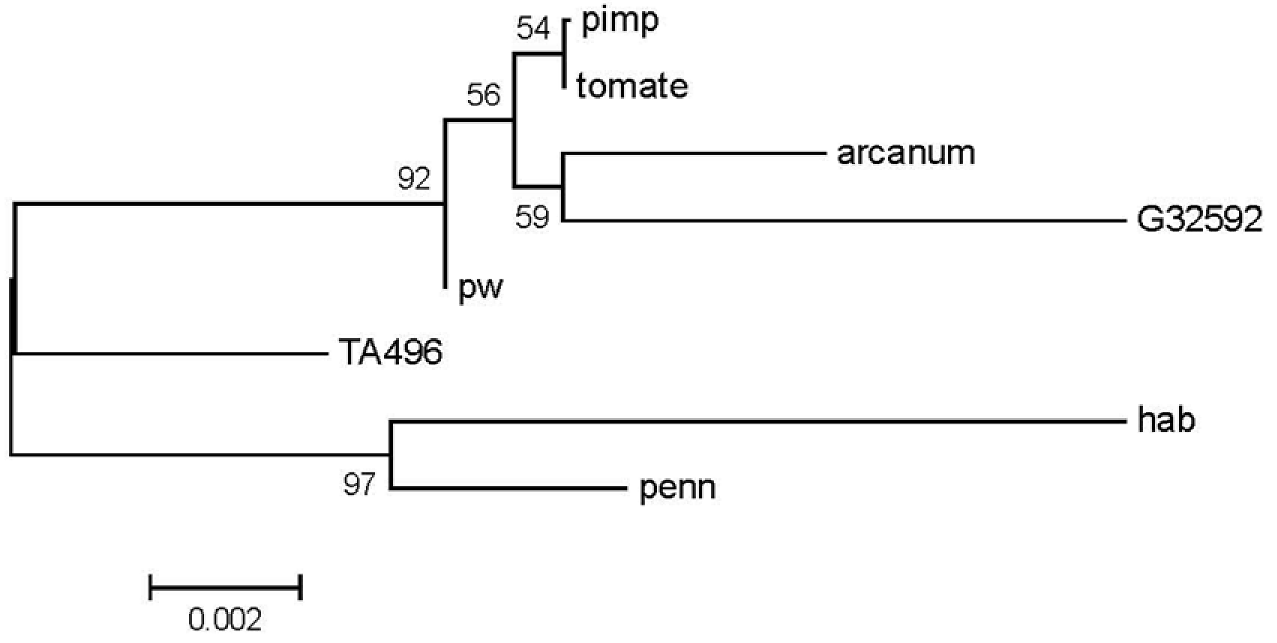
54
pimp
tomate
56
arcanum
92
59
G32592
pw
TA496
hab
97
penn
0.002

LeSNP3
3300_2

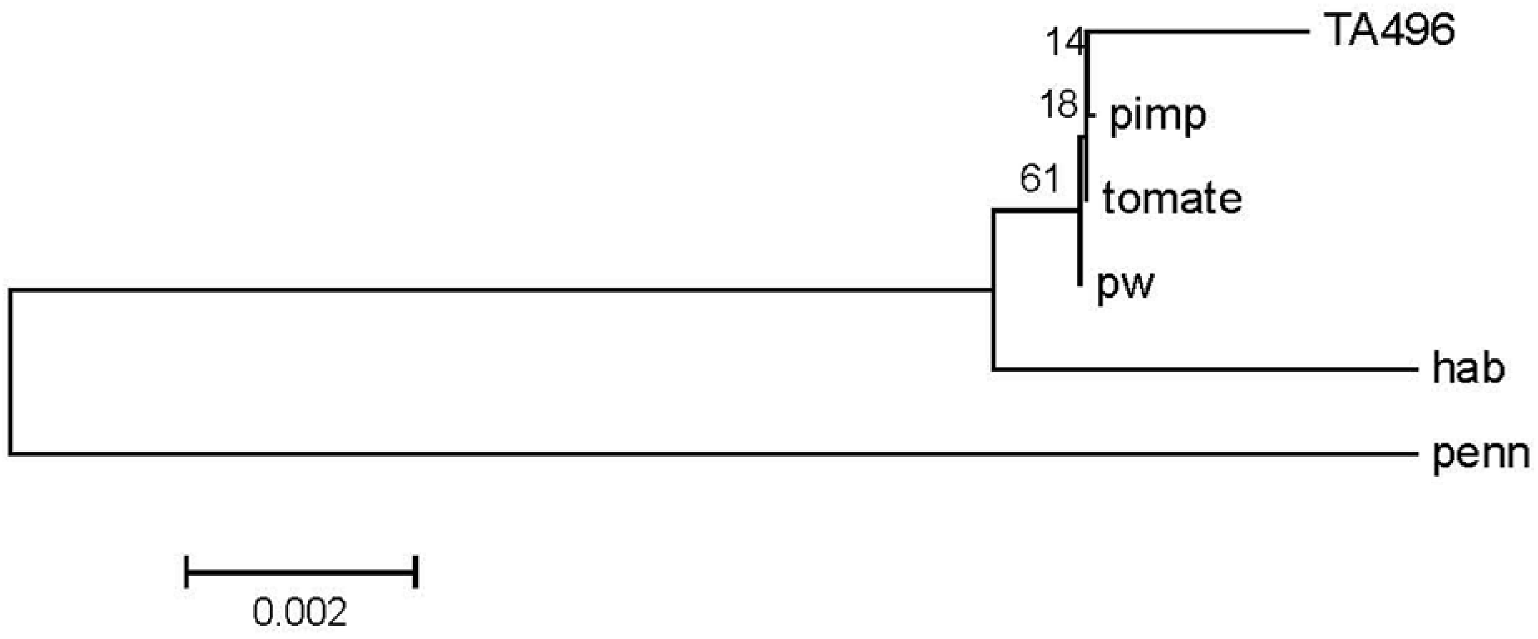

LeSNP4
4301_3

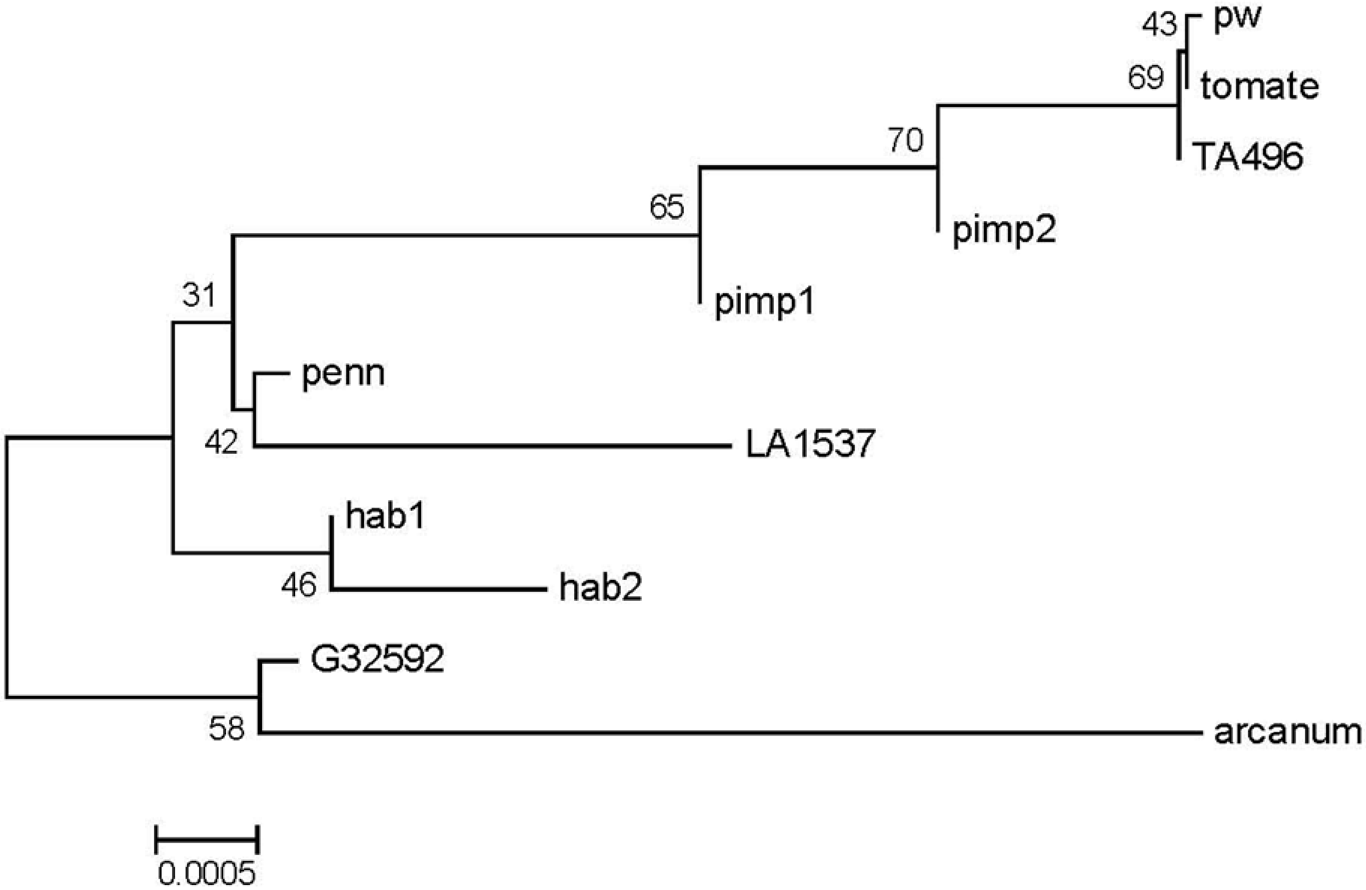

LeSNP5
3155_3

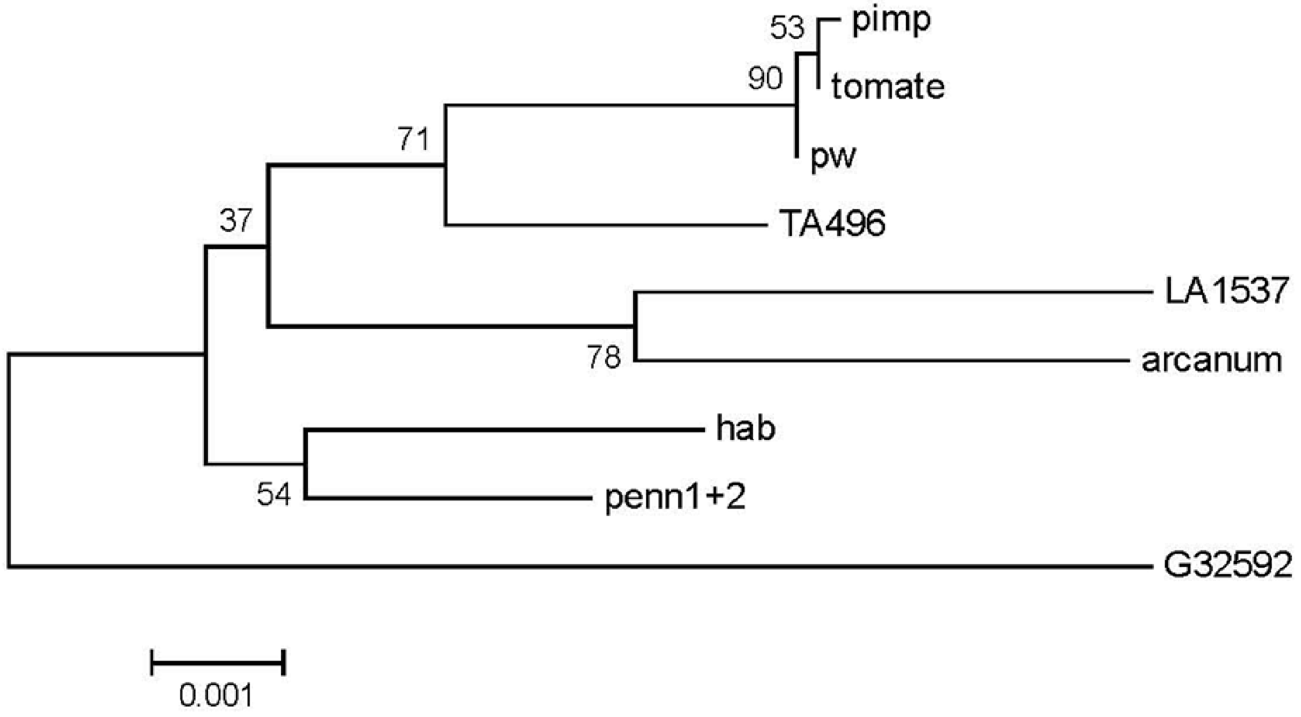

LeSNP6
1260_2

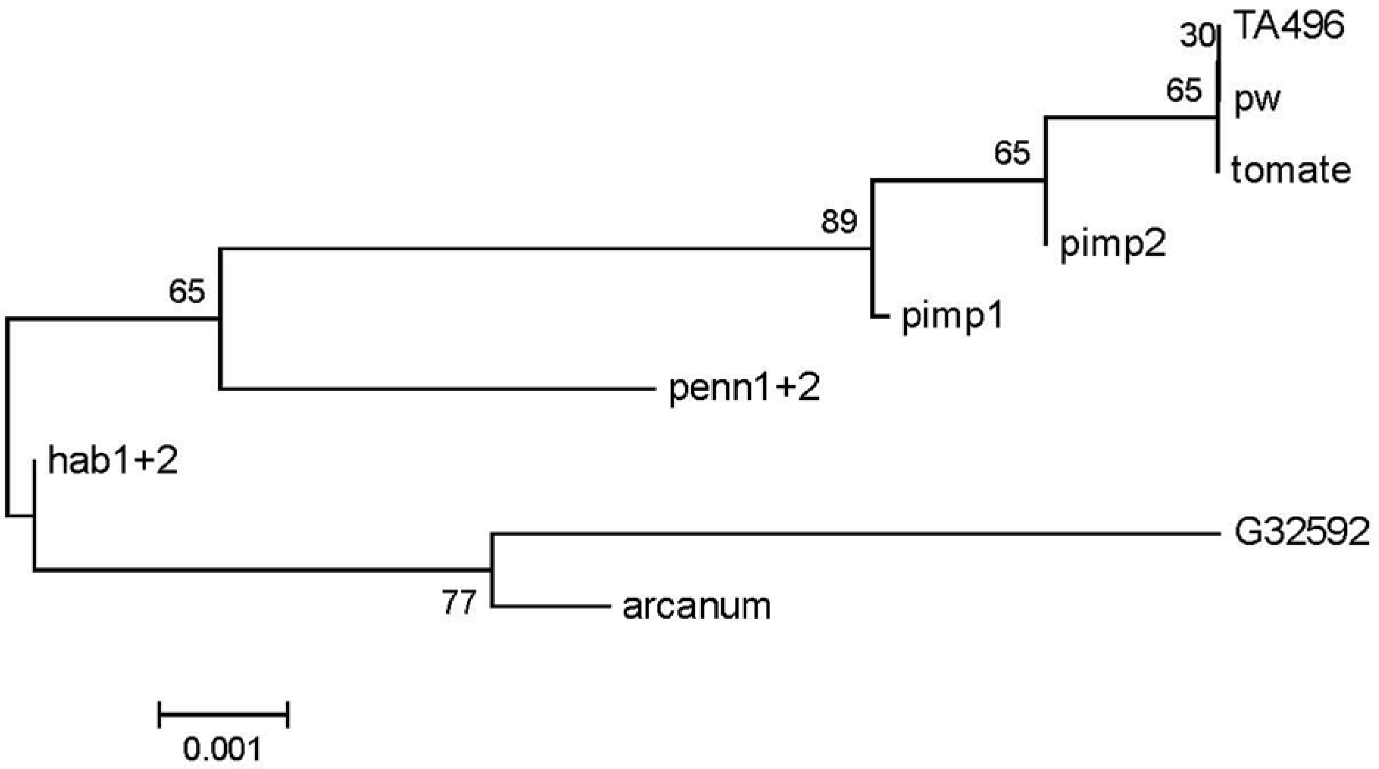

LeSNP7
3332_3

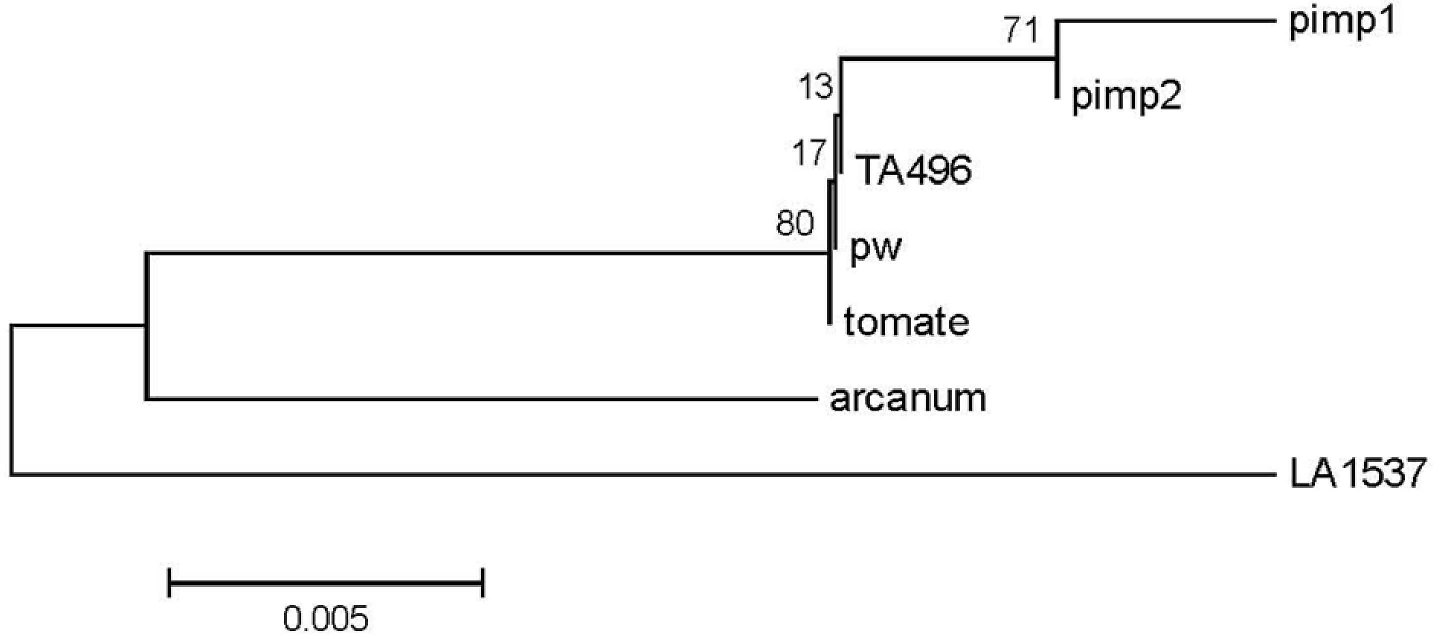

LeSNP9b
2534_1b

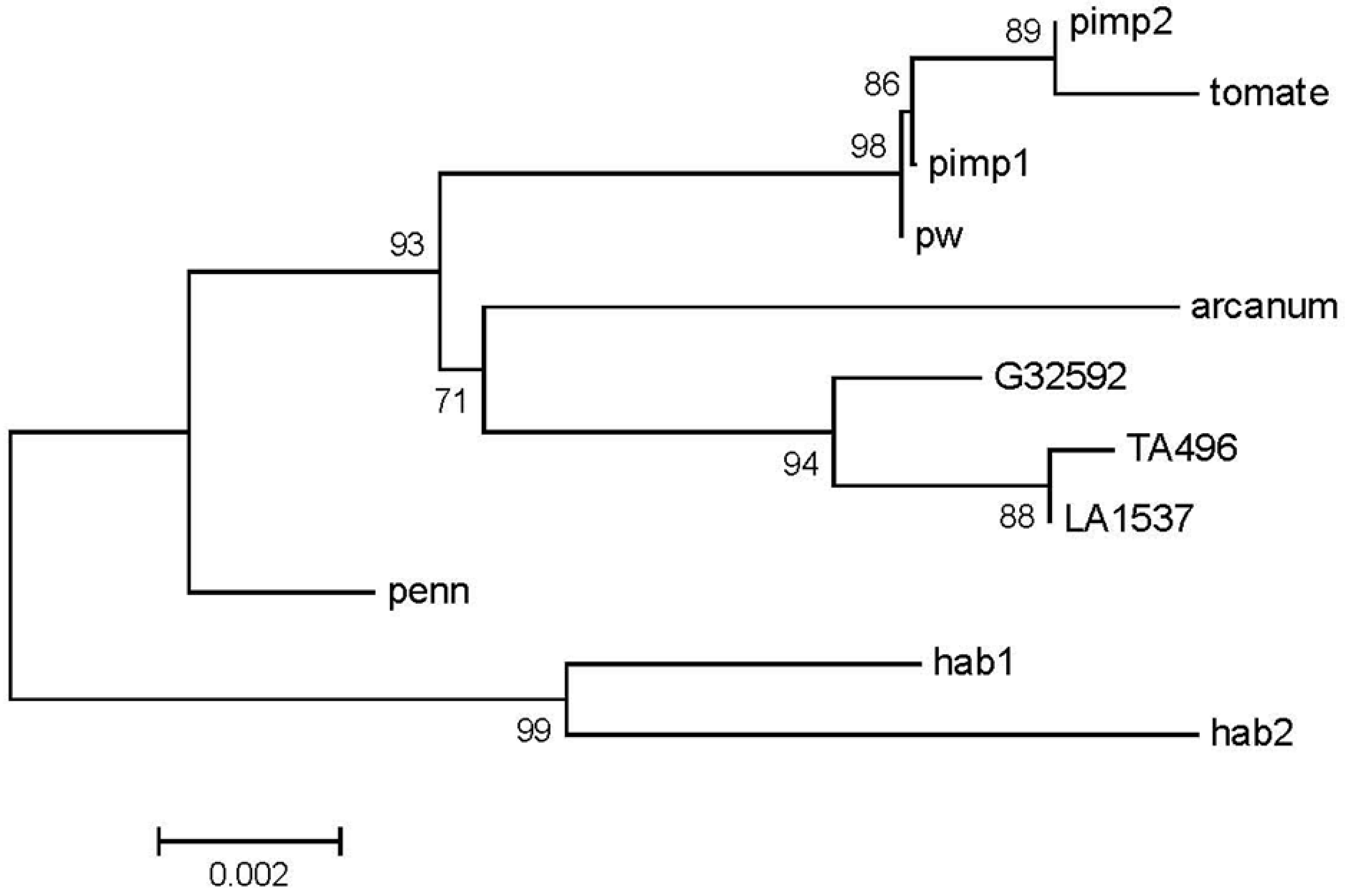

LeSNP10
2325_3

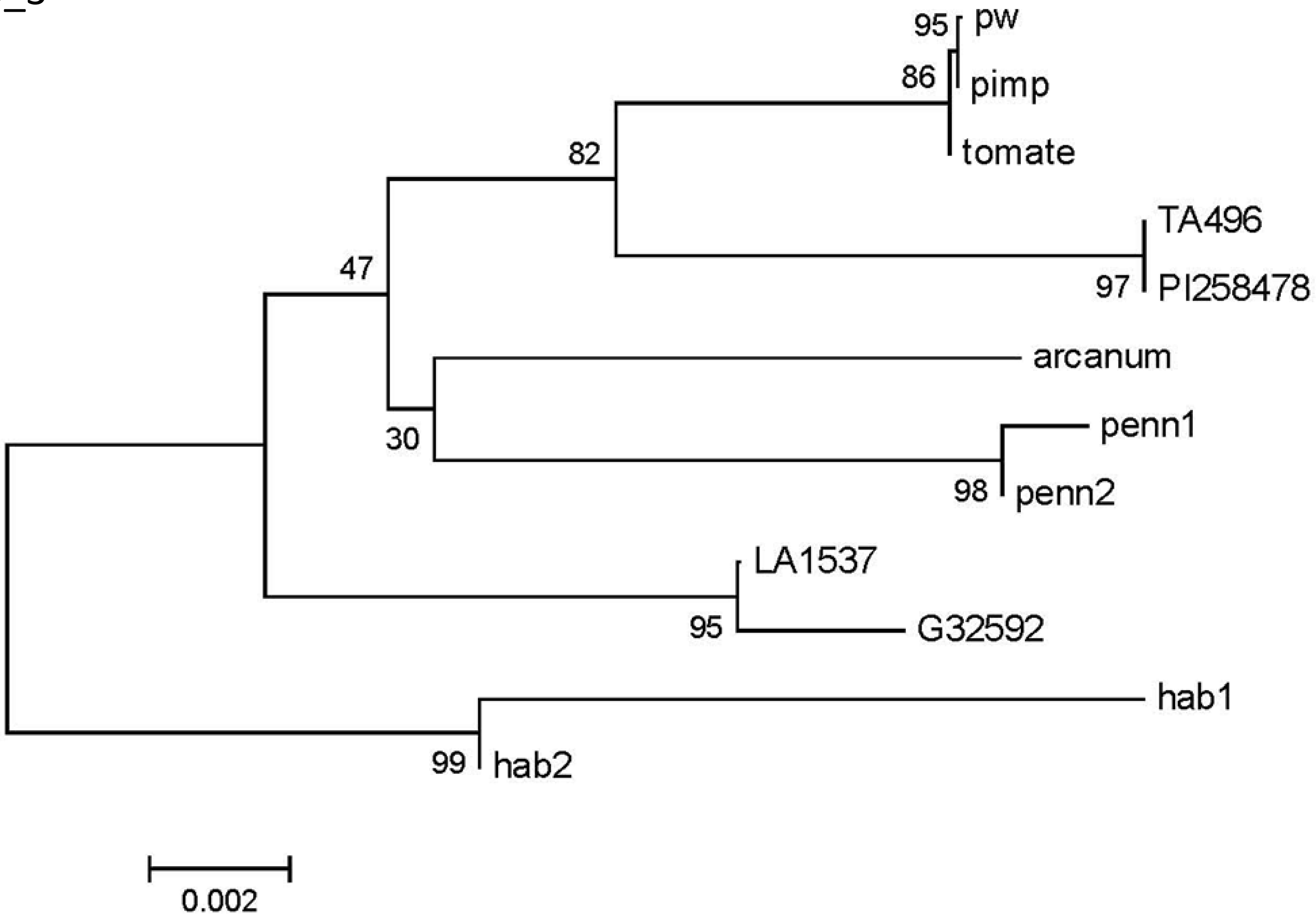

LeSNP13
1287_1

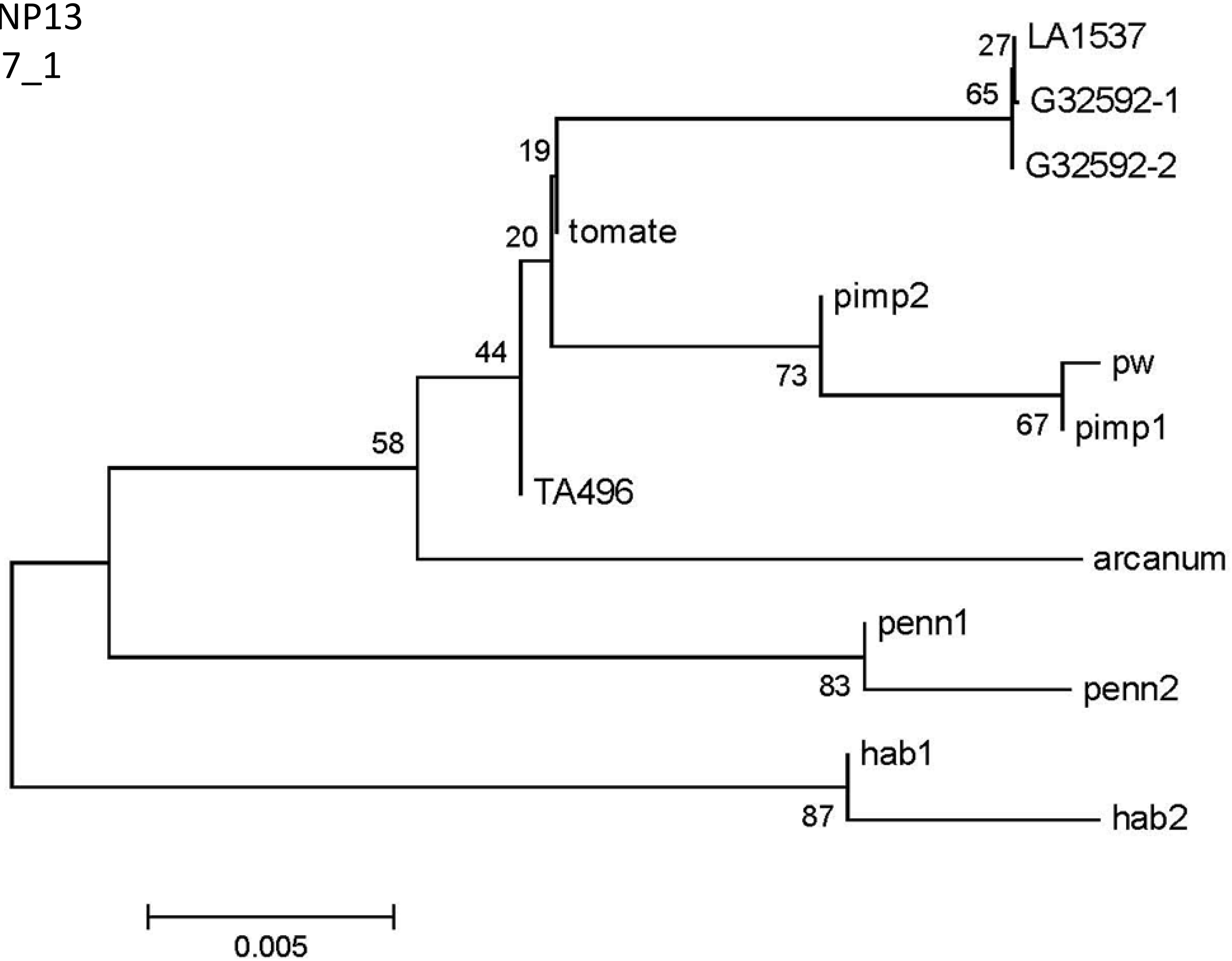

LeSNP16
1589_1

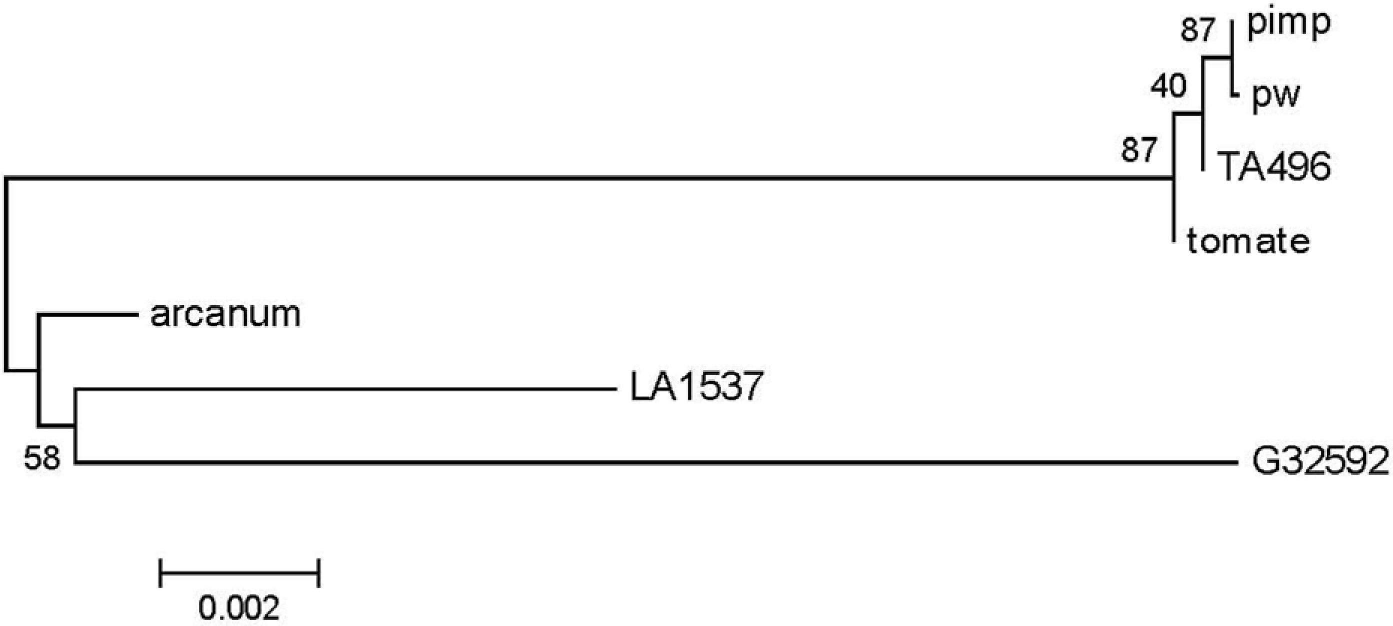

LeSNP17
1675_1

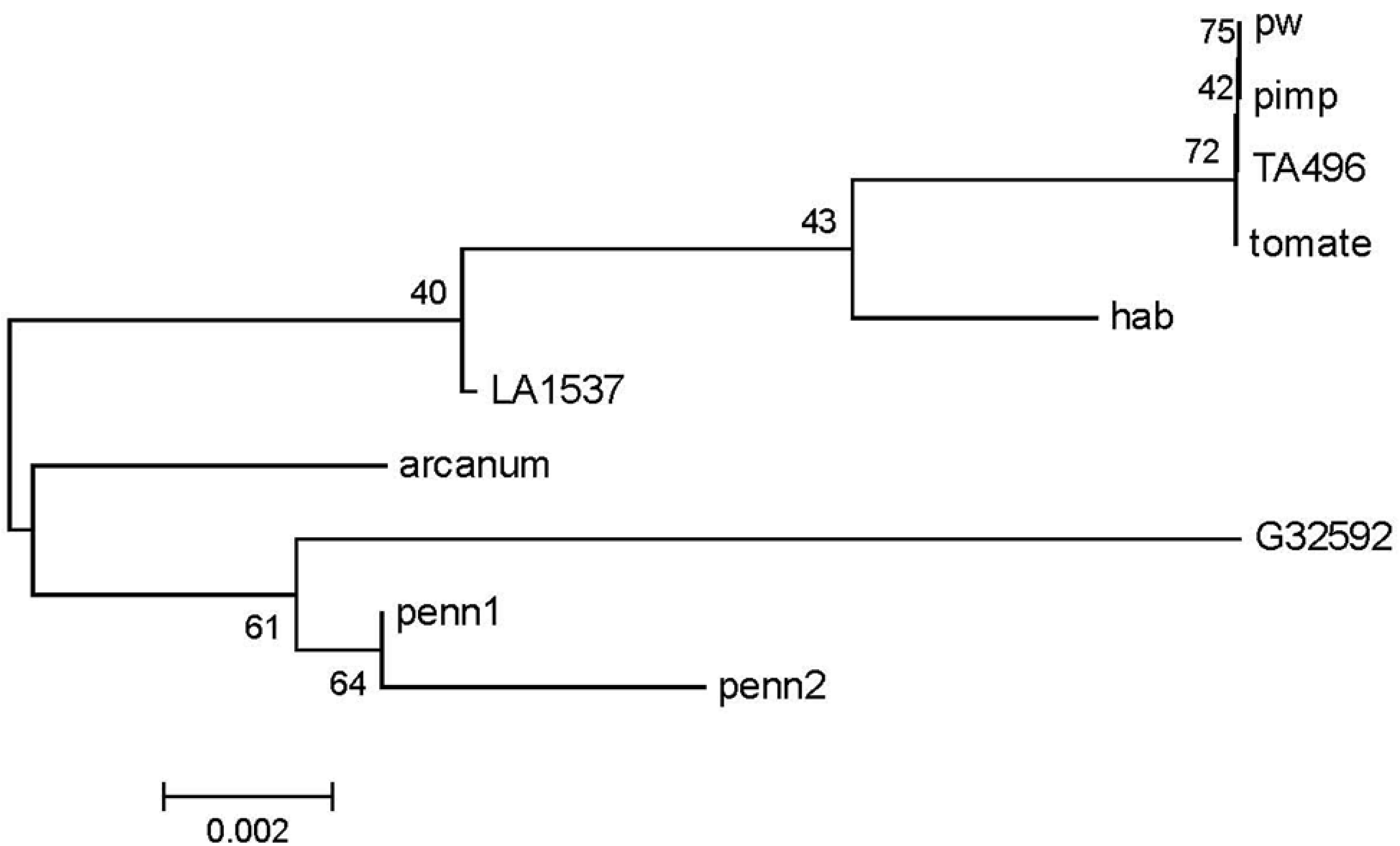

LeSNP19
1724_1

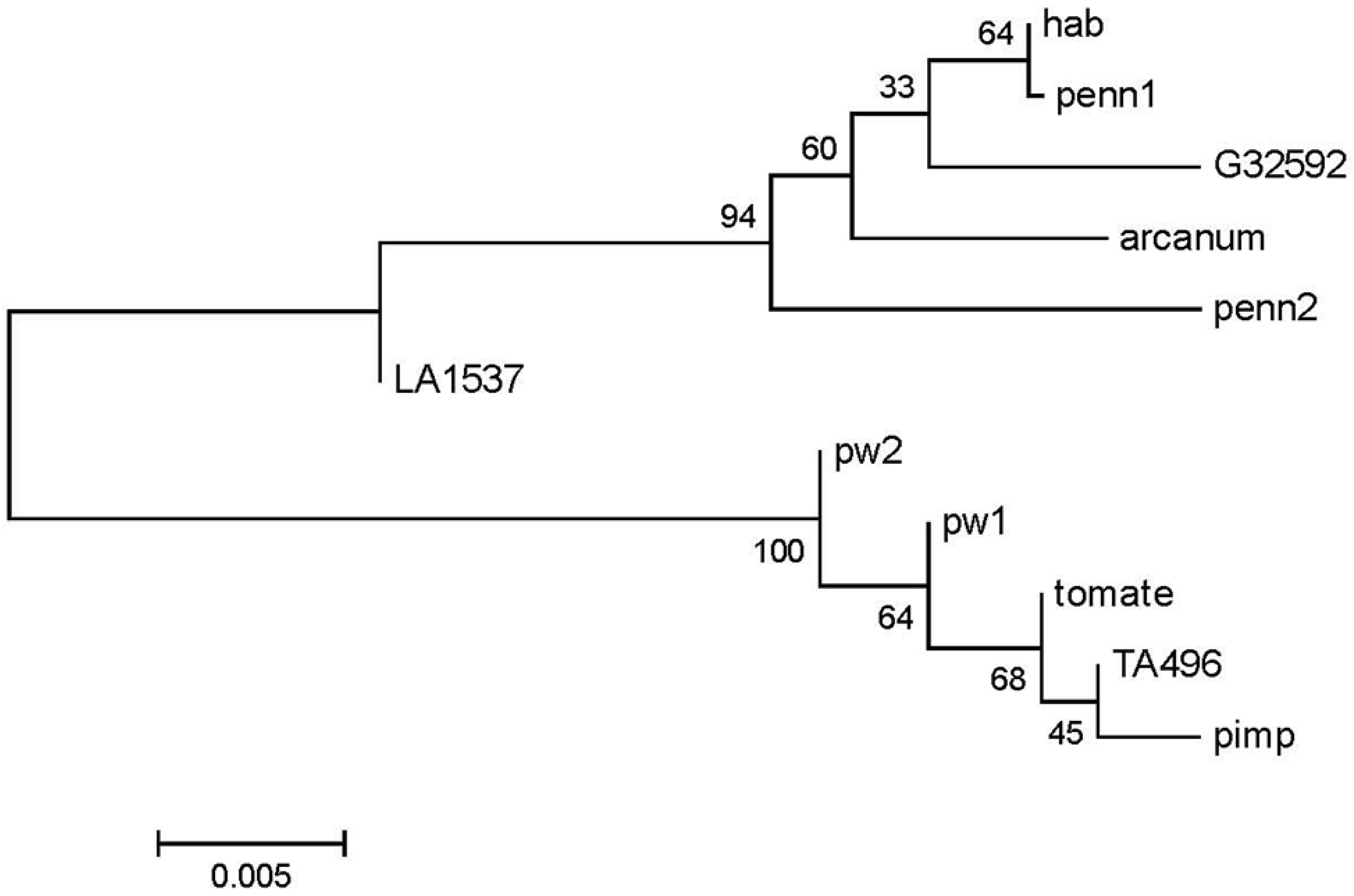

LeSNP21
1863_3

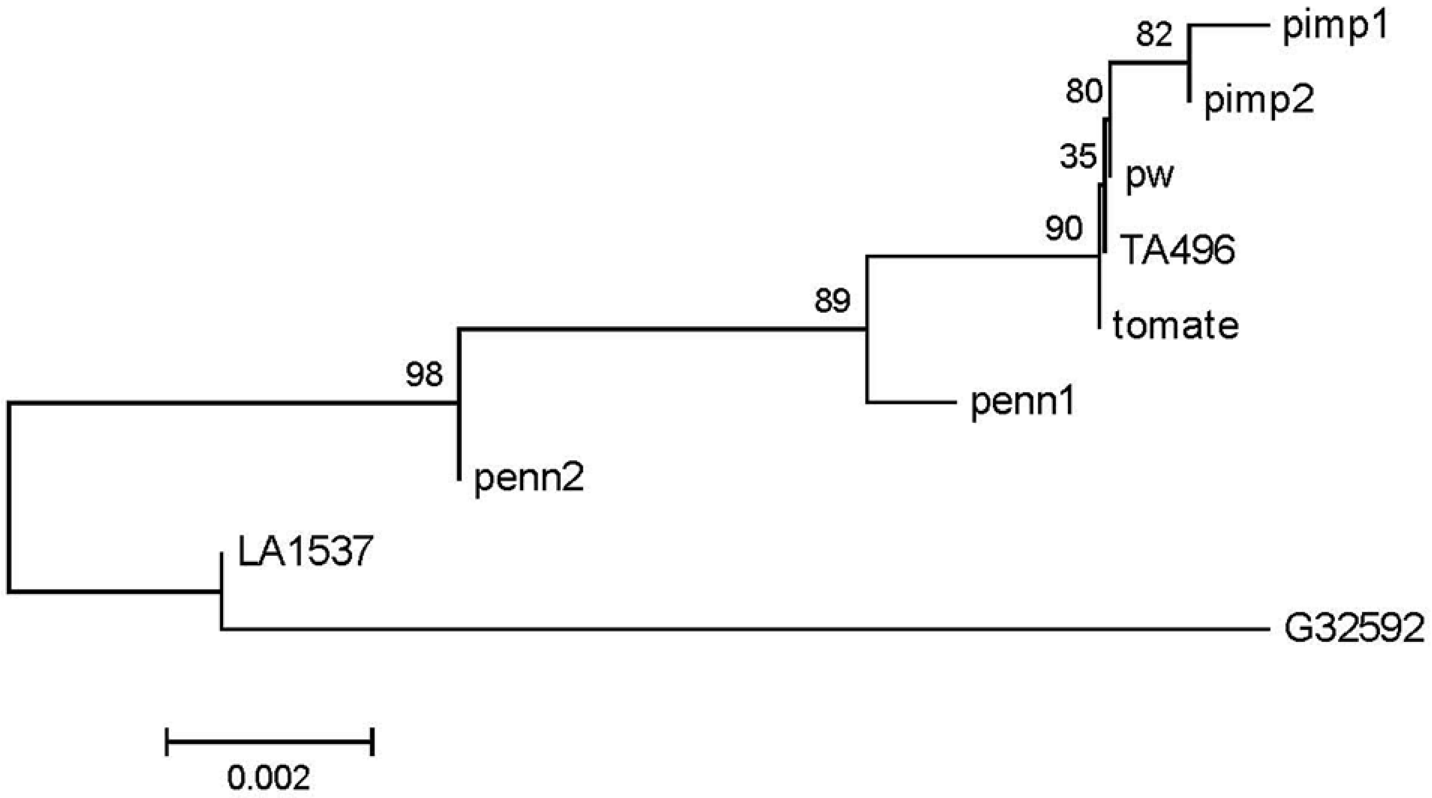

LeSNP24
2189_1

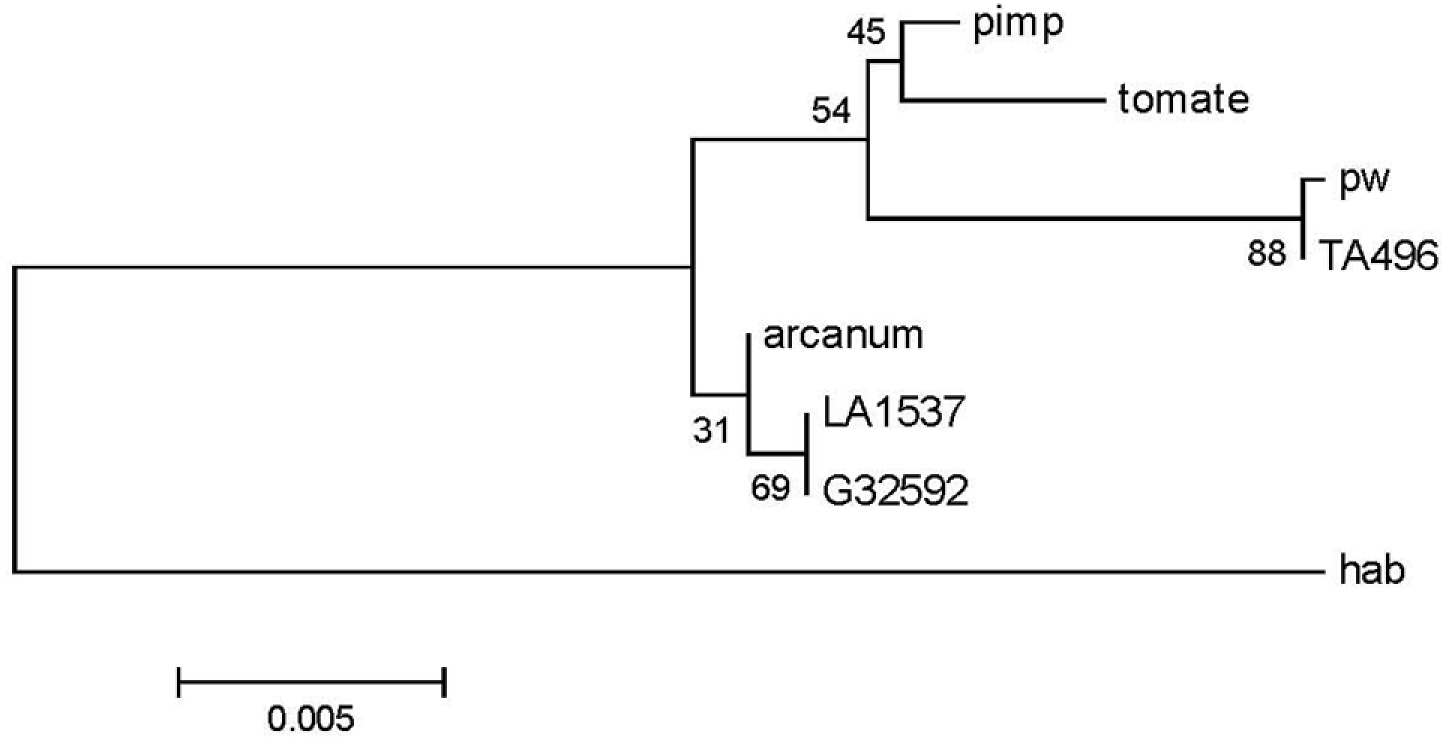

LeSNP25
220_1

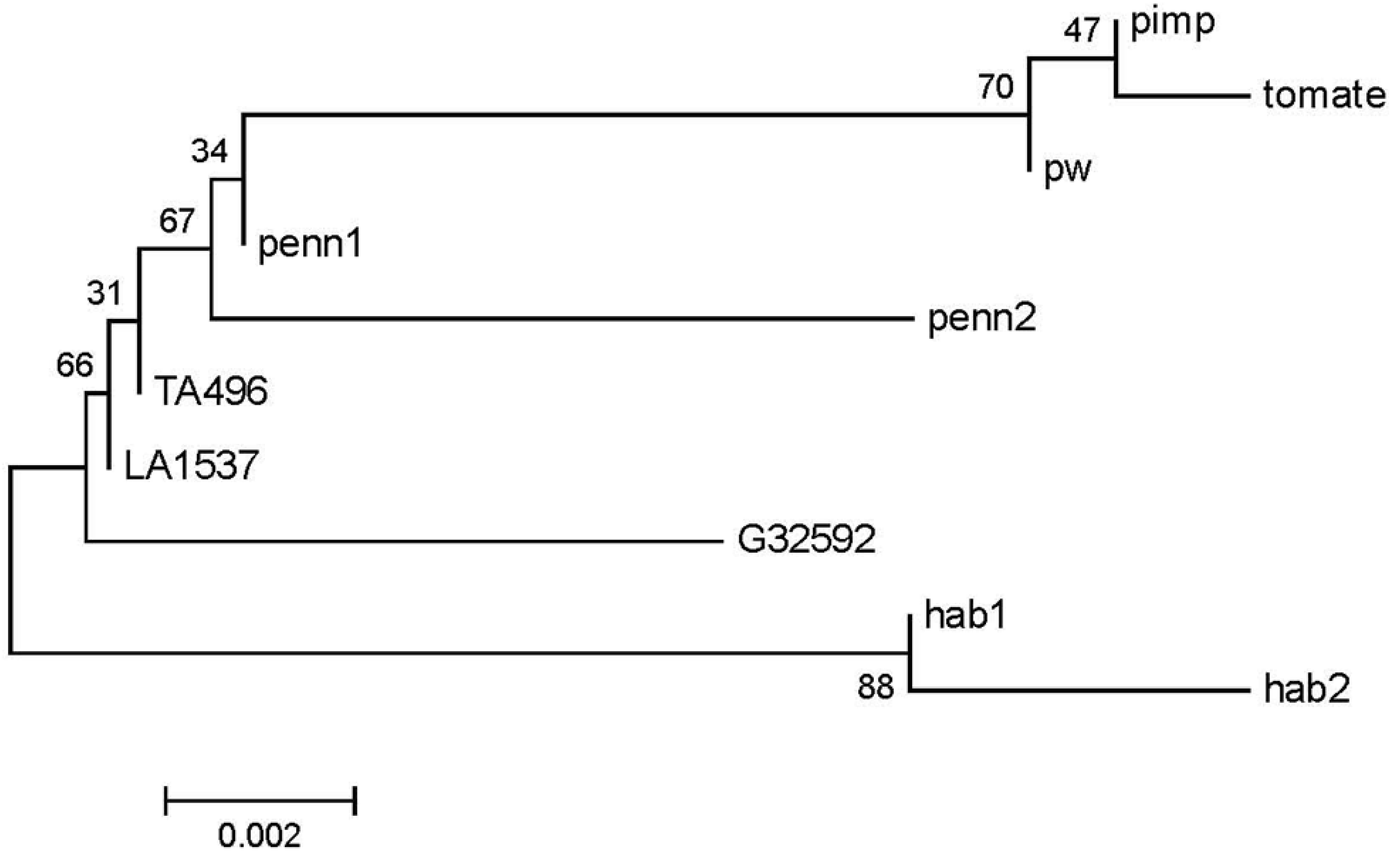

LeSNP27
2280_1

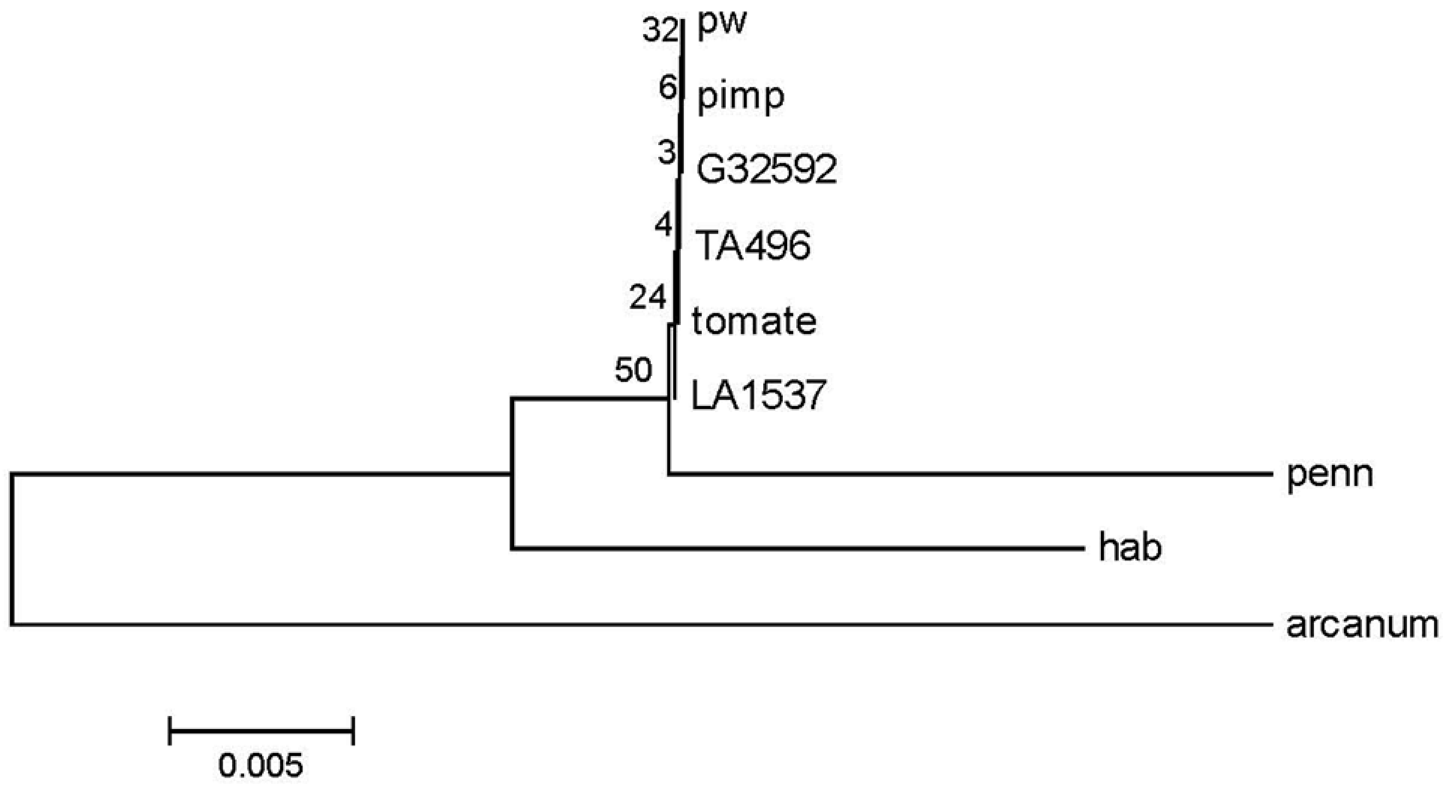

LeSNP28b
241_2b

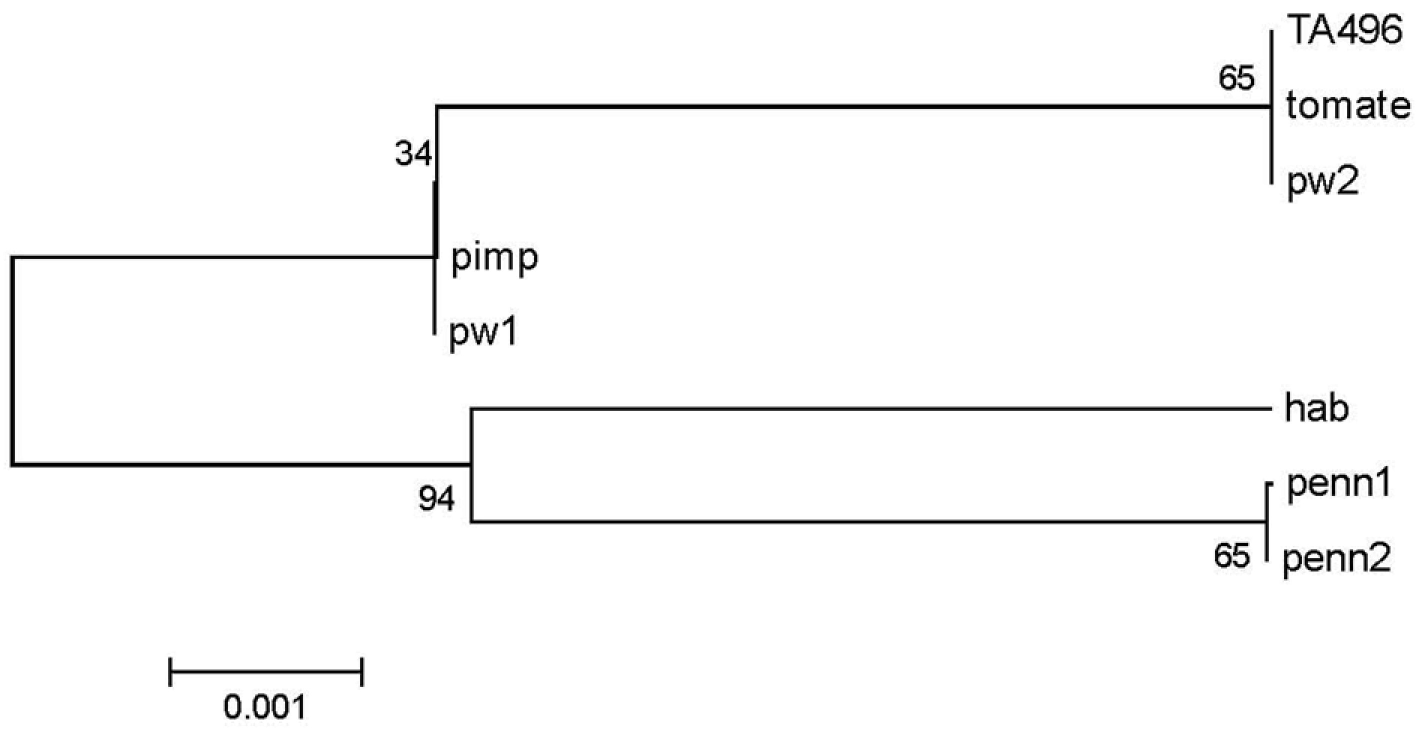

LeSNP30
2486_1

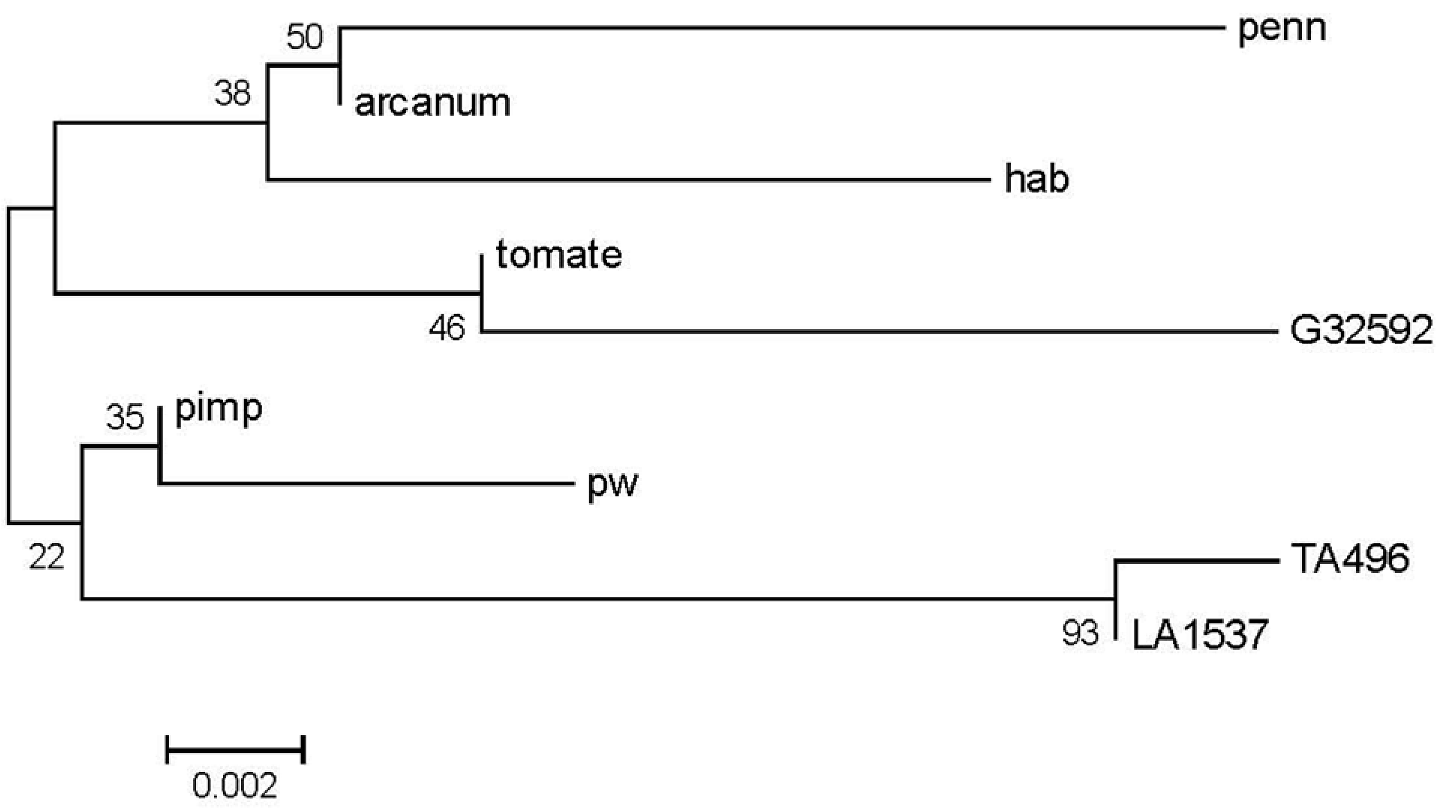

LeSNP32
2582_1

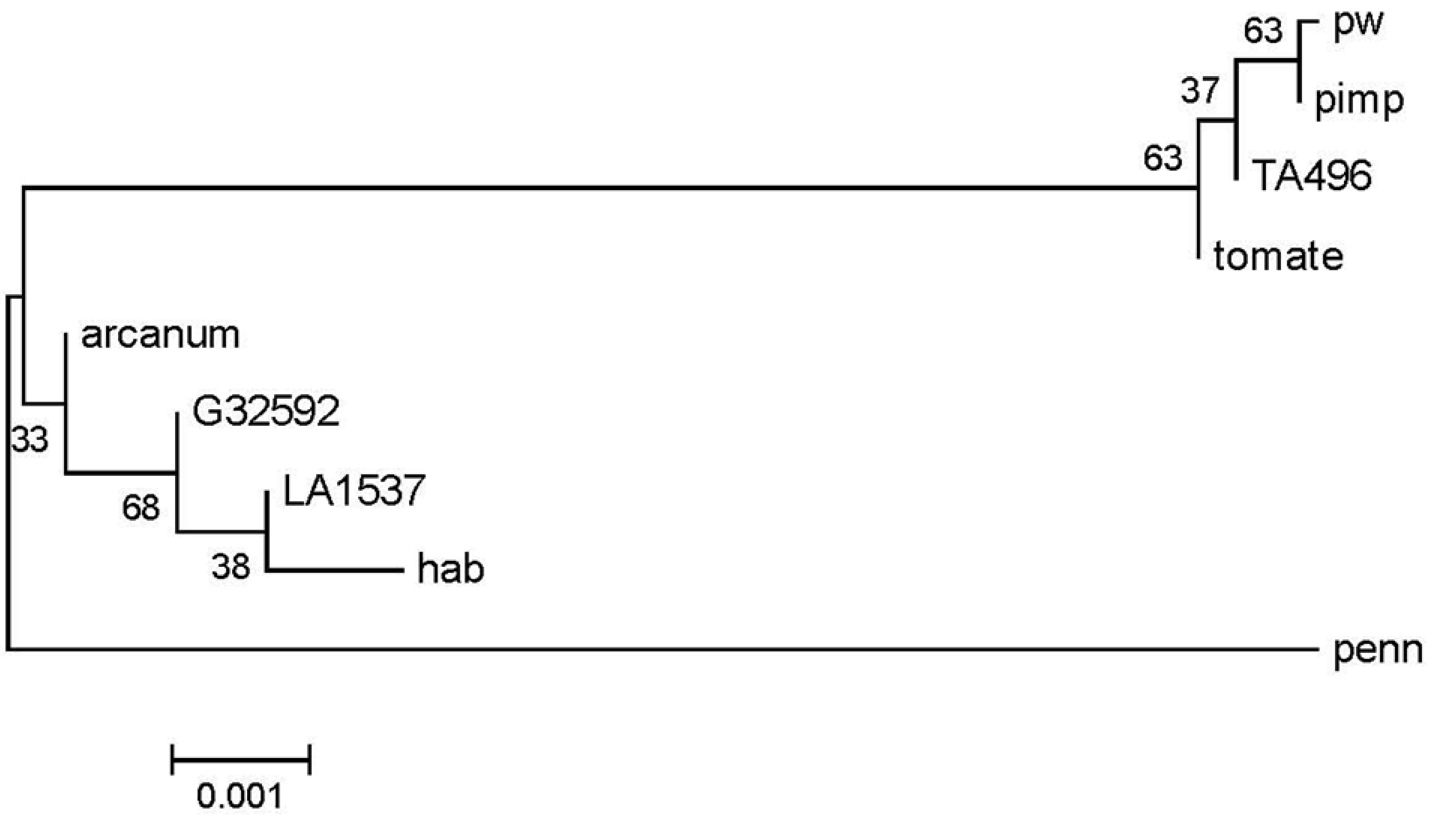

LeSNP33
2719_1

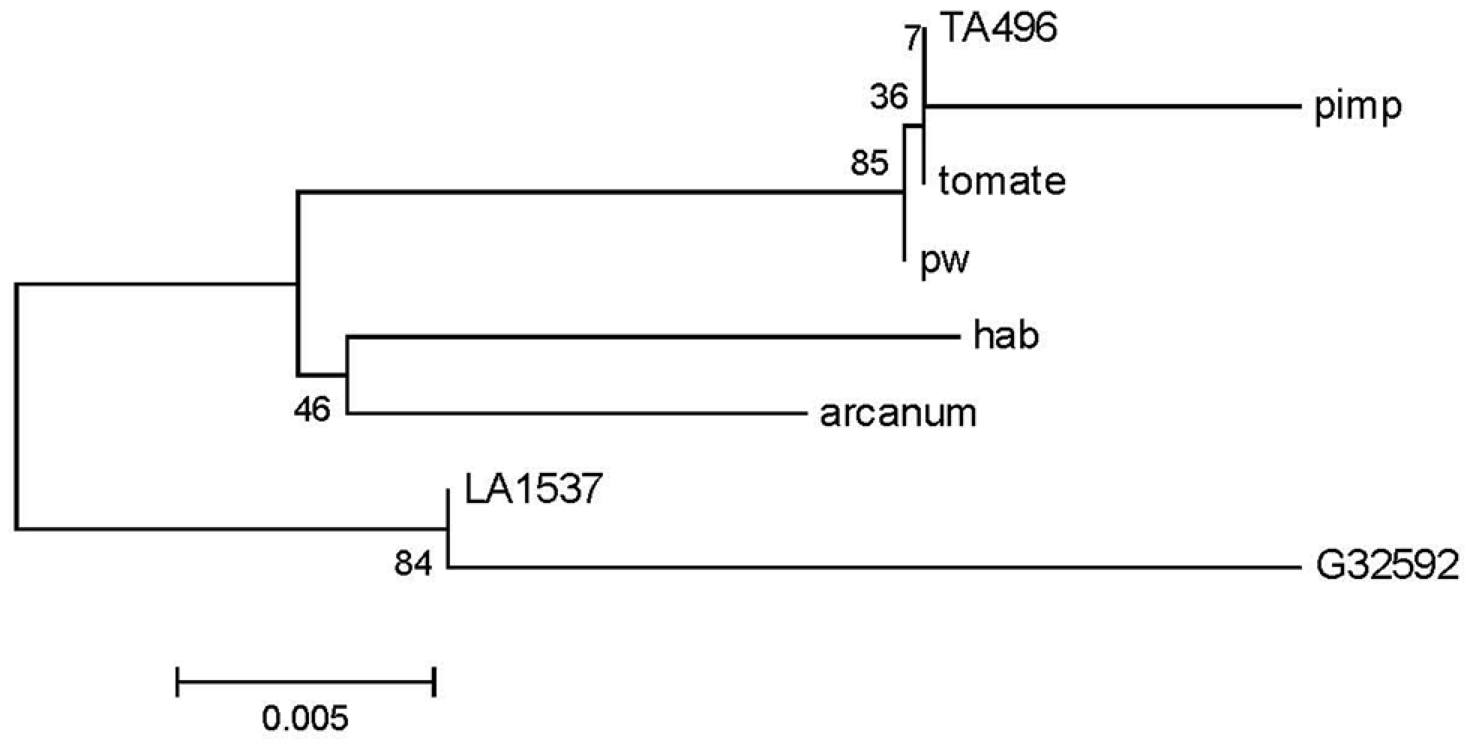

LeSNP34
2819_5

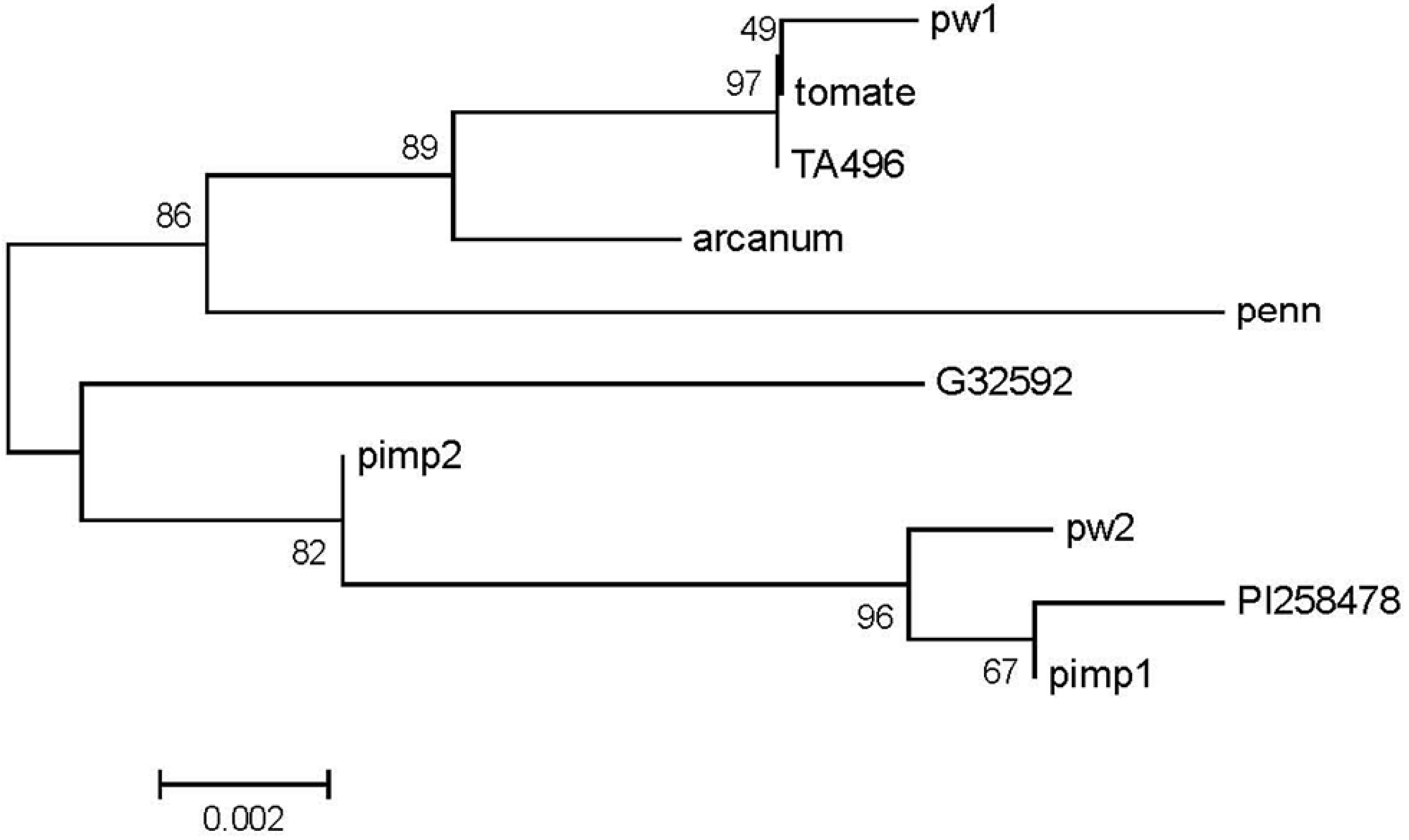

LeSNP35b
2875_4b

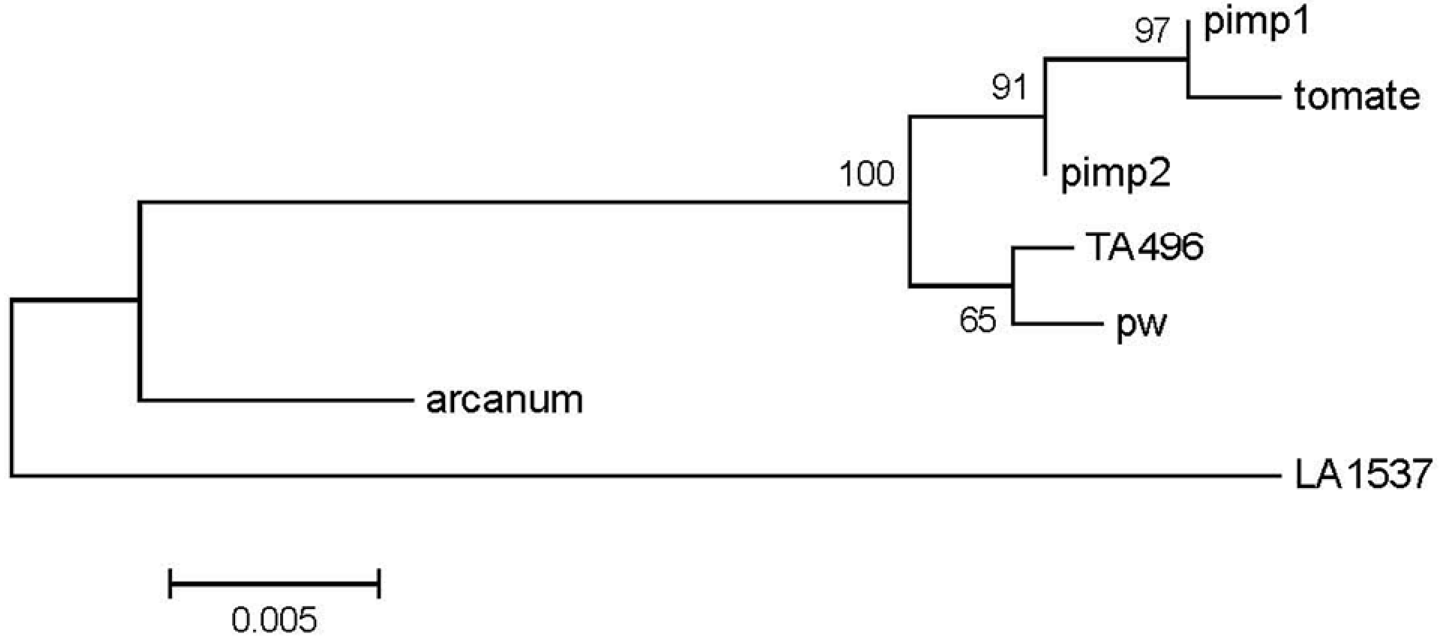

LeSNP40b
296_1b

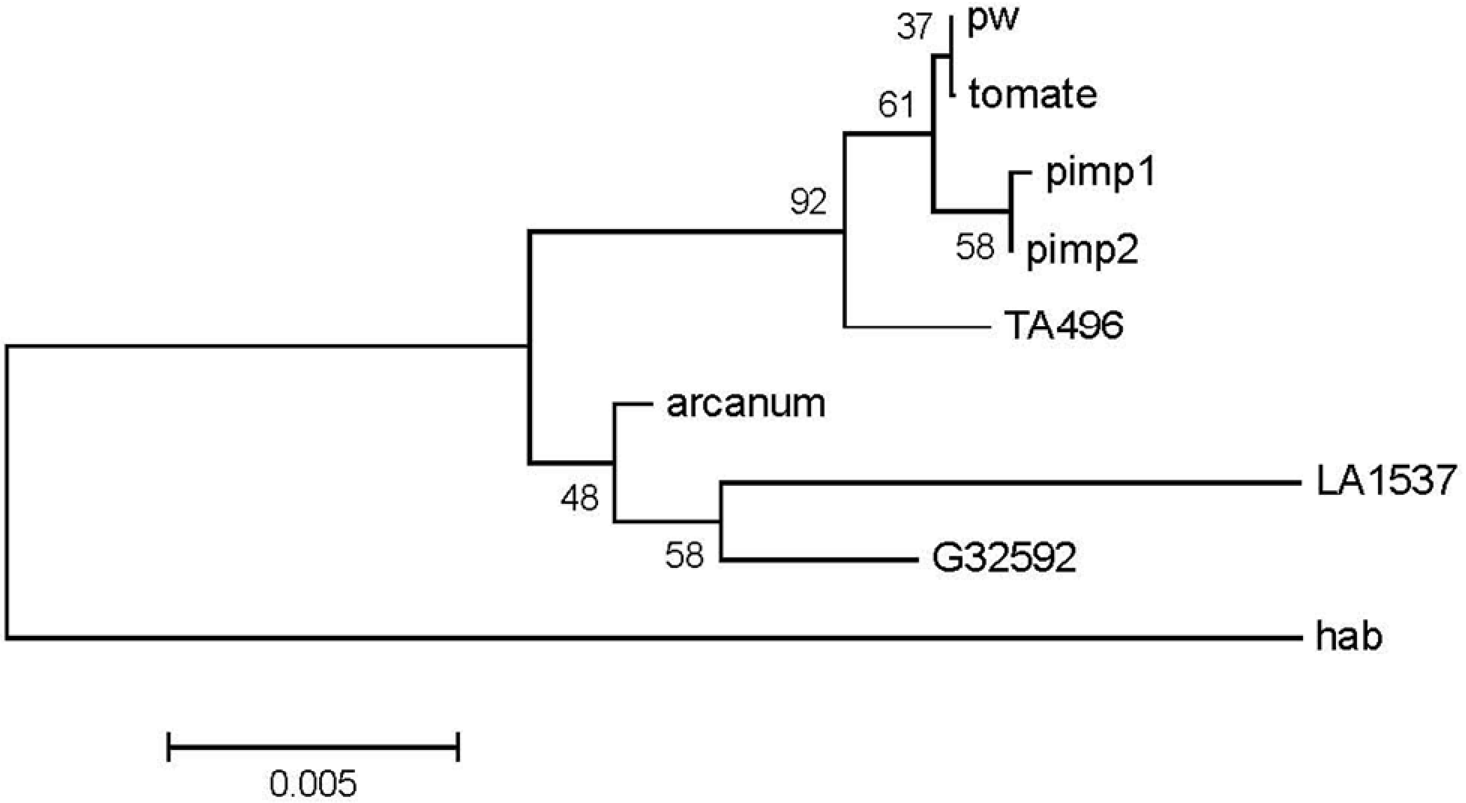

COS1
U146140

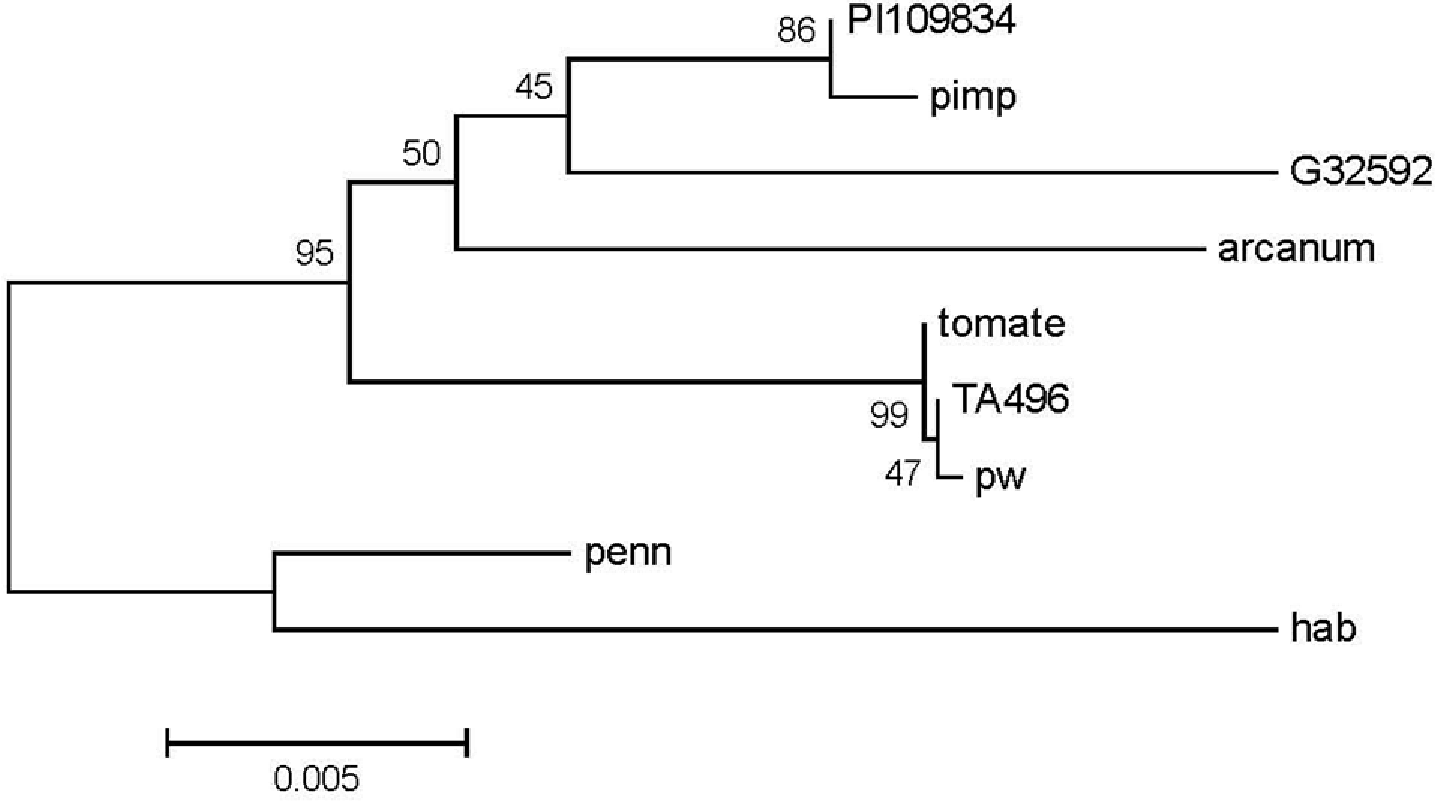

COS2
C2_At1g13380

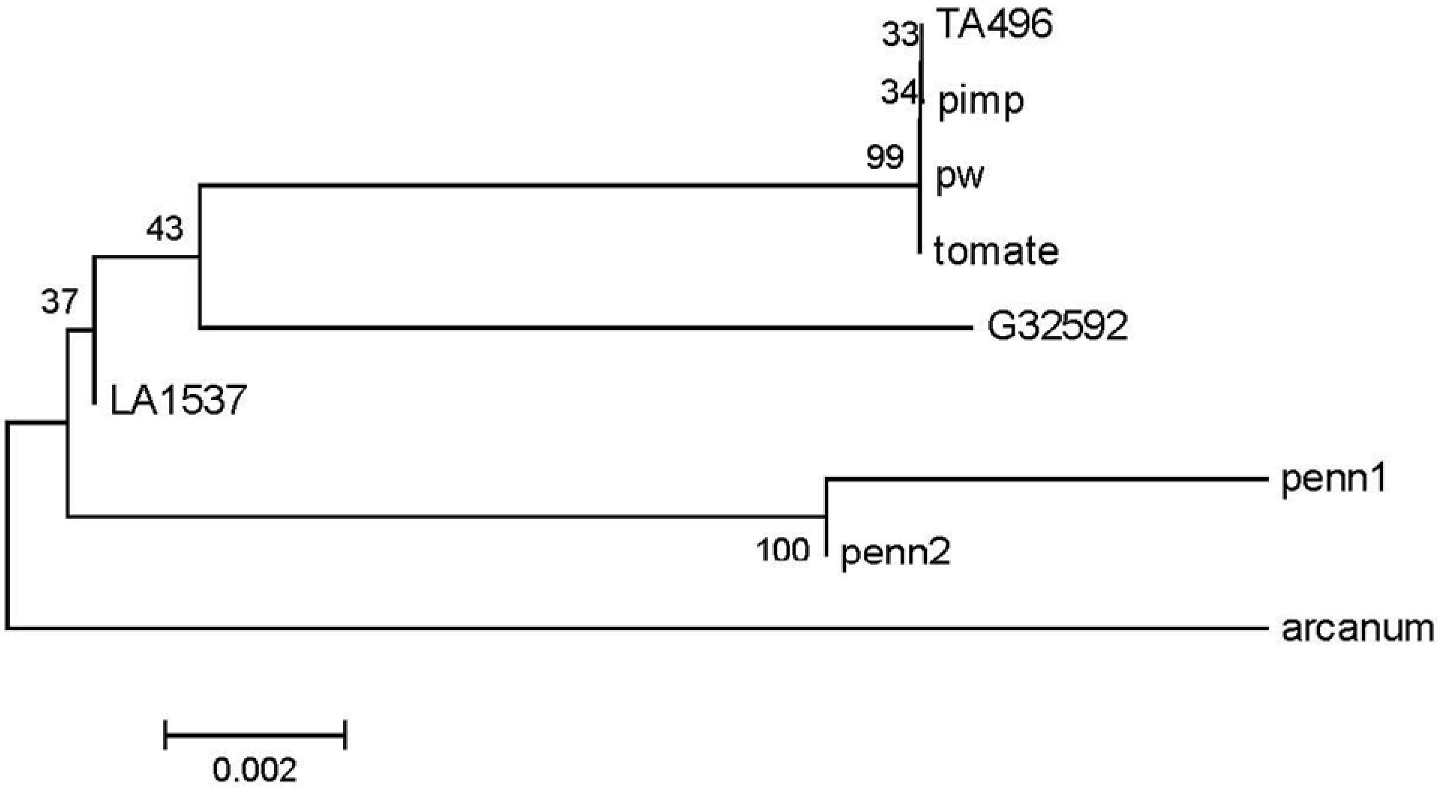

COS3
C2_At1g14000

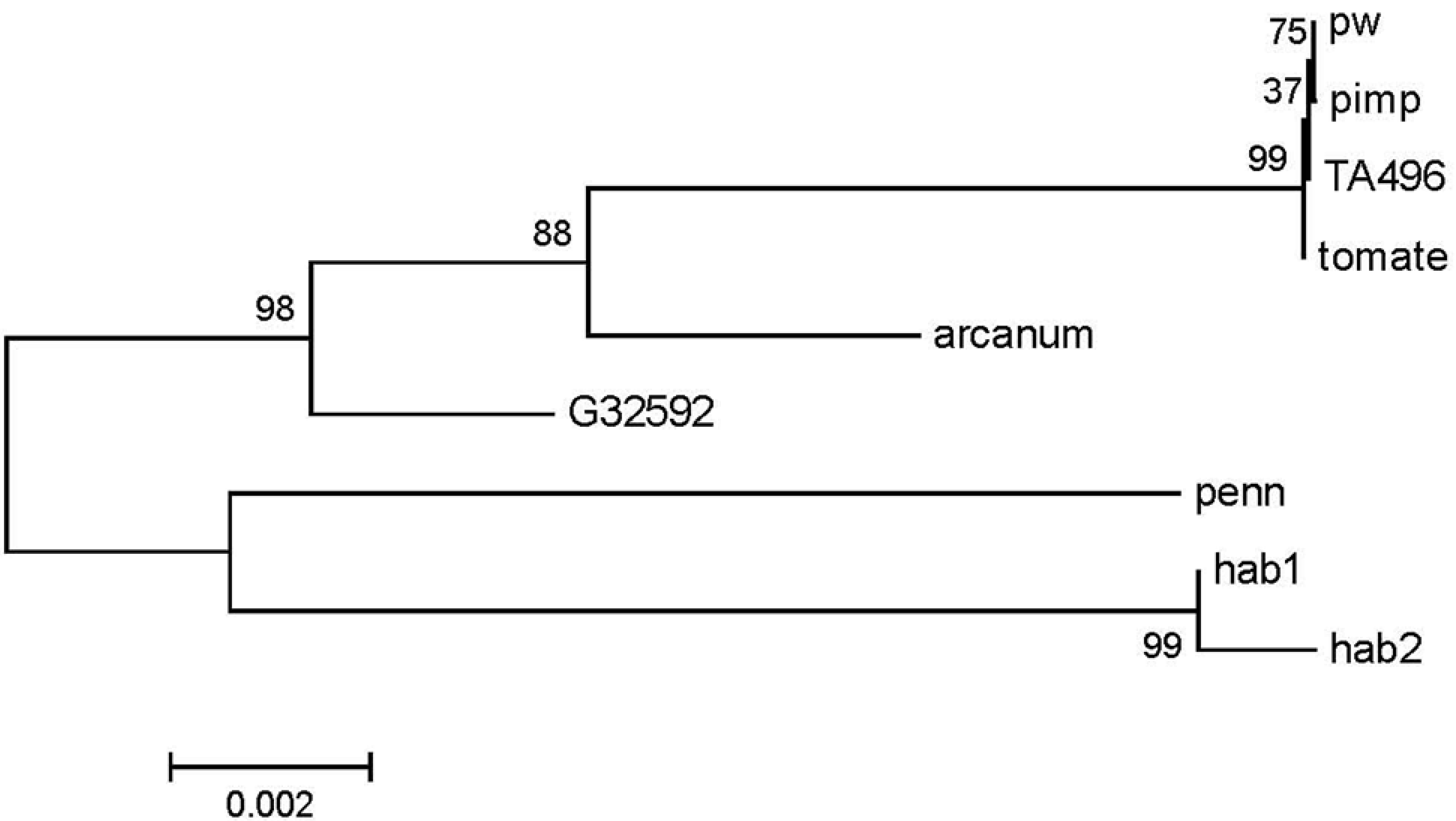

COS4
C2_At1g20050

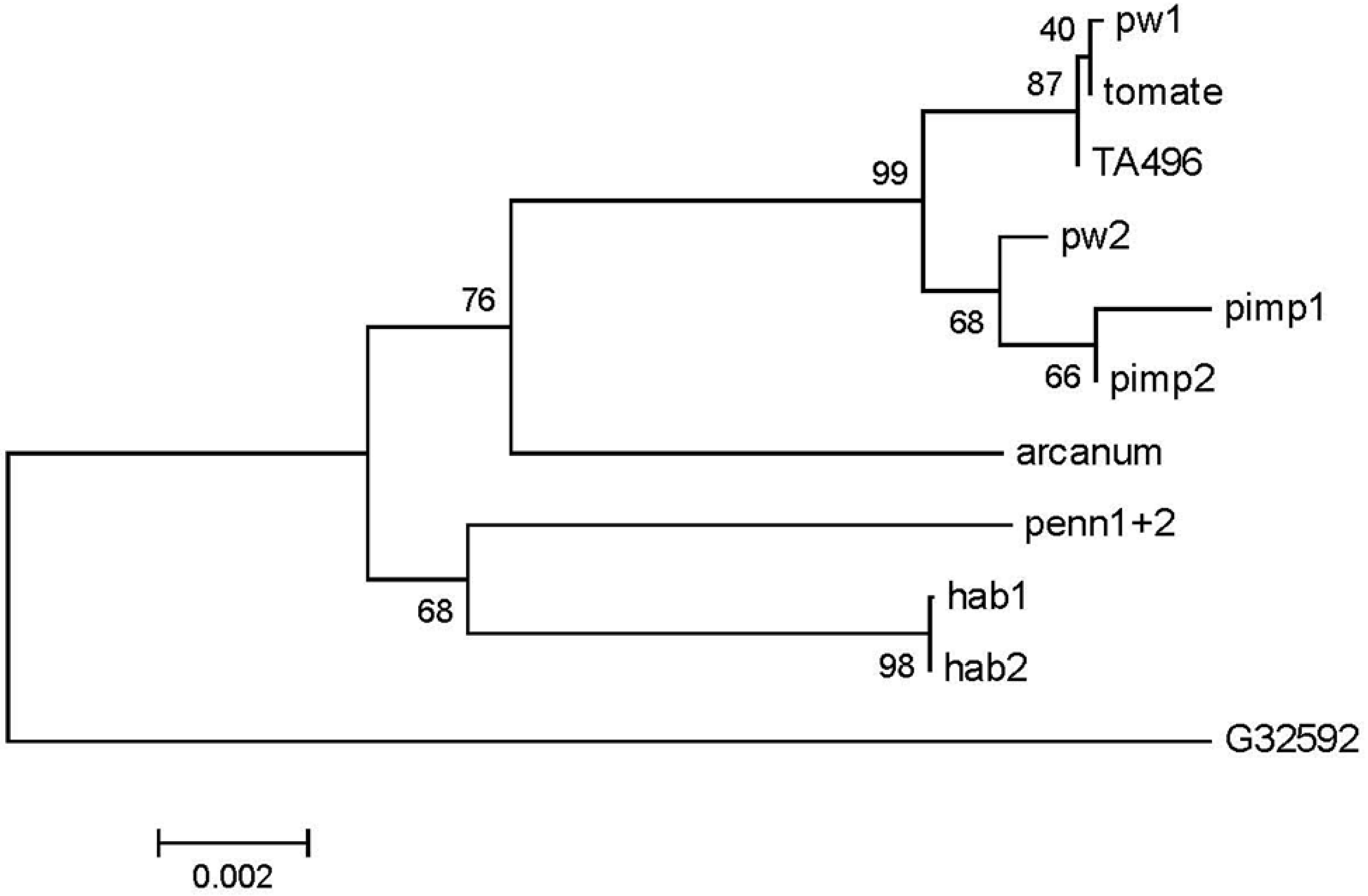

COS5
C2_At1g32130

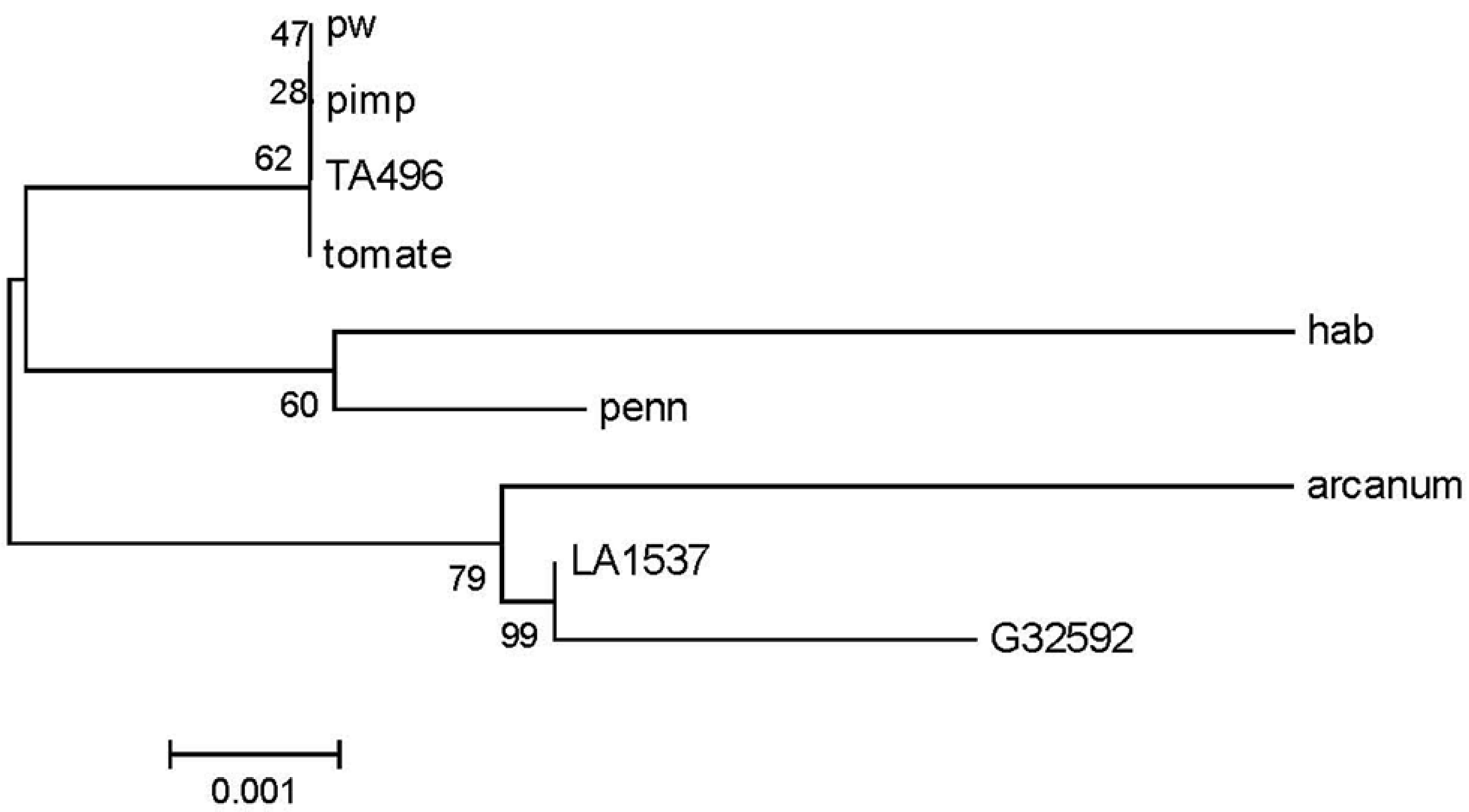

COS7
C2_At1g44575

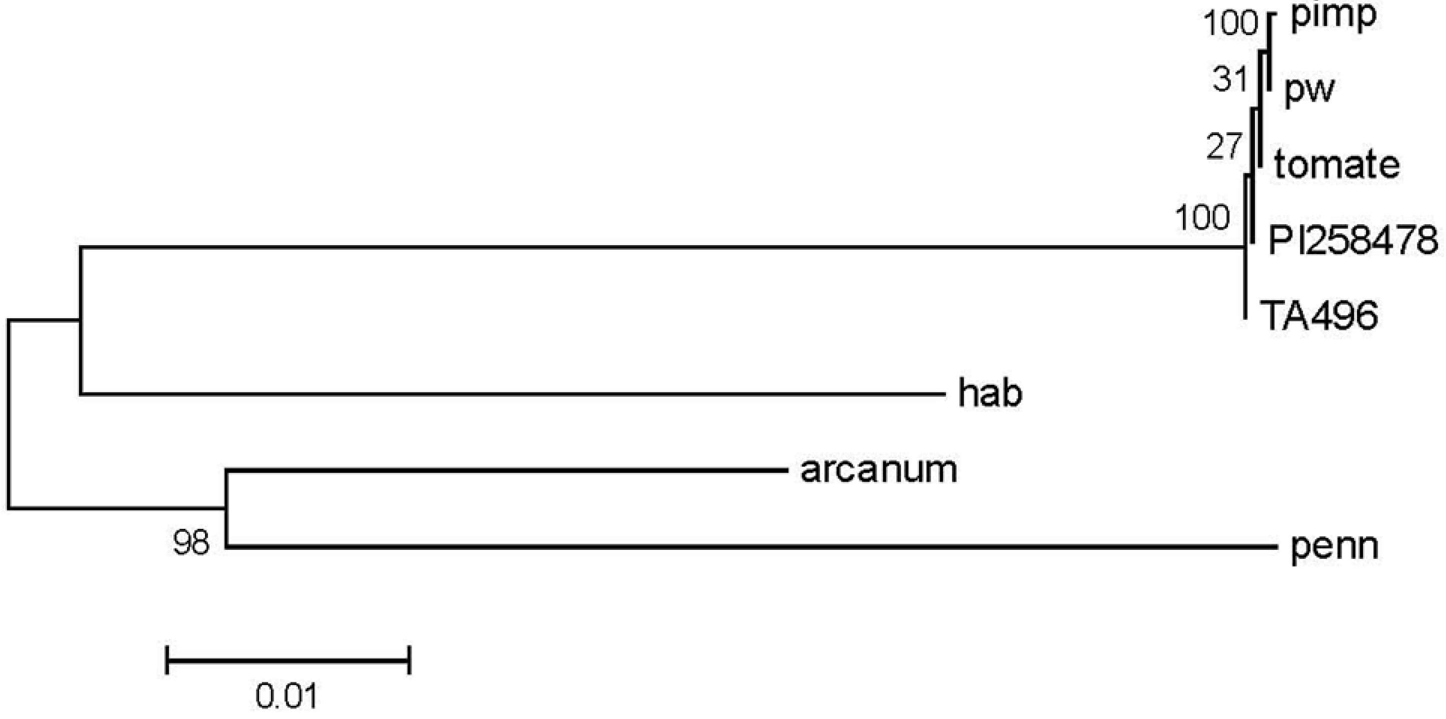

COS9
C2_At1g50020

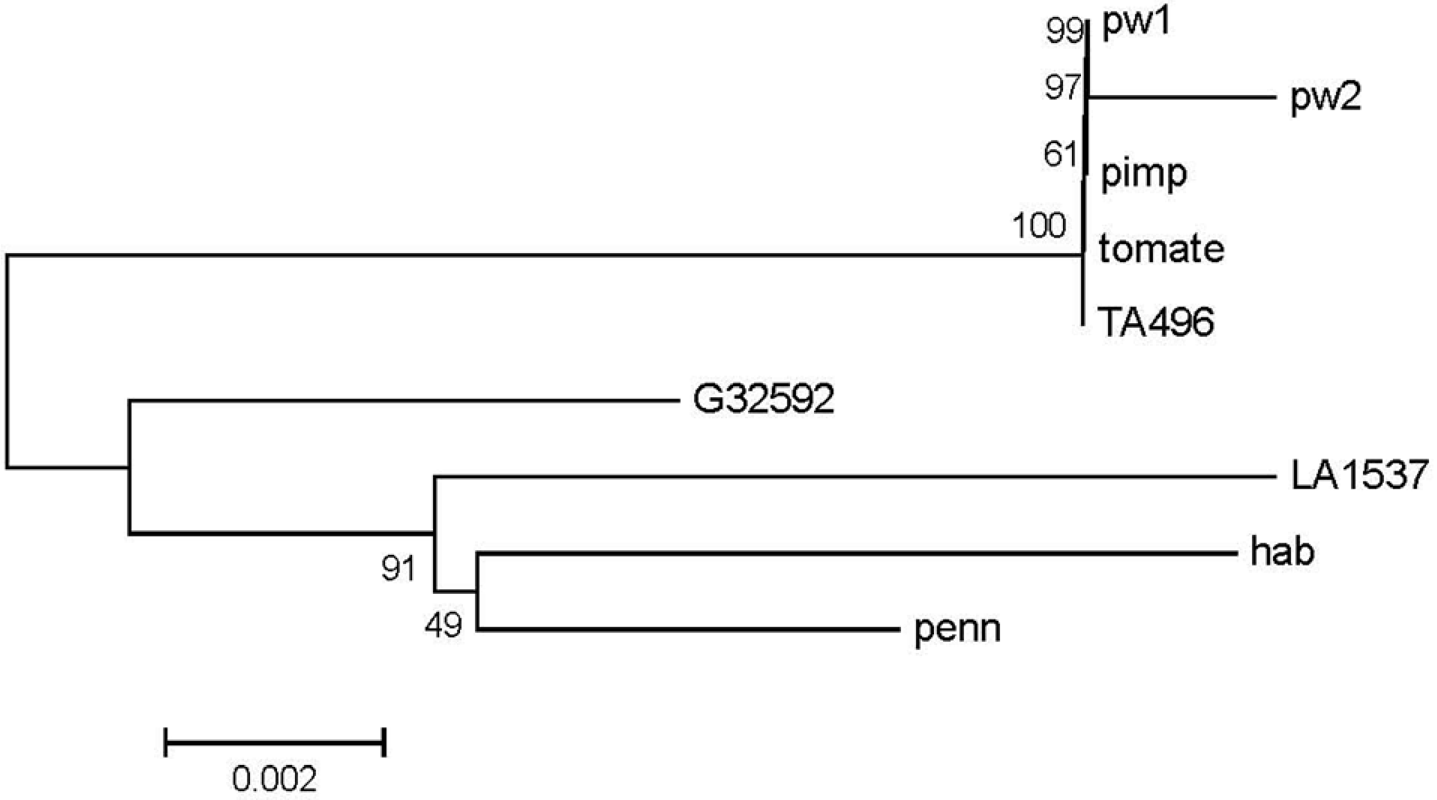

COS10
C2_At1g73180

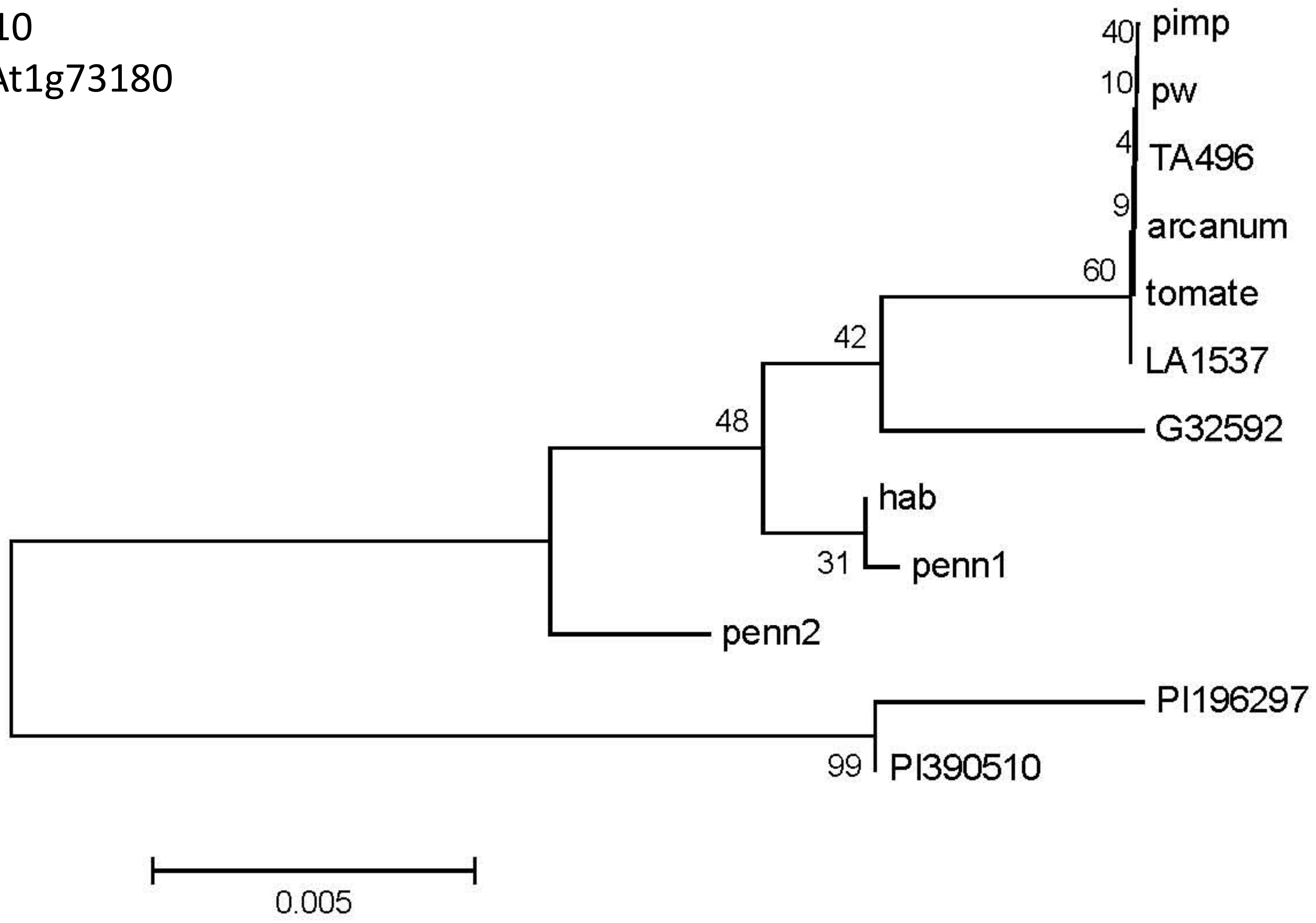

COS11
C2_At2g15890

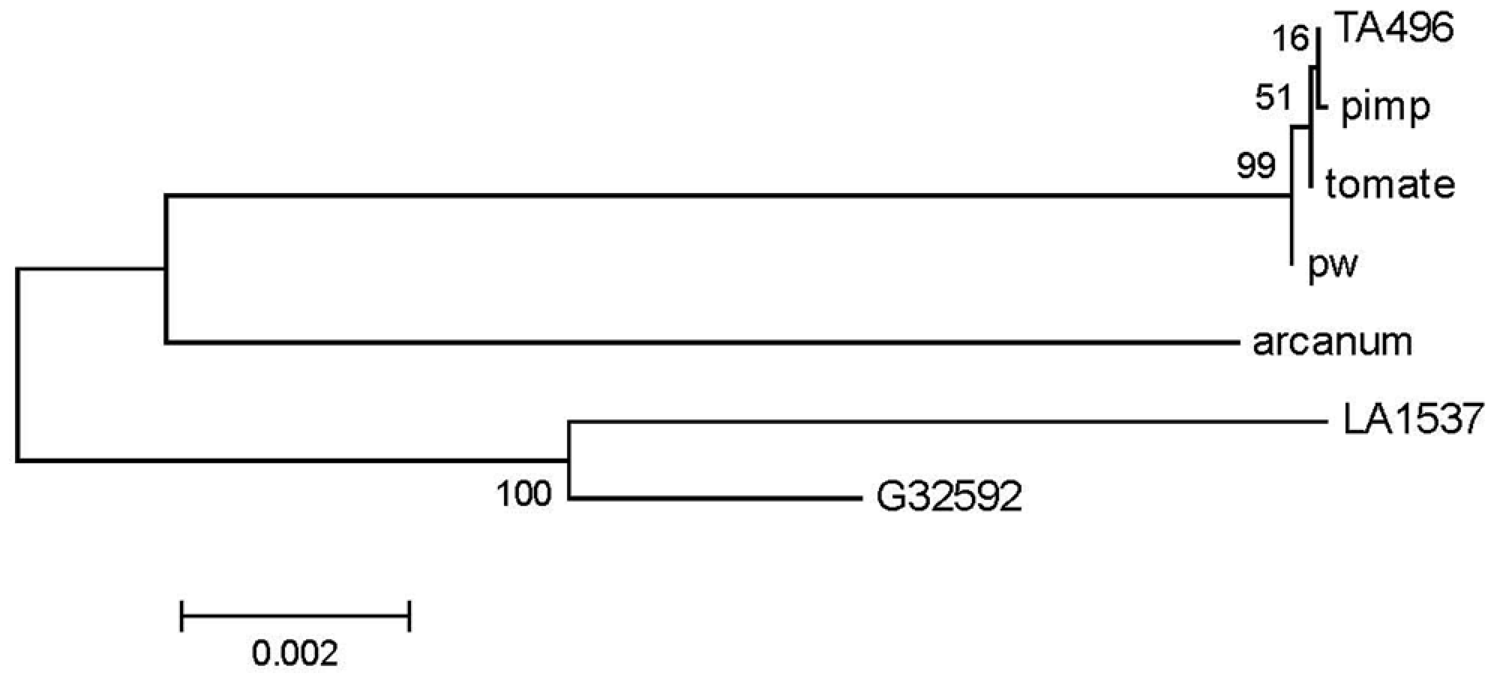

COS12
C2_At2g22570

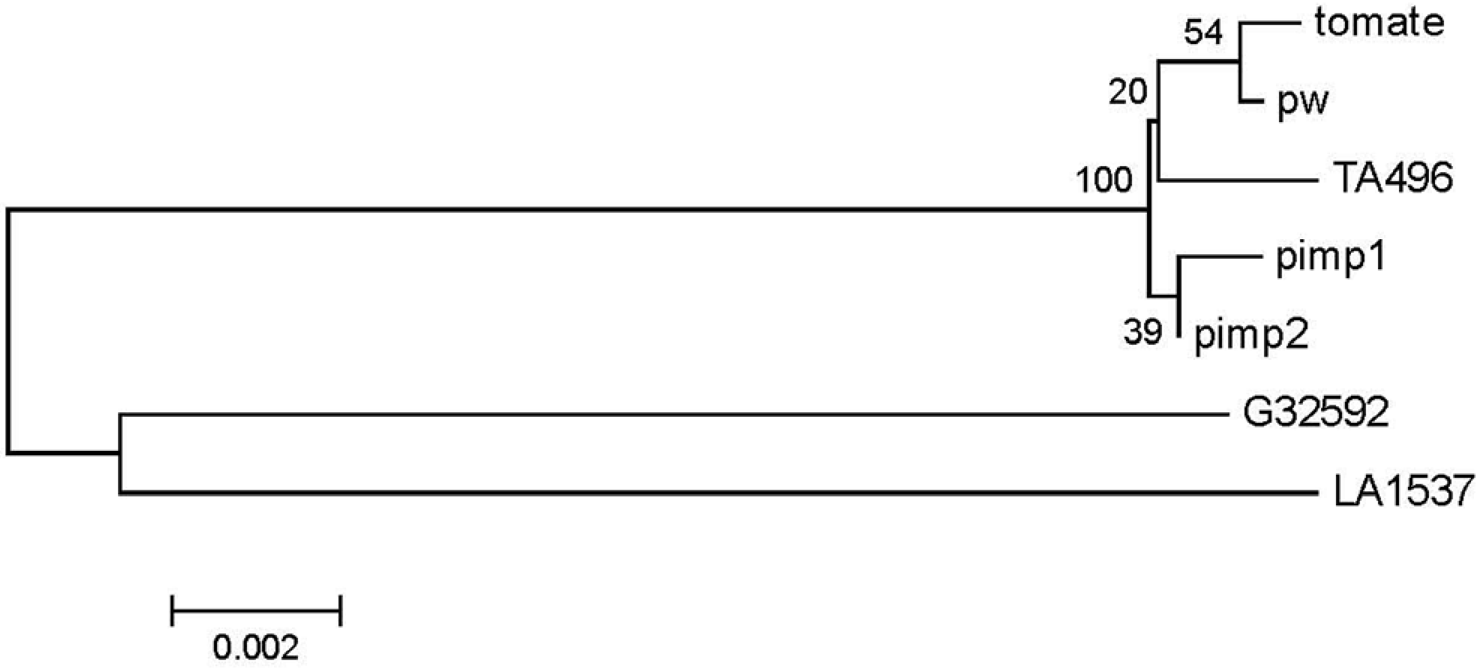

COS13
C2_At2g36930

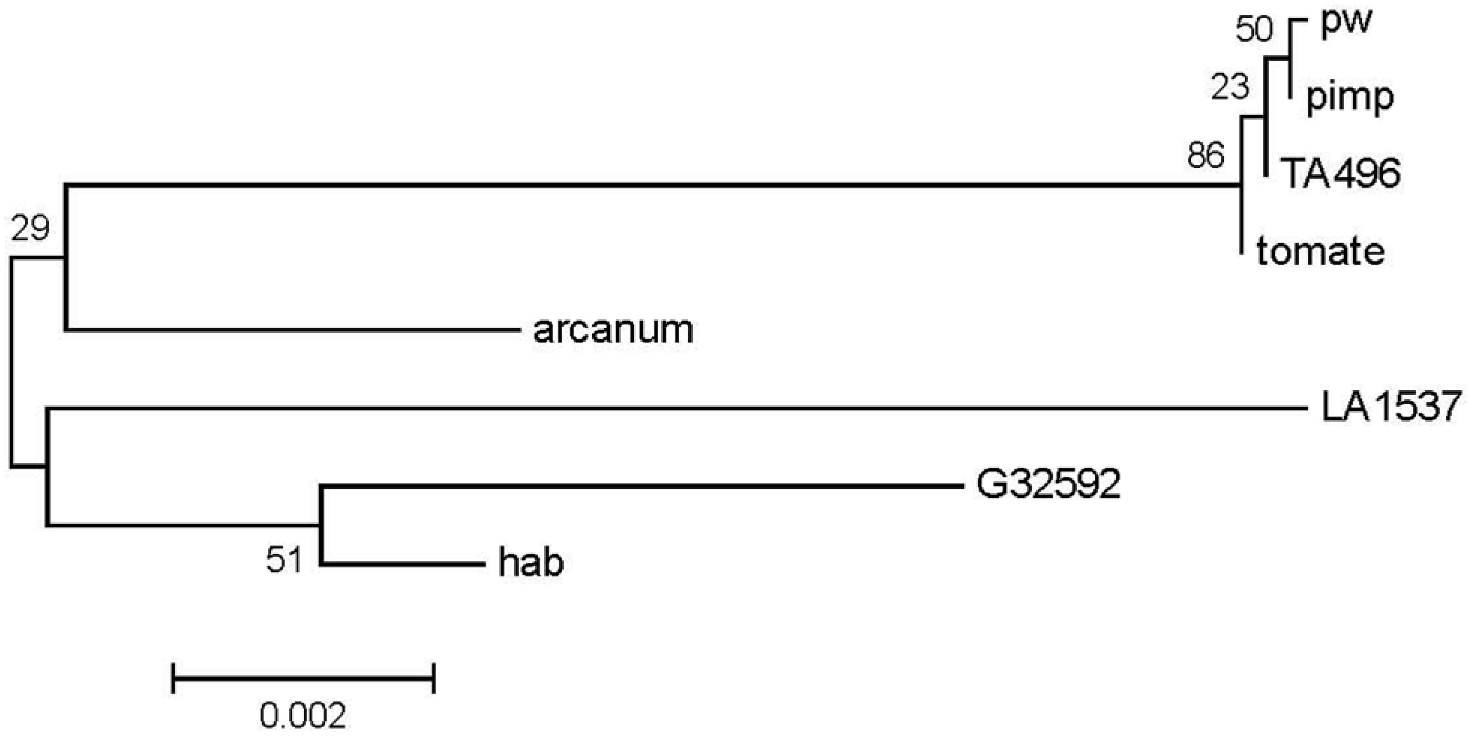

COS15
U221402

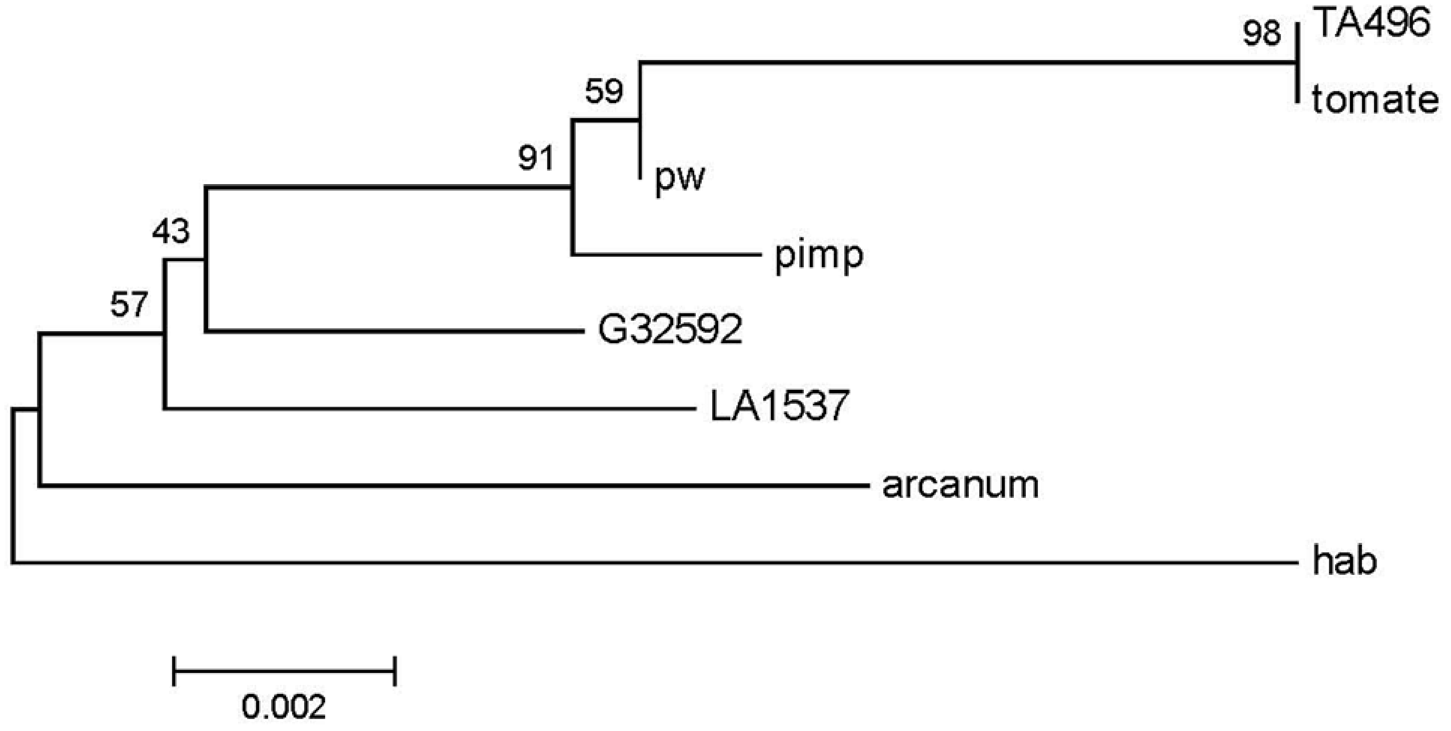

COS16
U318882

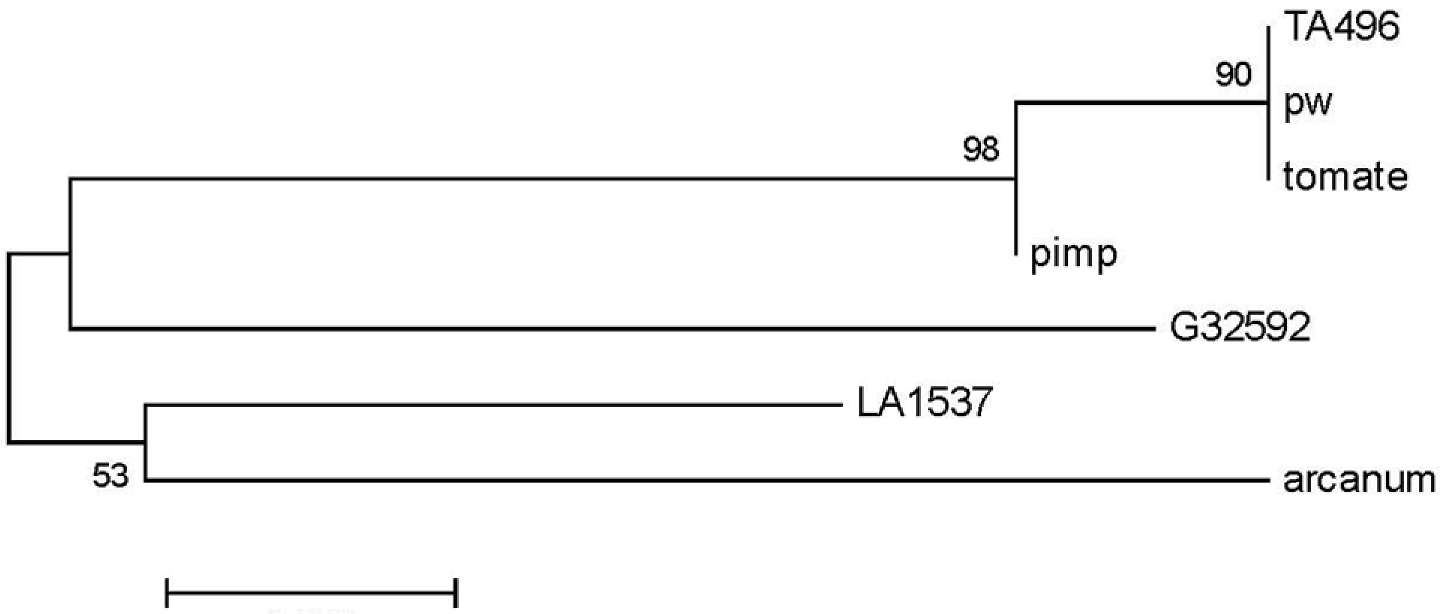

COS18
U146437

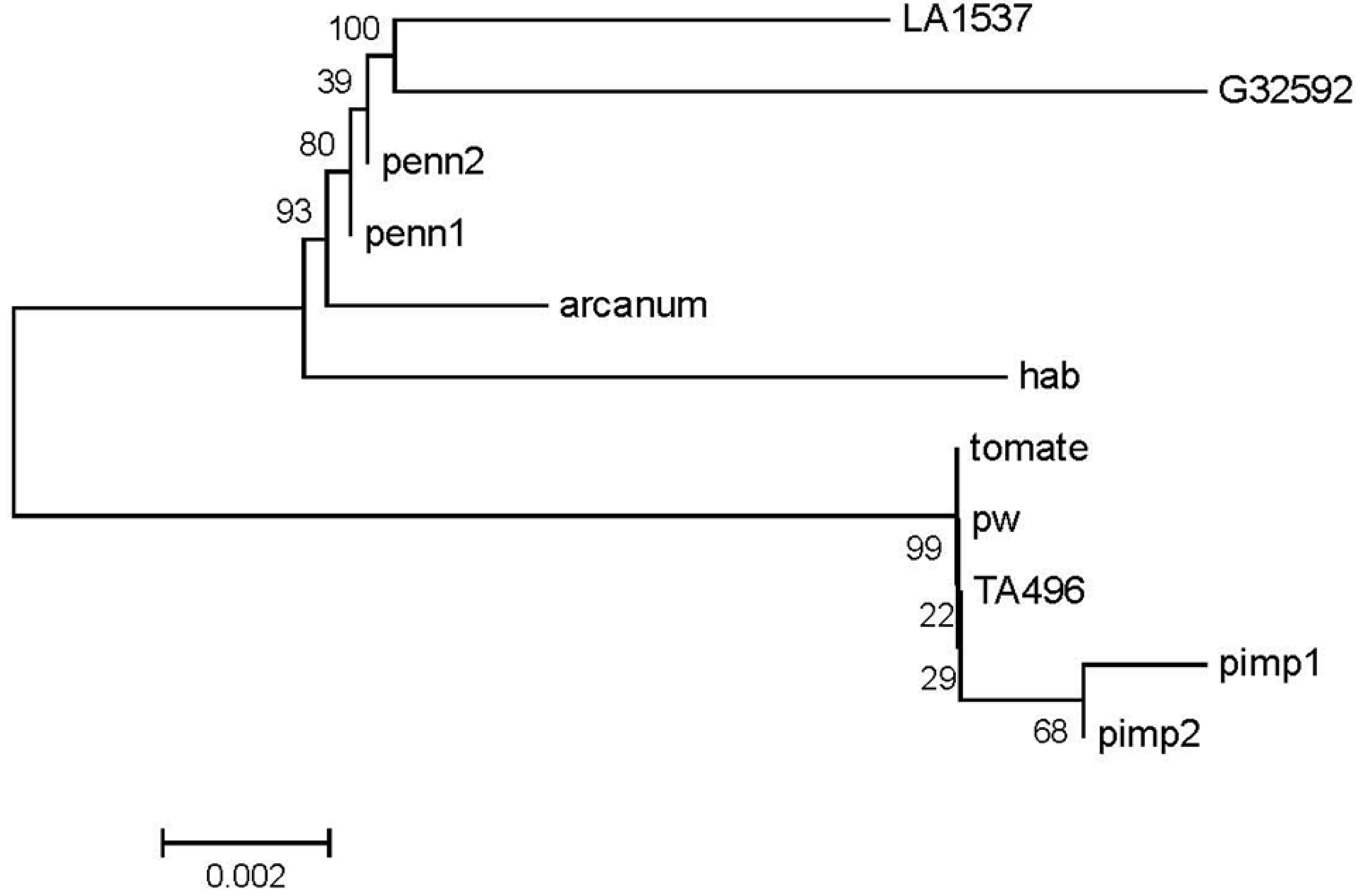

hp2 exon 2

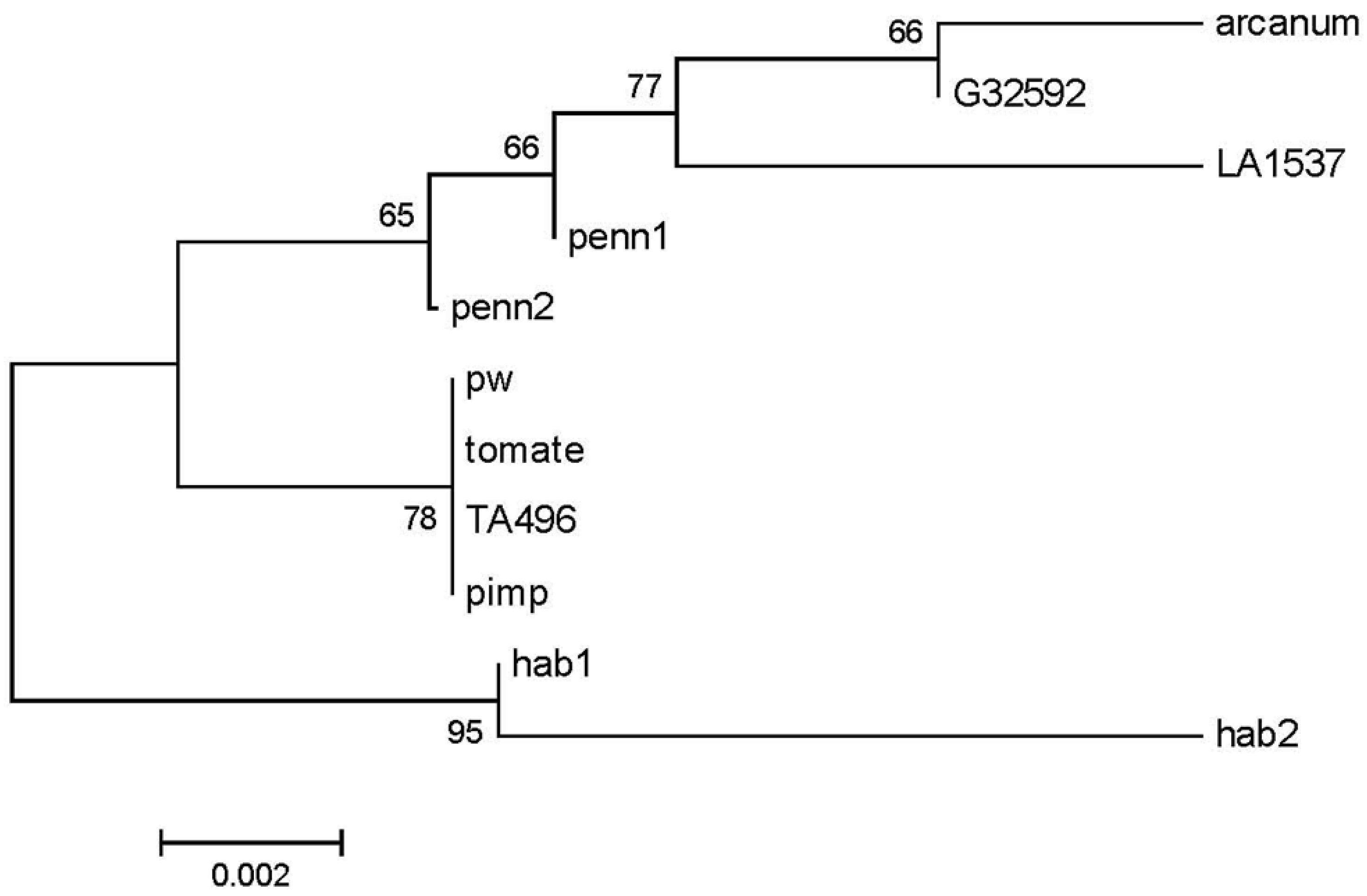
66
arcanum
77
G32592
66
LA1537
65
penn1
penn2
pw
tomate
78
TA496
pimp
hab1
95
hab2
0.002

Pds

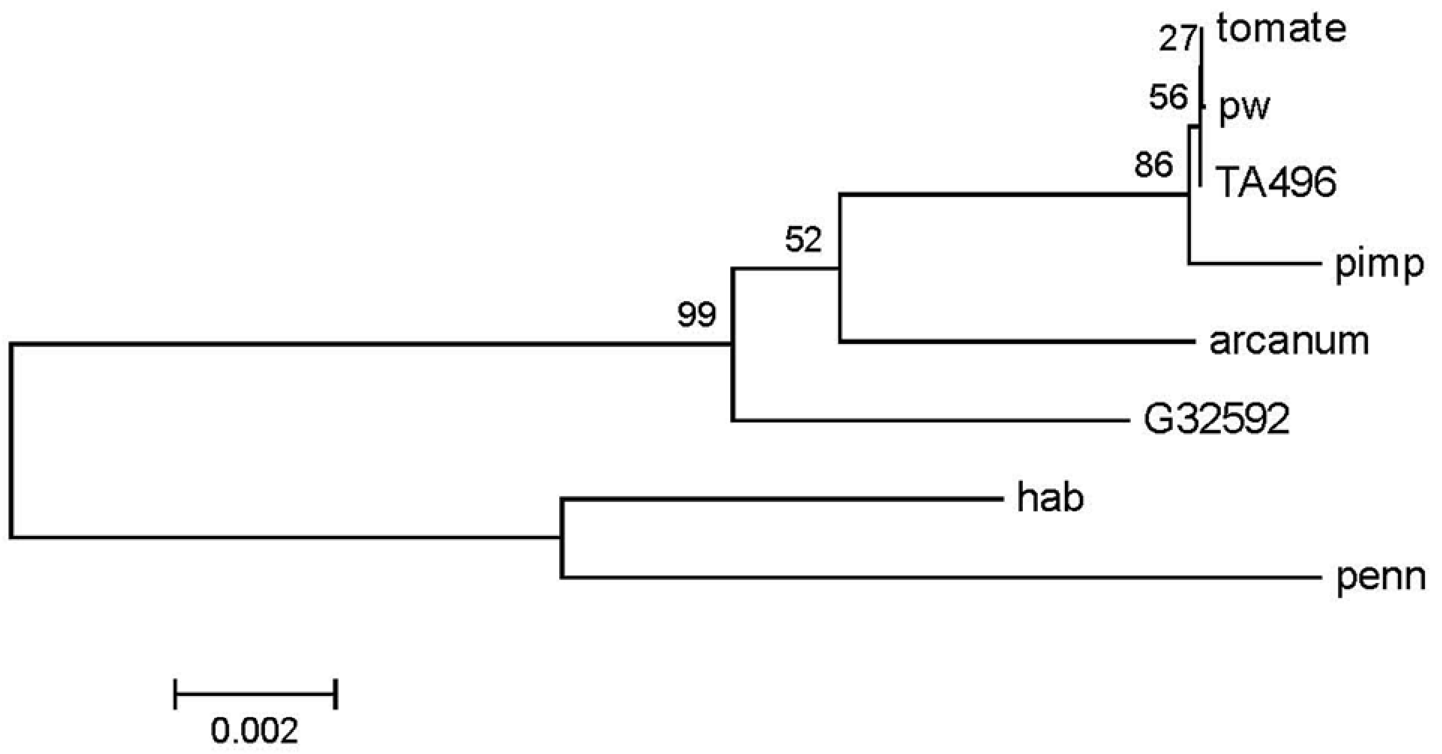
27
tomate
56
pw
86
TA496
52
pimp
99
arcanum
G32592
hab
penn
0.002

Psy1

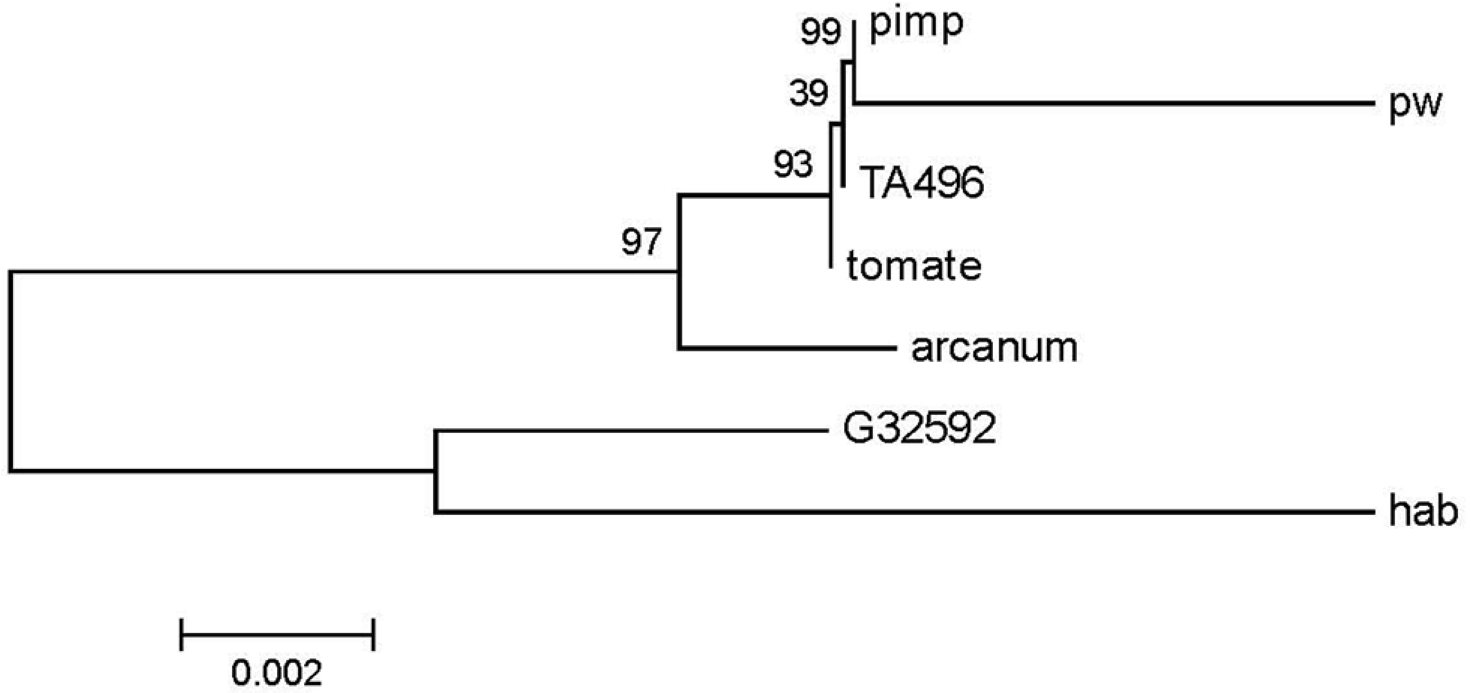

Cyc-B 5' region

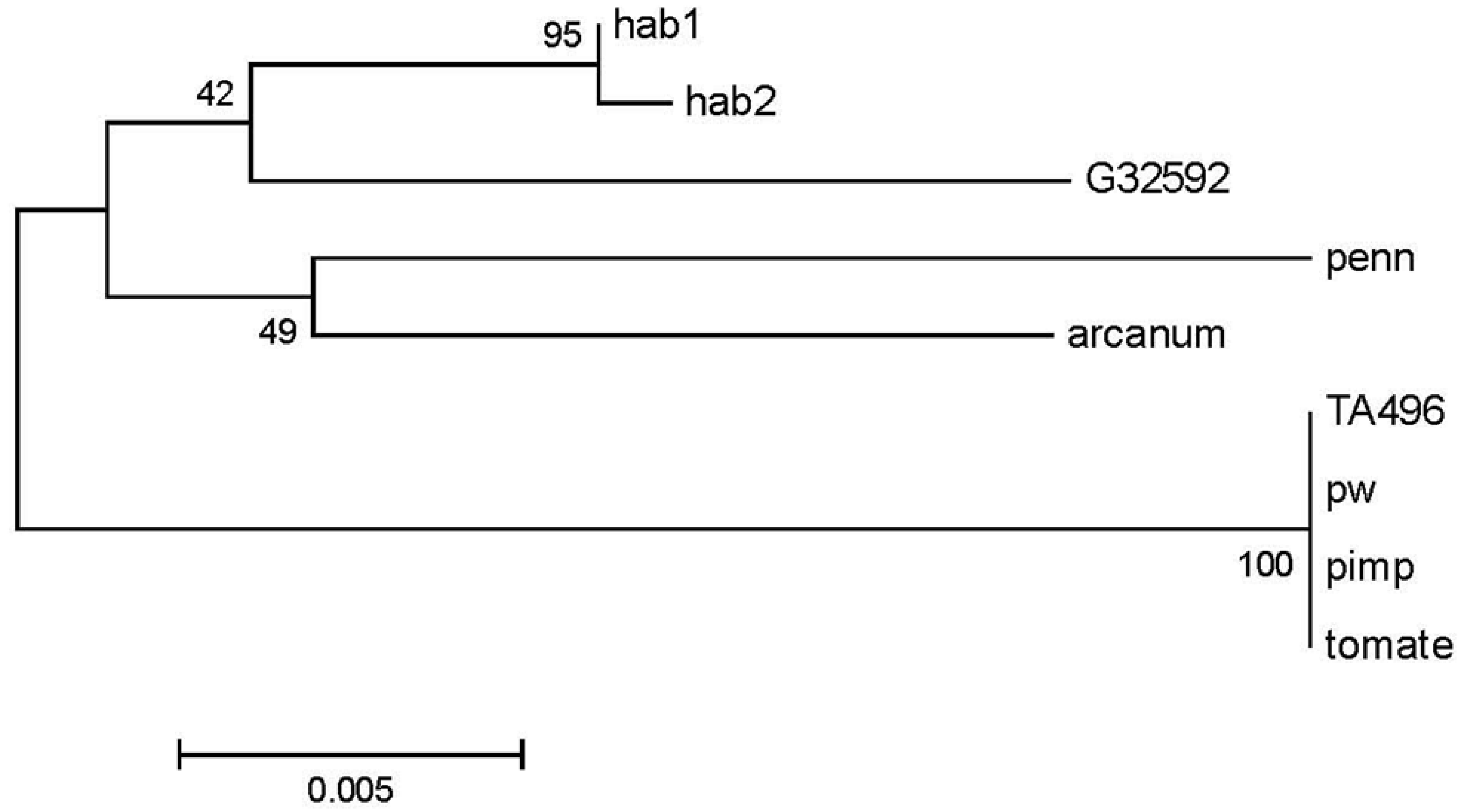

hp2 3’ region

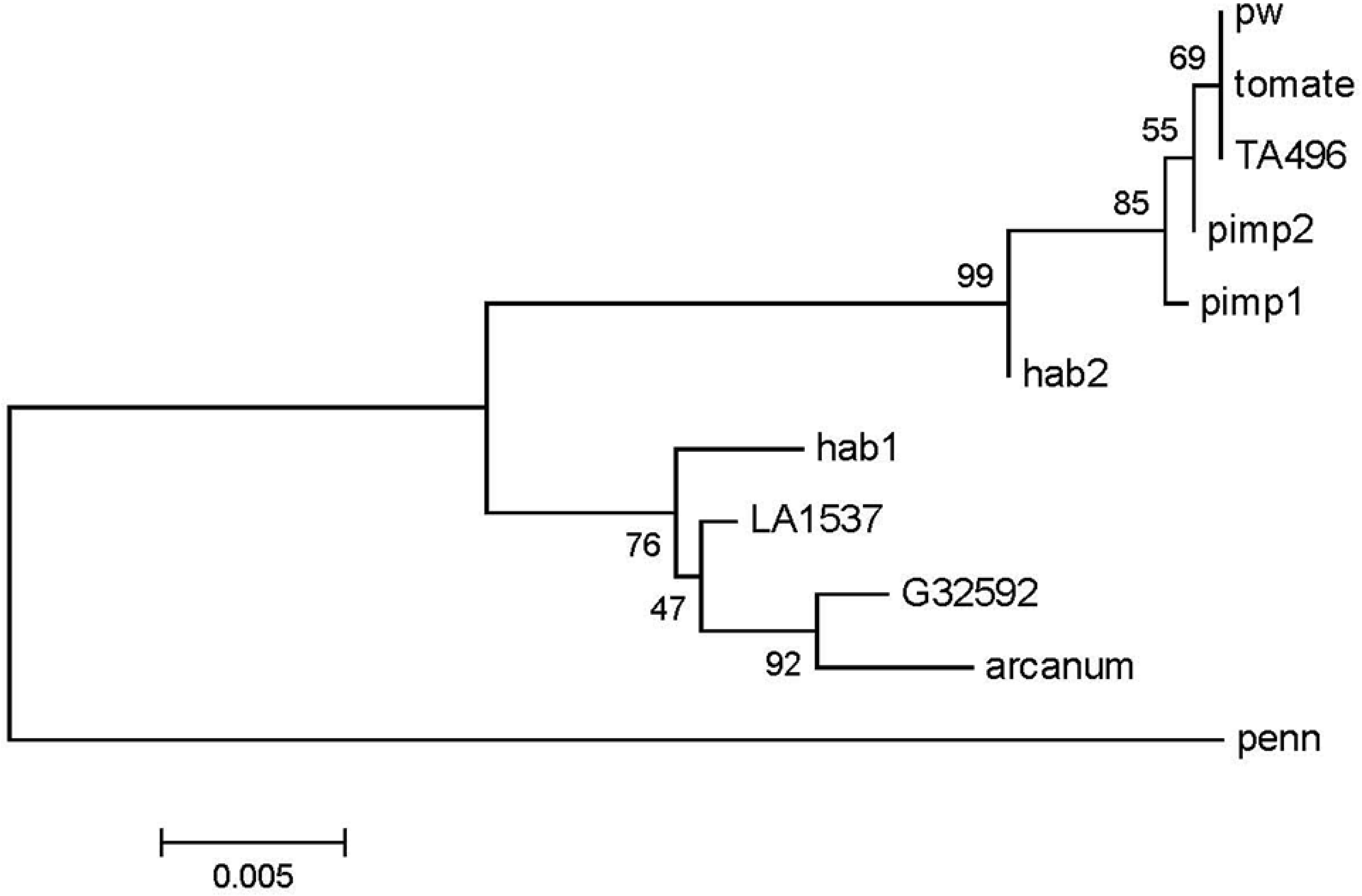
pw
69
tomate
55
TA496
85
pimp2
99
pimp1
hab2
hab1
LA1537
76
G32592
47
arcanum
92
penn
0.005

fw 2.2

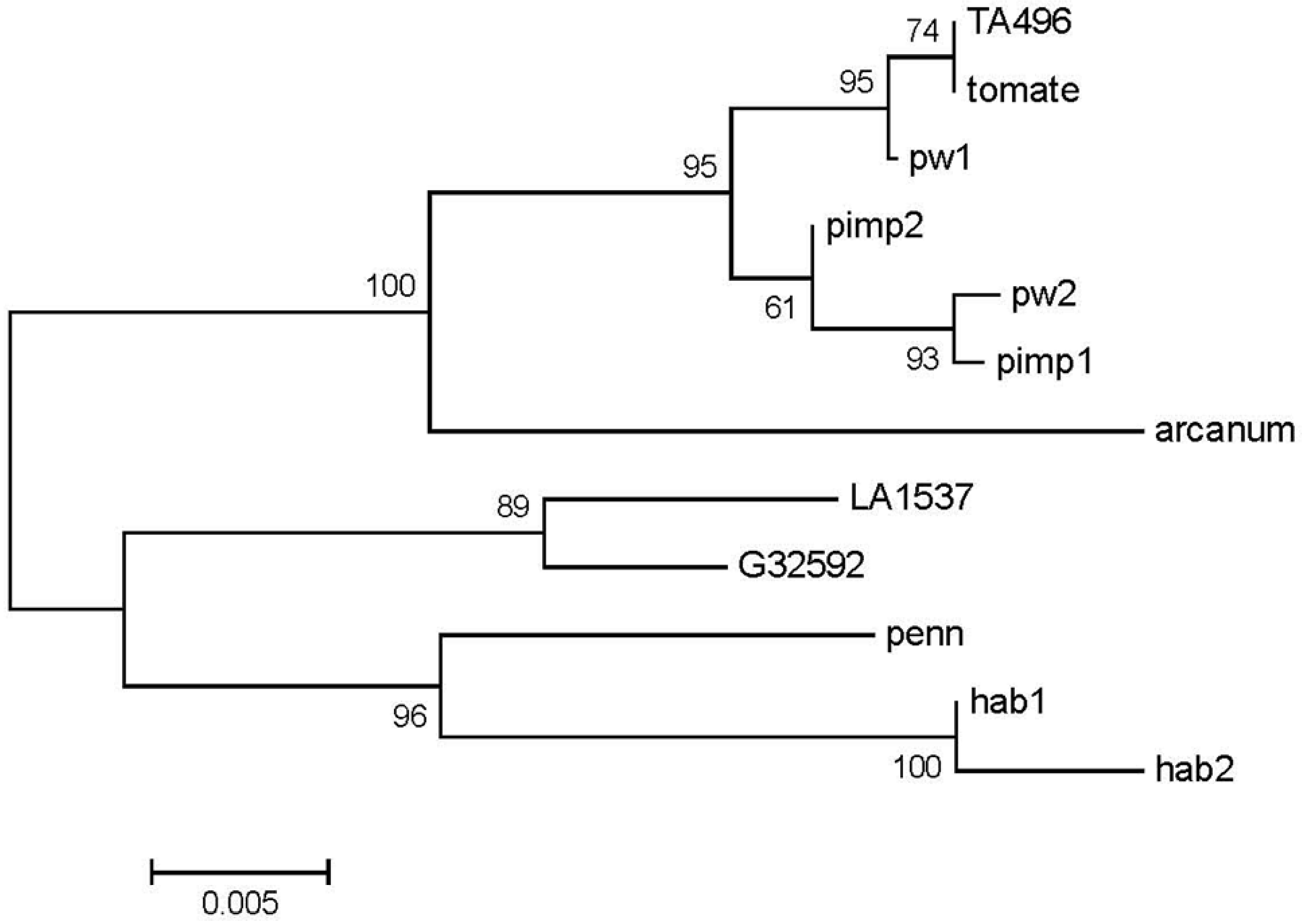

74
TA496
95
tomate
95
pw1
pimp2
100
pw2
61
93
pimp1
arcanum
89
LA1537
G32592
penn
96
hab1
100
hab2
0.005

TG11

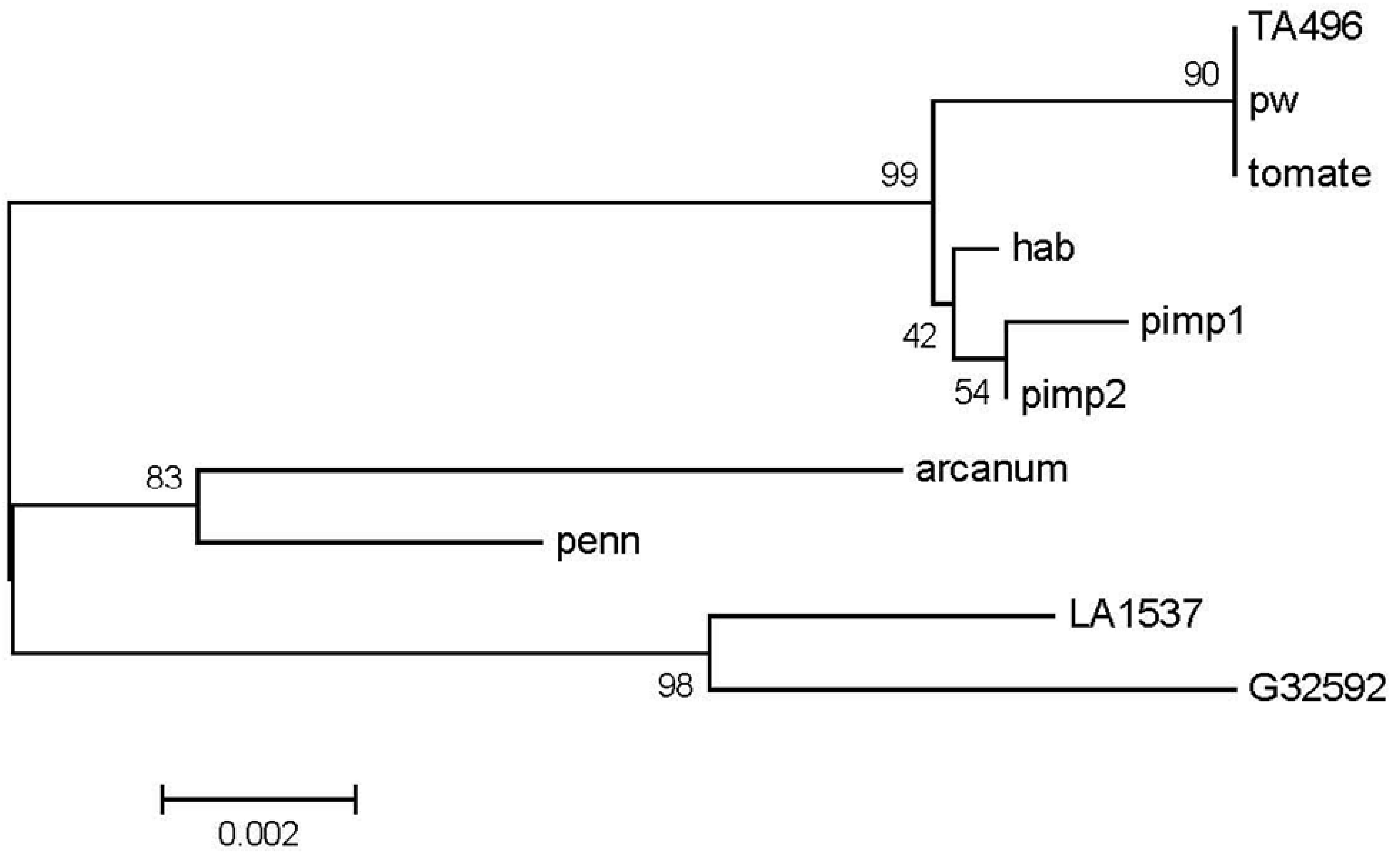
TA496
90
pw
tomate
99
hab
pimp1
42
54
pimp2
83
arcanum
penn
LA1537
98
G32592
0.002

CRTISO

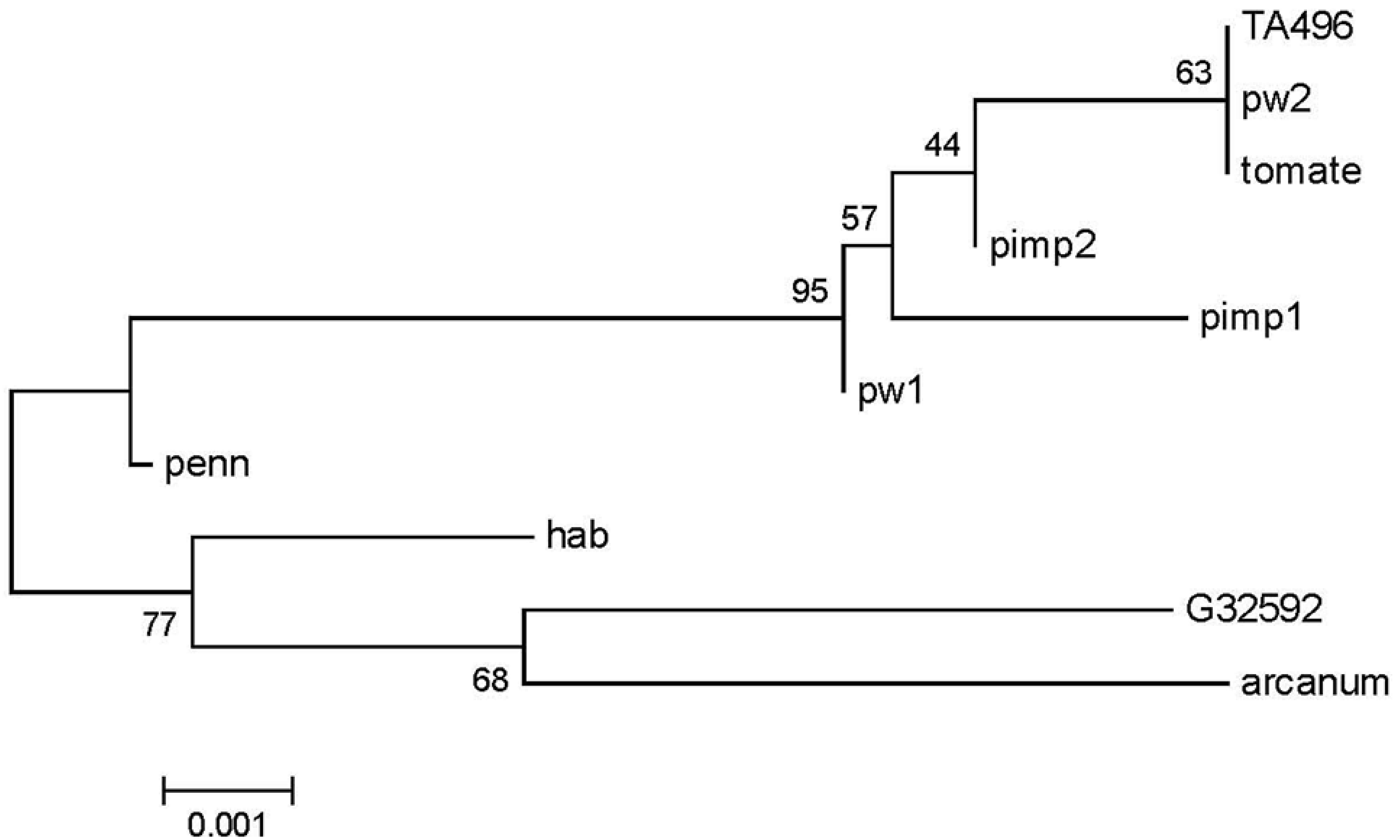
TA496
63
pw2
tomate
44
57
pimp2
95
pimp1
pw1
penn
hab
G32592
77
68
arcanum
0.001

rin

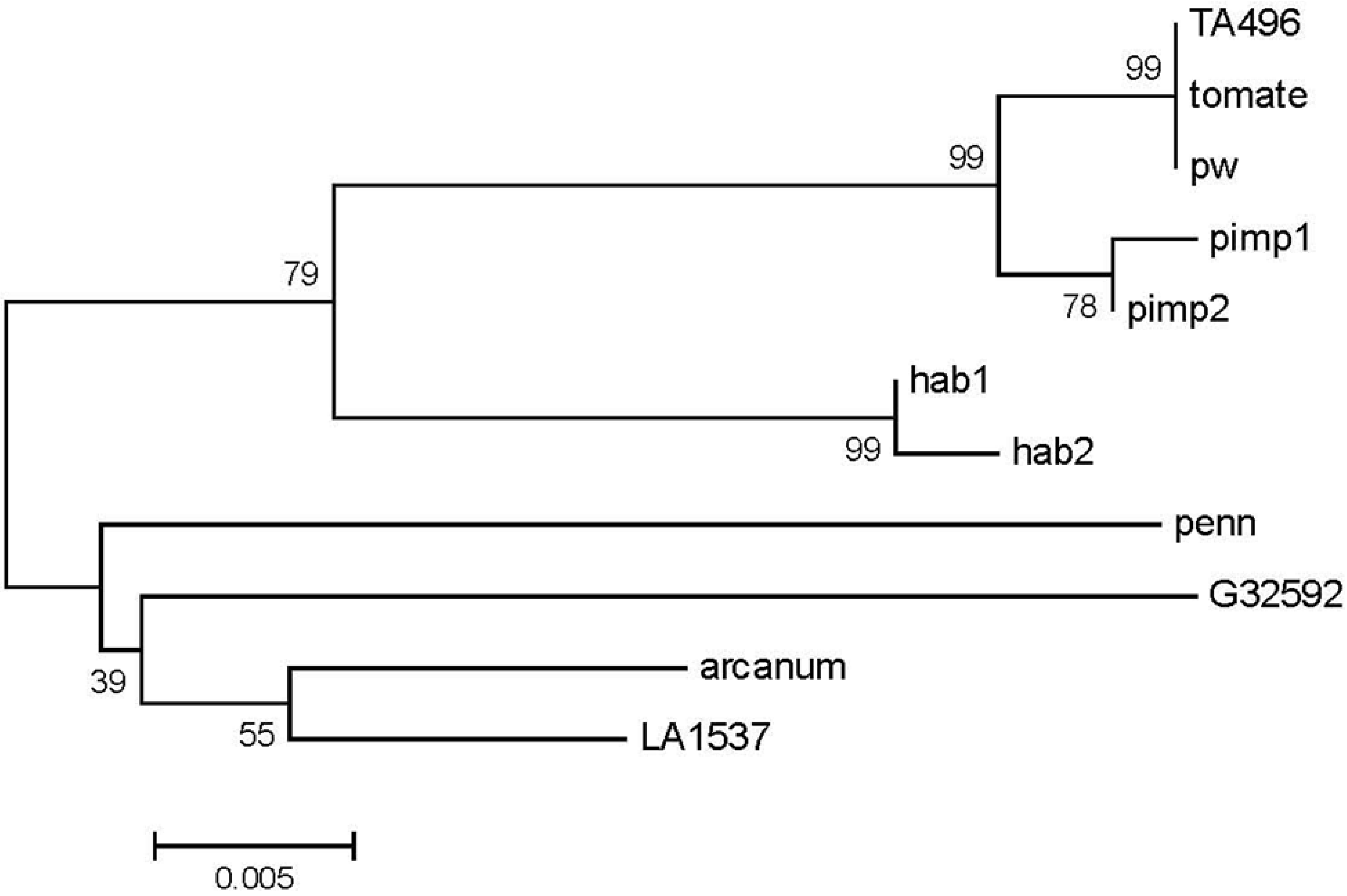
TA496
99
tomate
99
pw
pimp1
79
78
pimp2
hab1
99
hab2
penn
G32592
arcanum
39
55
LA1537
0.005

PTOX

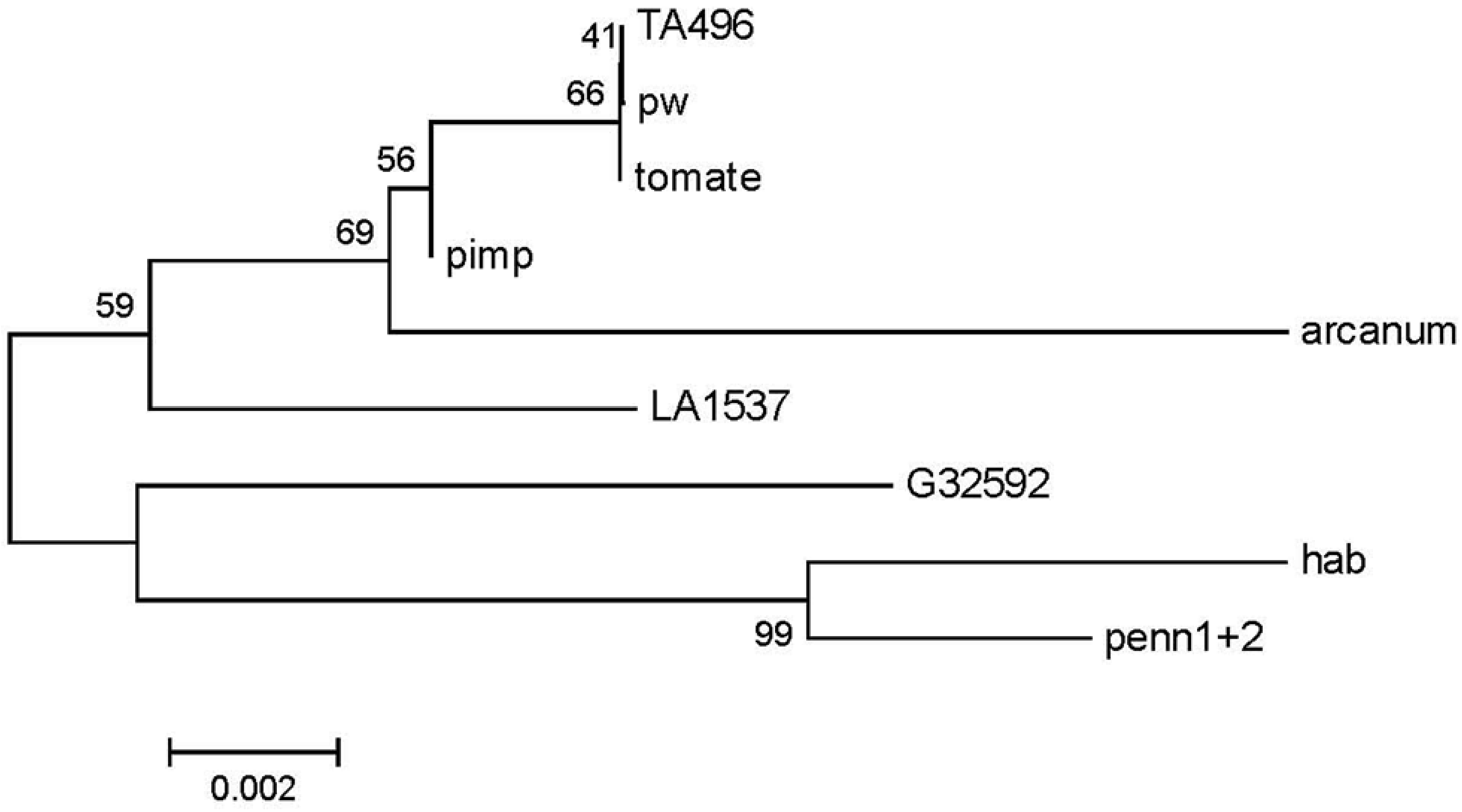

41
TA496
66
pw
56
tomate
69
pimp
59
arcanum
LA1537
G32592
hab
99
penn1+2
0.002

LeSNP30
2486_1

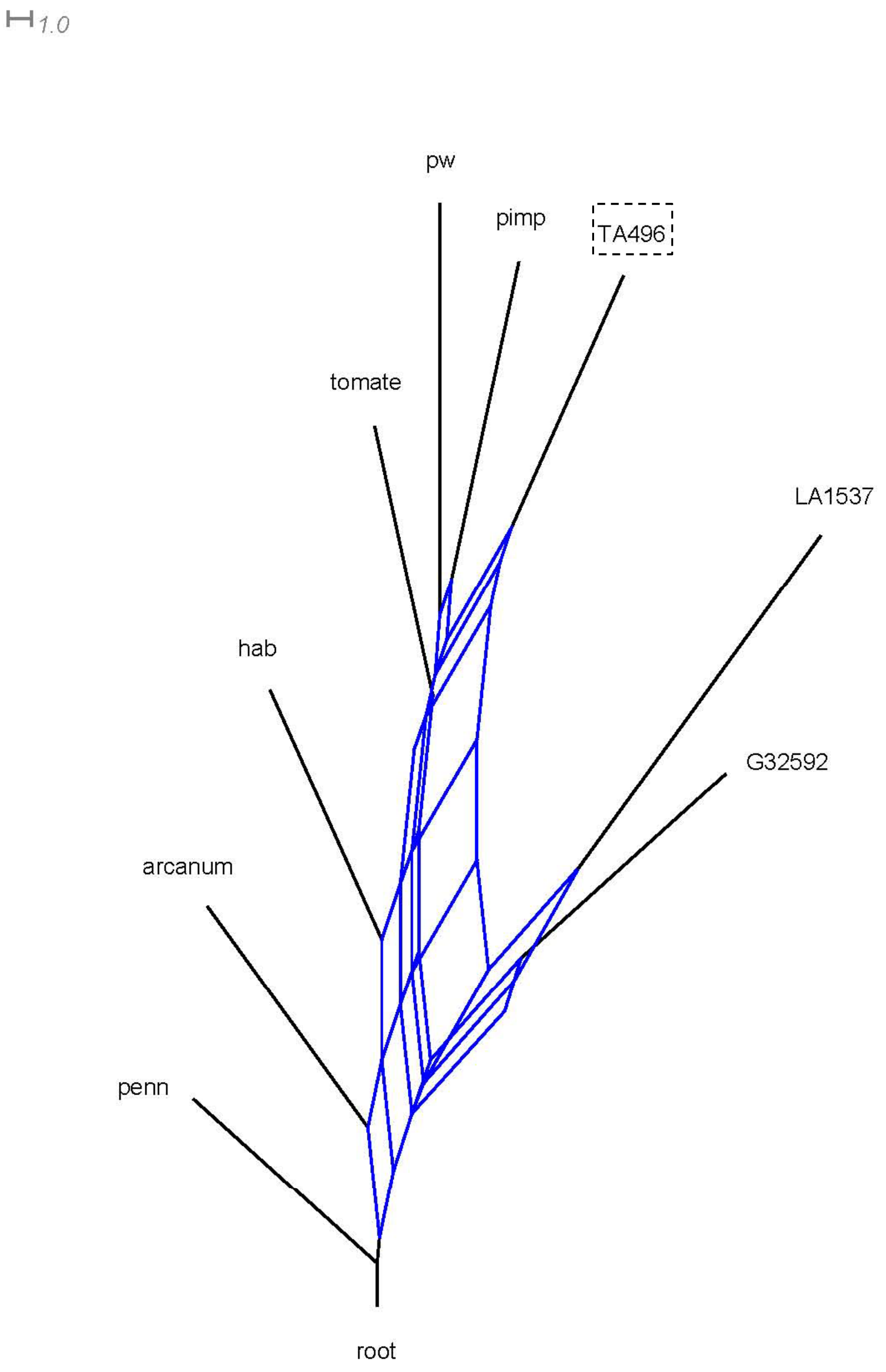

LeSNP9b
2534_1b

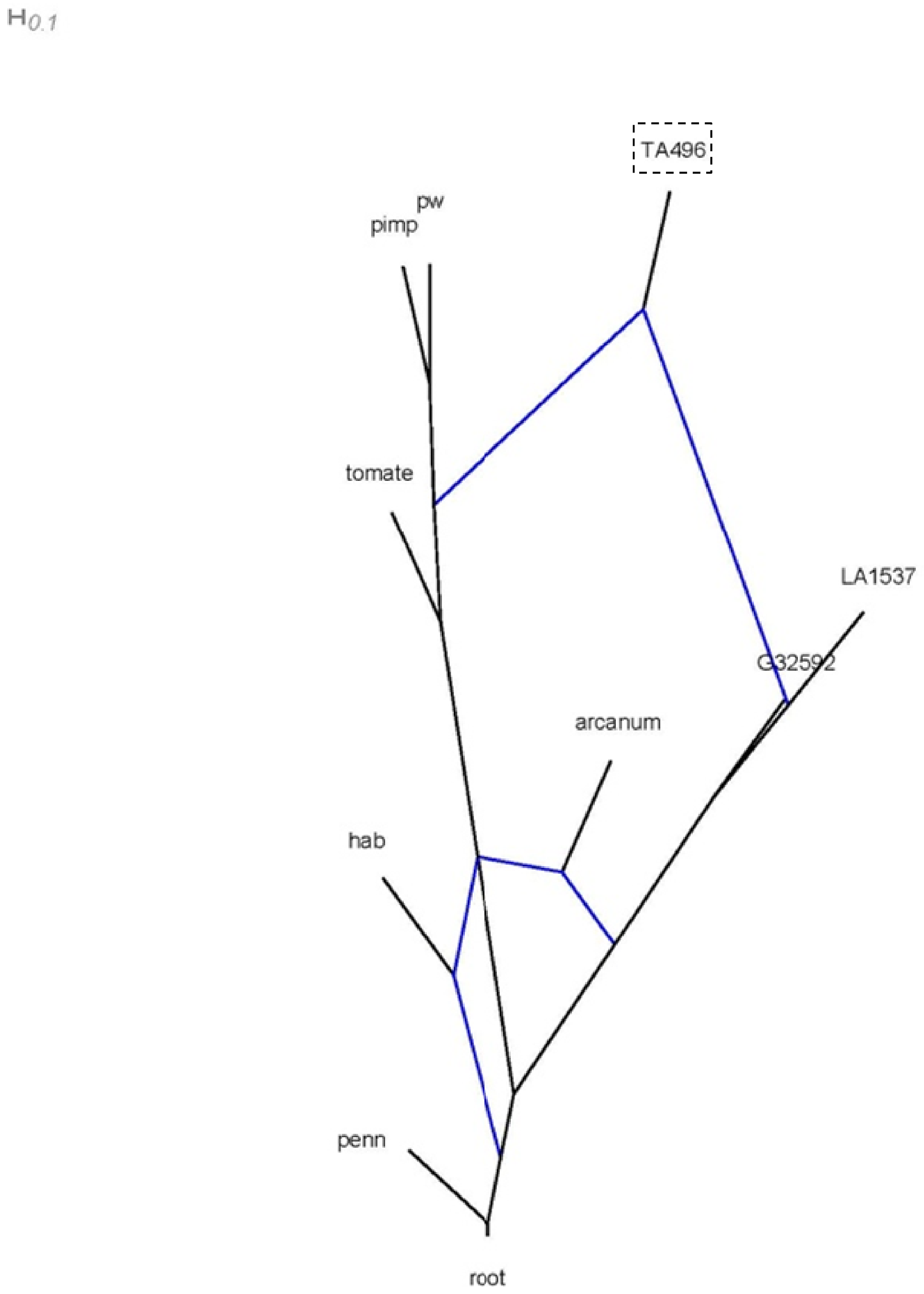

LeSNP25
220_1

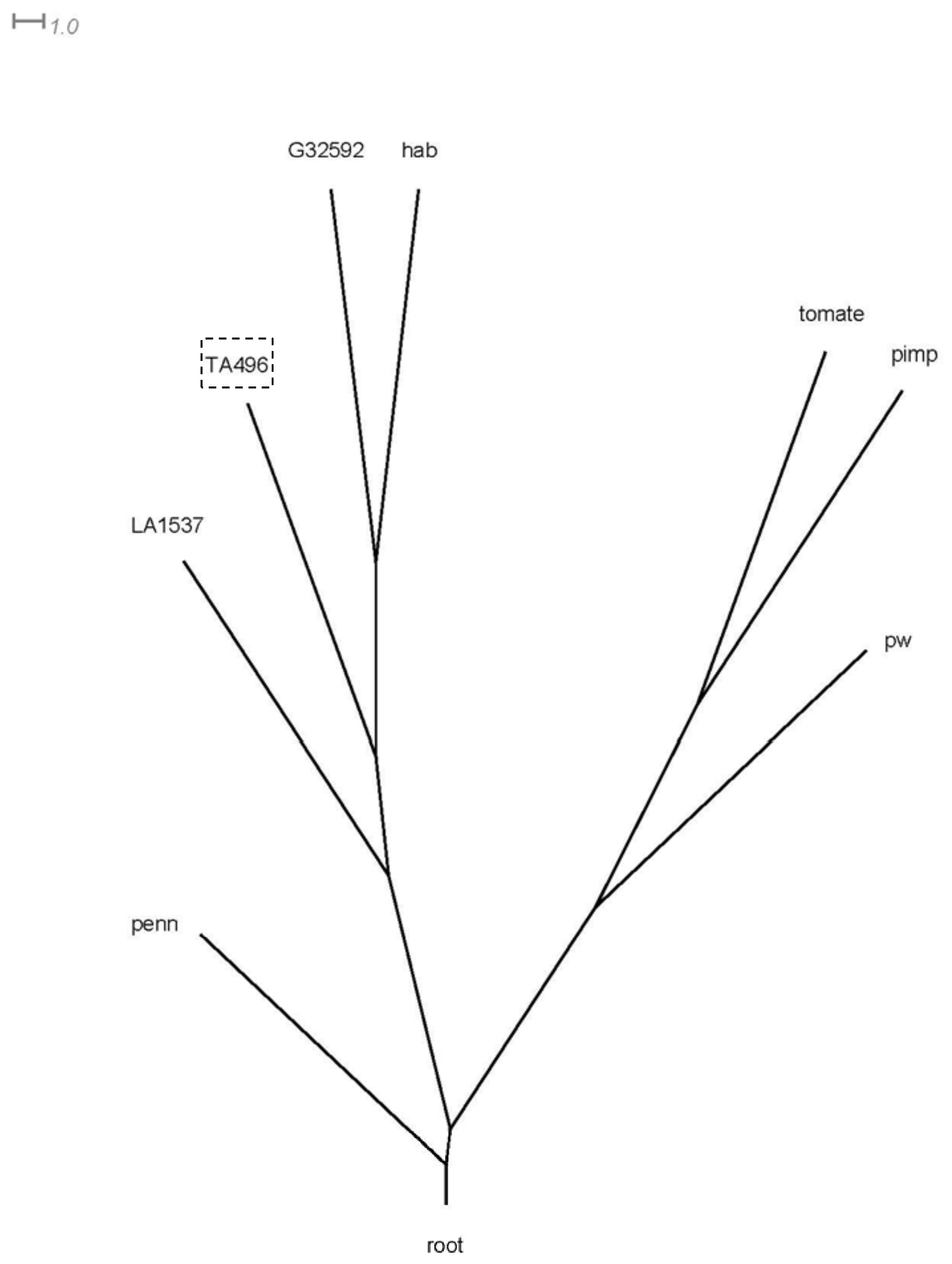

LeSNP1
437_2

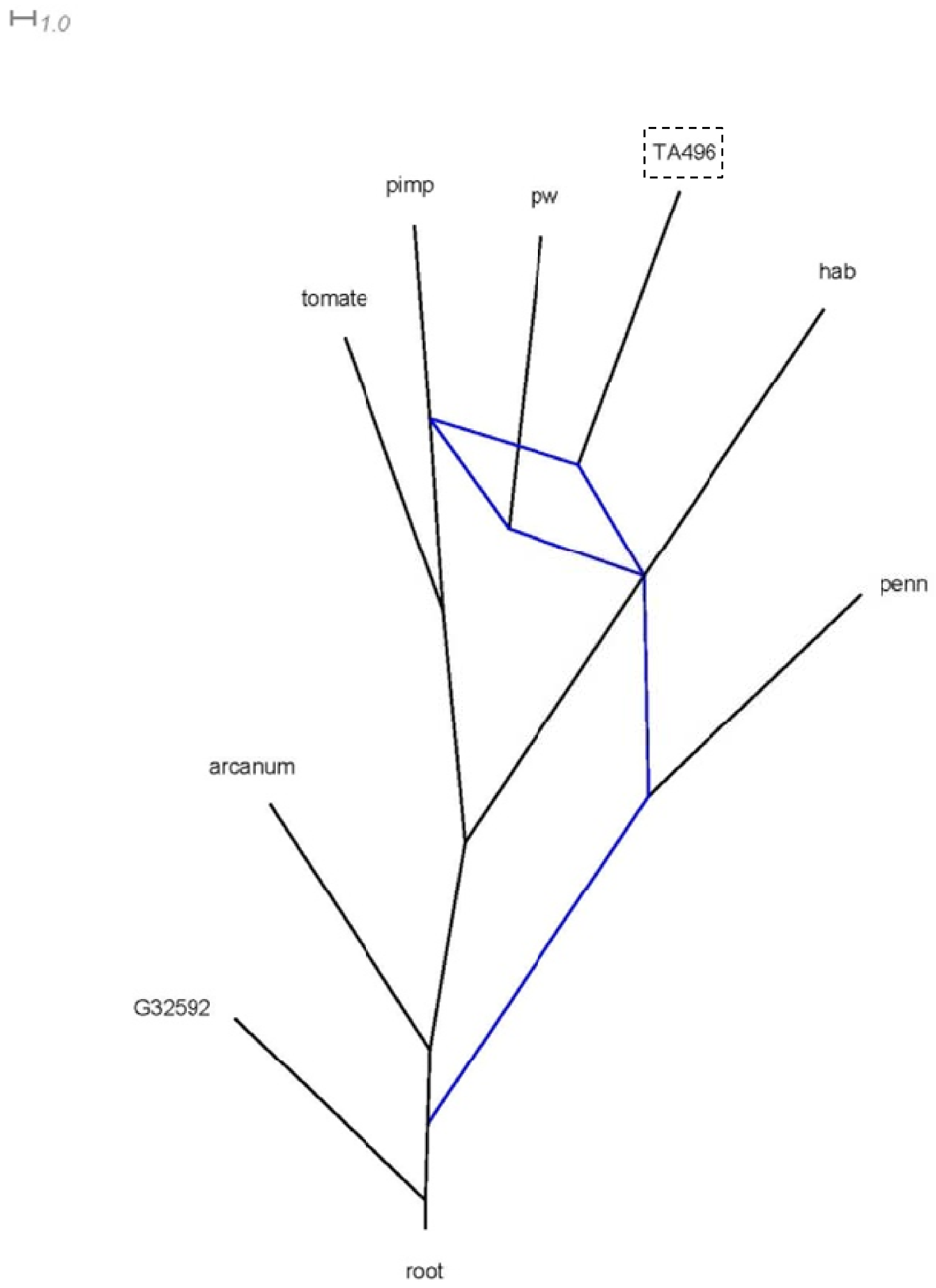

LeSNP10
2325_3

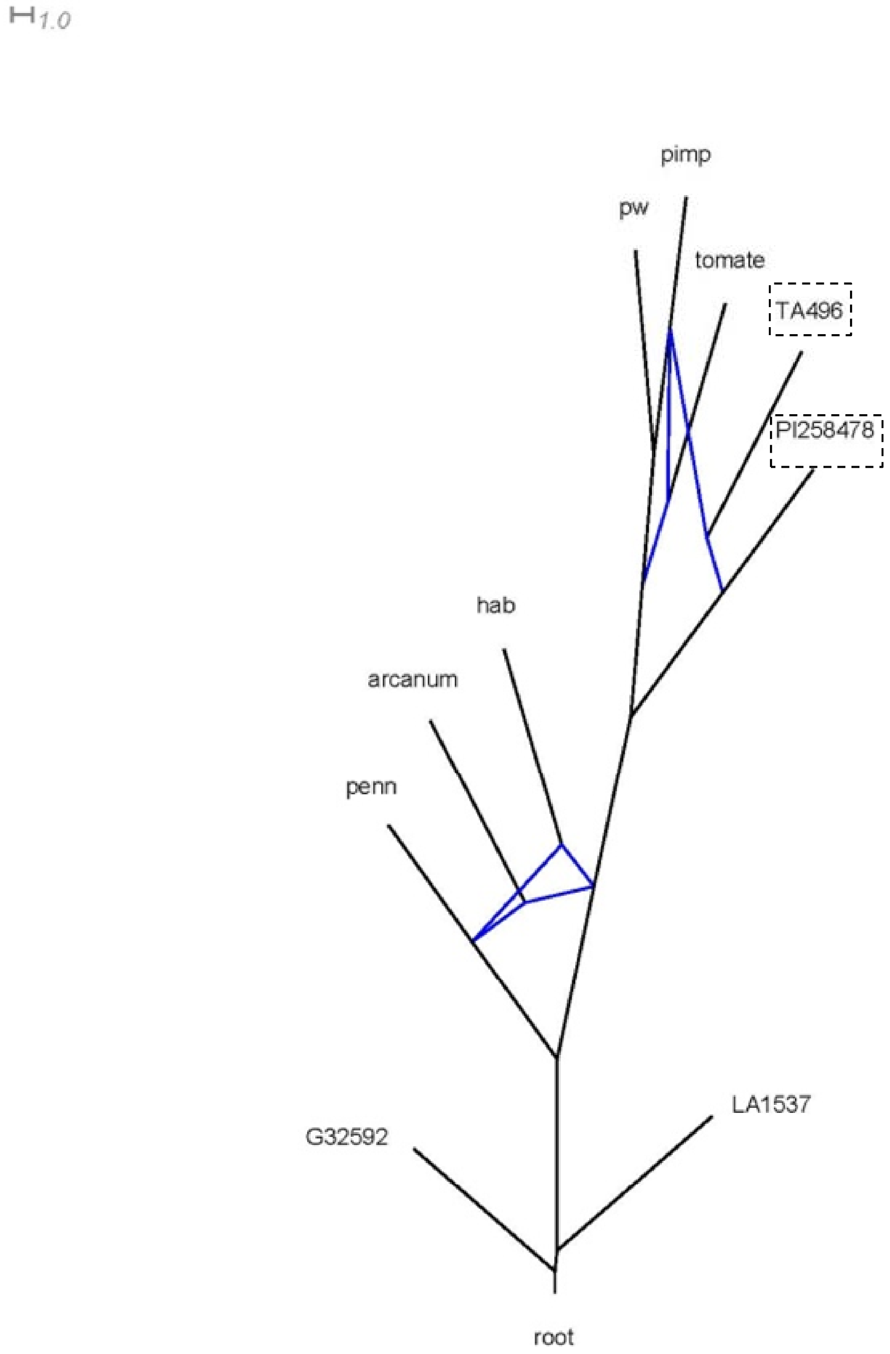

COS7
C2_At1g44575

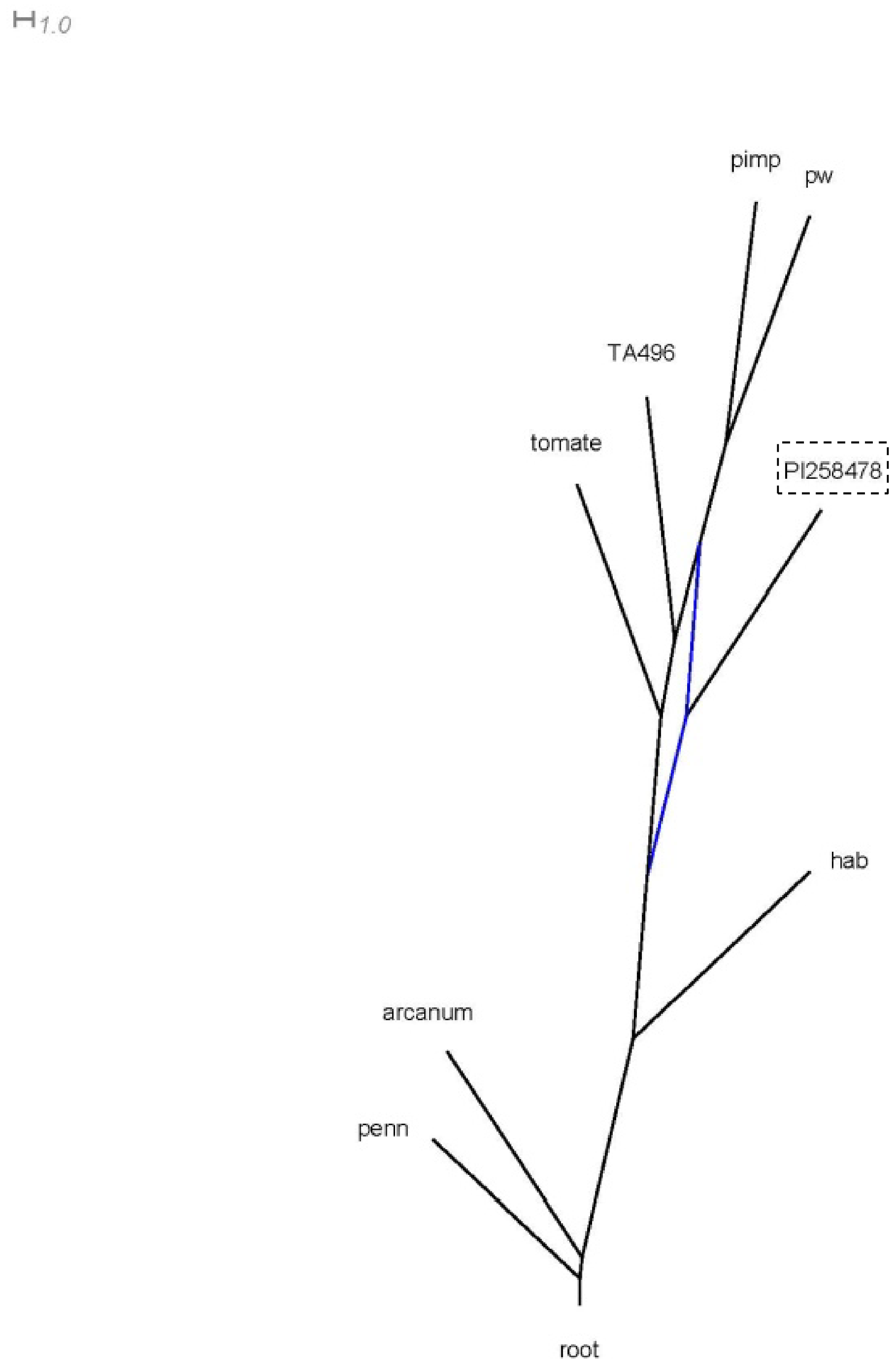

LeSNP34
2819_5

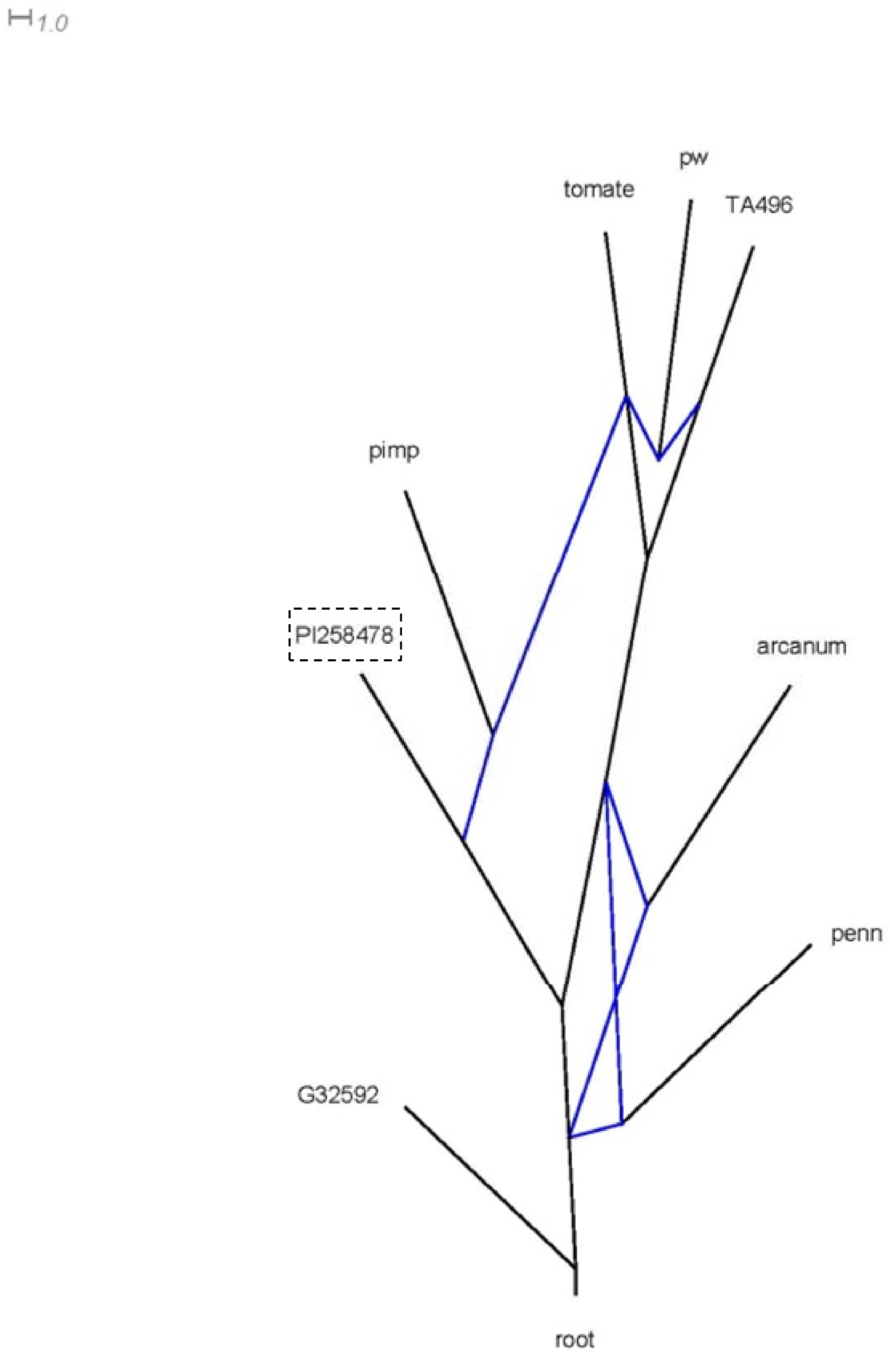

COS1
U146140

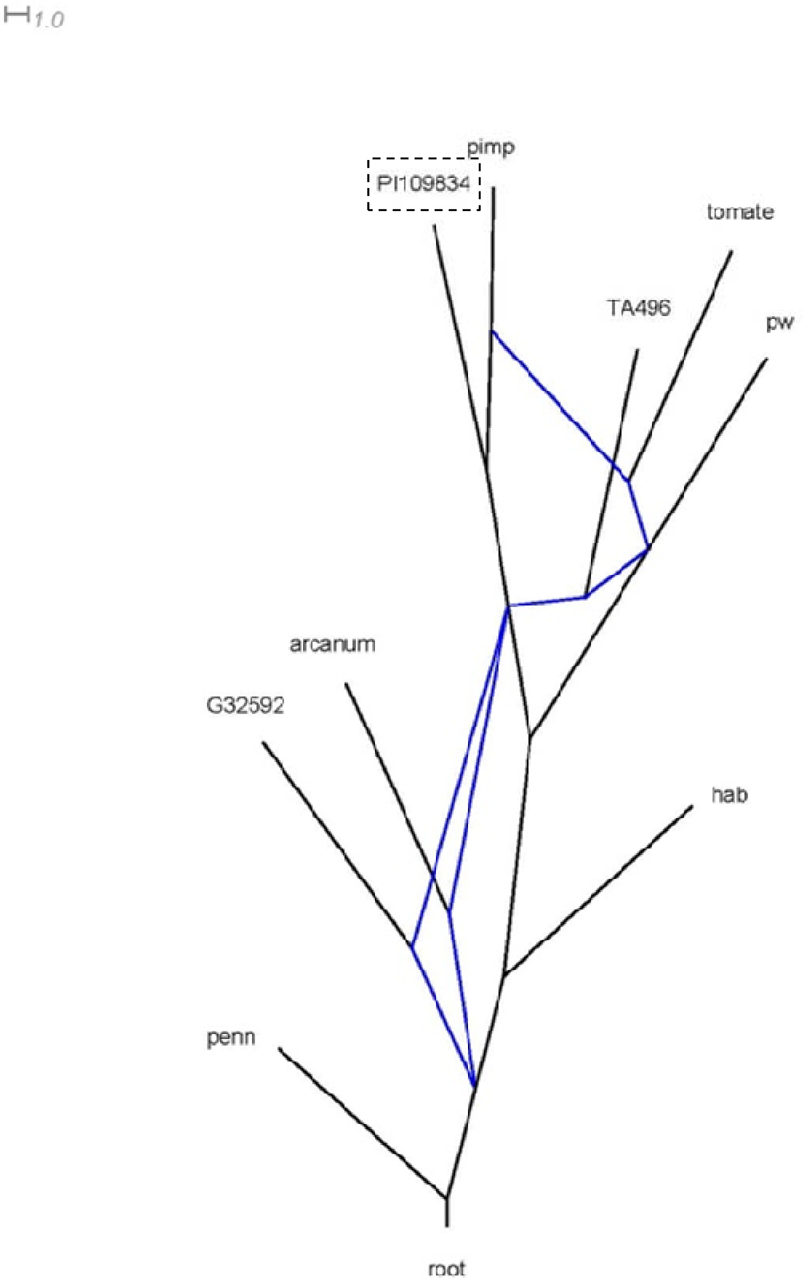

COS10
C2_At1g73180

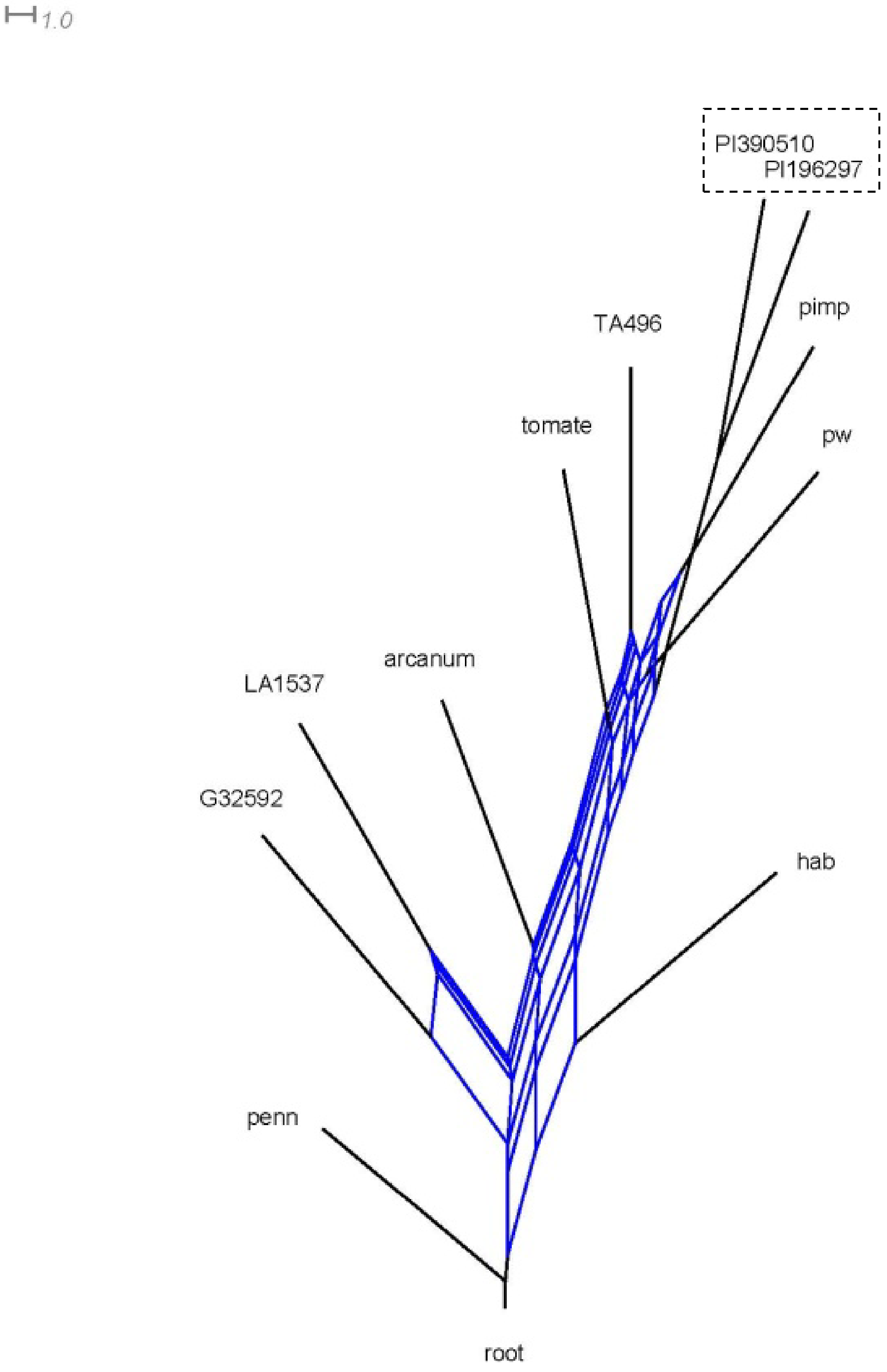

Supplement: Additional file 2 — Clustering and network analyses of tomato genotypes. Neighbor joining trees for 47 markers and hybridization networks for nine markers sampled from wild and cultivated tomato. [file 1471-2229-12-133-S2.pdf]
